# Spatial learning impairments and discoordination of entorhinal-hippocampal circuit coding following prolonged febrile seizures

**DOI:** 10.1002/hipo.23541

**Published:** 2023-04-24

**Authors:** Michelle L. Kloc, Yuncai Chen, Jennifer M. Daglian, Gregory L. Holmes, Tallie Z. Baram, Jeremy M. Barry

**Affiliations:** 1 Epilepsy Cognition and Development Group, Department of Neurological Sciences, University of Vermont, Larner College of Medicine, Burlington, Vermont, USA; 2 Departments of Pediatrics, University California-Irvine, Irvine, California, USA; 3 Departments of Anatomy/Neurobiology, University California-Irvine, Irvine, California, USA; 4 Departments of Neurology, University California-Irvine, Irvine, California, USA

**Keywords:** circuit throughput, development, febrile status epilepticus, network development, pathology, seizures, spatial memory

## Abstract

How the development and function of neural circuits governing learning and memory are affected by insults in early life remains poorly understood. The goal of this study was to identify putative changes in cortico-hippocampal signaling mechanisms that could lead to learning and memory deficits in a clinically relevant developmental pathophysiological rodent model, Febrile status epilepticus (FSE). FSE in both pediatric cases and the experimental animal model, is associated with enduring physiological alterations of the hippocampal circuit and cognitive impairment. Here, we deconstruct hippocampal circuit throughput by inducing slow theta oscillations in rats under urethane anesthesia and isolating the dendritic compartments of CA1 and dentate gyrus subfields, their reception of medial and lateral entorhinal cortex inputs, and the efficacy of signal propagation to each somatic cell layer. We identify FSE-induced theta-gamma decoupling at cortical synaptic input pathways and altered signal phase coherence along the CA1 and dentate gyrus somatodendritic axes. Moreover, increased DG synaptic activity levels are predictive of poor cognitive outcomes. We propose that these alterations in cortico-hippocampal coordination interfere with the ability of hippocampal dendrites to receive, decode and propagate neocortical inputs. If this frequency-specific syntax is necessary for cortico-hippocampal coordination and spatial learning and memory, its loss could be a mechanism for FSE cognitive comorbidities.

## 1 | INTRODUCTION

How early-life neurological insults alter the operation and coordination of oscillations within and between circuits underlying cognition remains poorly understood (Barry & Holmes, 2016; Barry et al., 2016). Febrile seizures (FS) are one of the most common forms of seizure in early life, occurring in 2%–5% of American and Western European infants and children (Baram & Shinnar, 2001; Chungath & Shorvon, 2008; Steering Committee on Quality & Management, 2008). Whereas short FS are benign (Chang et al., 2003; Kloc et al., 2021), a subset of these seizures is prolonged, and when lasting 30 min or longer are termed Febrile status epilepticus (FSE). FS evolve into FSE in 5%–9% of children, making it the most frequent cause of status epilepticus in children (Berg & Shinnar, 1996; Shinnar et al., 2008). In human studies, FSE is a significant risk factor for hippocampal injury, temporal lobe epilepsy, and enduring cognitive impairments (Hesdorffer et al., 2011; Lewis et al., 2014; Martinos et al., 2012; Scott, 2010, 2014; Shinnar et al., 1990; Shinnar et al., 2008; Verity et al., 1998; Weiss et al., 2016). Given the clinical importance of FSE and its comorbidities, animal models have been developed that mimic its risks for epilepsy and the development of cognitive deficit (Chen et al., 2001; Chen et al., 2021; Dube et al., 2000; Dube et al., 2009; Dubé & Baram, 2006; Heida et al., 2004). Animals experiencing this model of experimental Febrile status epilepticus (eFSE) tend to exhibit enduring spatial memory impairments (Barry et al., 2016; Barry et al., 2020; Kloc et al., 2022; Patterson et al., 2017) that correlate with an absence of CA1 place cell modulation by theta oscillations, poor theta and gamma coordination between CA1 and CA3, and poor functional CA1 gamma in relation to behavior (Barry et al., 2015, 2016, 2020; Dube et al., 2009). These effects could ultimately be attributed to eFSE-induced CA1 attenuation of apical dendritic branching, and the dentate gyrus granule cell (DGC) apical dendritic overgrowth and preservation of vestigial basal dendrites (Patterson et al., 2017). Yet, how these physiological alterations affect mechanisms of neocortical-hippocampal coordination have not been studied. Addressing the functional consequences of these FSE-induced CA1 and DG alterations is important as the efficacy of neocortical-hippocampal signal coordination has been proposed to be critical for spatial learning and memory (Bellmund et al., 2020; Fernandez-Ruiz et al., 2021; Gerlei et al., 2021; Igarashi et al., 2014; Lopez-Madrona et al., 2020; Niedecker et al., 2021; Olafsdottir et al., 2016; Quirk et al., 2021; Roy et al., 2017; Suh et al., 2011). The operational frequencies of current oscillations along the somatodendritic axes of hippocampal neurons may represent a code or syntax for the processing of neocortical inputs (Chung et al., 2021; Dvorak et al., 2018; Dvorak et al., 2021; Fernandez-Ruiz et al., 2021). If coordination between the cortex and hippocampus at specific oscillation frequencies is required for hippocampal signal inhibition or signal transmission within and between hippocampal subfields, it should be sensitive to eFSE-induced perturbation of CA1 and DG dendritic physiology.

We hypothesize that eFSE will affect neocortical-hippocampal input pathways and the frequencies at which dendritic currents oscillate in and out of the membrane. These alterations will in turn alter the ability of hippocampal dendrites to correctly receive cortical inputs at specific subcompartments and transmit their integrated signals along the somatodendritic axes to the cell layer. To this end, we used high-density, laminar electrodes that allow for the simultaneous electrophysiological interrogation of CA1 and DG dendritic compartments corresponding to specific neocortical synaptic inputs (Chung et al., 2021; Dvorak et al., 2021; Fernandez-Ruiz et al., 2021). We made these electrophysiological measurements using induced steady-state slow theta oscillations under urethane (Brankack et al., 1993; Dickson et al., 1994; Hanada et al., 1999; Holscher et al., 1997; Klausberger et al., 2003; Lockmann et al., 2016; Vanderwolf, 1988). Recordings during urethane anesthesia were cost-effective and provided a controlled setting for data analysis where we could control the induction of slow theta oscillations with low variance in power and frequency. In contrast, recording during the active avoidance task presents challenges in accounting for group movement and speed differences that are known to affect our operational measures of theta and gamma oscillations due to the strong correlation of these oscillations to movement speed and cognitive demand (Ahmed & Mehta, 2012; Barry et al., 2020; Mouchati et al., 2020; Zheng et al., 2015).

eFSE resulted in decoupling of slow theta and gamma oscillations along the CA1 and DG somatodendritic axes, leading to shifts in the dominant frequencies where entorhinal-hippocampal synaptic inputs integrate at hippocampal dendritic compartments. It also led to the altered phase coherence of theta and gamma oscillations between cortical input pathways and the CA1 and DG cell layers, resulting in a significant loss of timing or the increased penetration of high-frequency oscillations. The hyperactivity of entorhinal-DG synaptic inputs likely underlies these measures and correlates with the eFSE effects on DG dendritic overgrowth (Patterson et al., 2017). We propose that these alterations in cortico-hippocampal synaptic pathway coordination collectively interfere with the ability of hippocampal dendrites to interpret and propagate inputs from different cortical layers. If frequency-specific syntax at each dendritic compartment is necessary for spatial learning and memory (Fernandez-Ruiz et al., 2021), its loss could be a mechanism for the temporal discoordination of hippocampal neurons and corresponding eFSE cognitive comorbidities (Barry et al., 2016). These results have implications for neurodevelopmental pathologies where the structure and function of neural circuits underpinning cognition are a concern.

## 2 | METHODS

### 2.1 | Animals and experimental overview

All procedures were approved by the University of Vermont and University of California-Irvine animal care and use committees and conducted in accordance with guidelines from the National Institutes of Health. Behavioral and electrophysiological experiments were conducted on 12 adult male Sprague-Dawley rats (CTL=6,eFSE=6). Rats were individually housed on a regular 12-h light/dark cycle with food and water ad libitum. These rats were products of dams raised in quiet, temperature and diurnal-cycle-regulated environments. Pups underwent eFSE induction on postnatal day (P) 10 or P11 and weaned on P22. Spatial cognition was tested between p50 and p60 using the active avoidance task. Rats underwent a terminal, acute electrophysiological assessment under urethane anesthesia from age P70–95. Prior work has shown that female rats do not exhibit severe cognition deficits following early-life seizures (Kloc et al., 2022; Niedecker et al., 2021), therefore only male rats were used in this study. One CTL animal died during the electrophysiological recording process (CTL=5).

### 2.2 | eFSE induction

eFSE was induced at age P10 or 11 as previously described (Barry et al., 2016; Dube et al., 2009; Patterson et al., 2017). Warm air was continuously delivered from a hairdryer above the pups resulting in elevation of core and brain temperatures. The onset of seizure behaviors was observed and recorded. Body temperature was maintained at a fever range (38.5–39.5°C) by regulation of the stream of warm air and measuring core temperature every 2 min. Hyperthermia was maintained for a total time of 60 min. As rat behavior during FS is consistent with the Racine scale, Racine stages were used to characterize seizure intensity and duration. This included stages of (1) Mouth and facial movement; (2) Head nodding; (3) Forelimb clonus; (4) Rearing with forelimb clonus; and (5) Rearing and falling with forelimb clonus. After an initial period of multiple distinct seizures, rats were in eFSE when they did not recover behaviorally between seizures, constituting a continuous “seizure state” that was maintained for a minimum of 30 min. Weight-matched normothermic littermates (CTL) were separated from their dams for the duration of the eFSE induction period and maintained in a separate container on a warming pad (~1 h) to control for maternal separation (Huang et al., 2002). At the end of the procedure, pups were returned to their home cage and dam.

### 2.3 | Active avoidance

**FIGURE 1:**
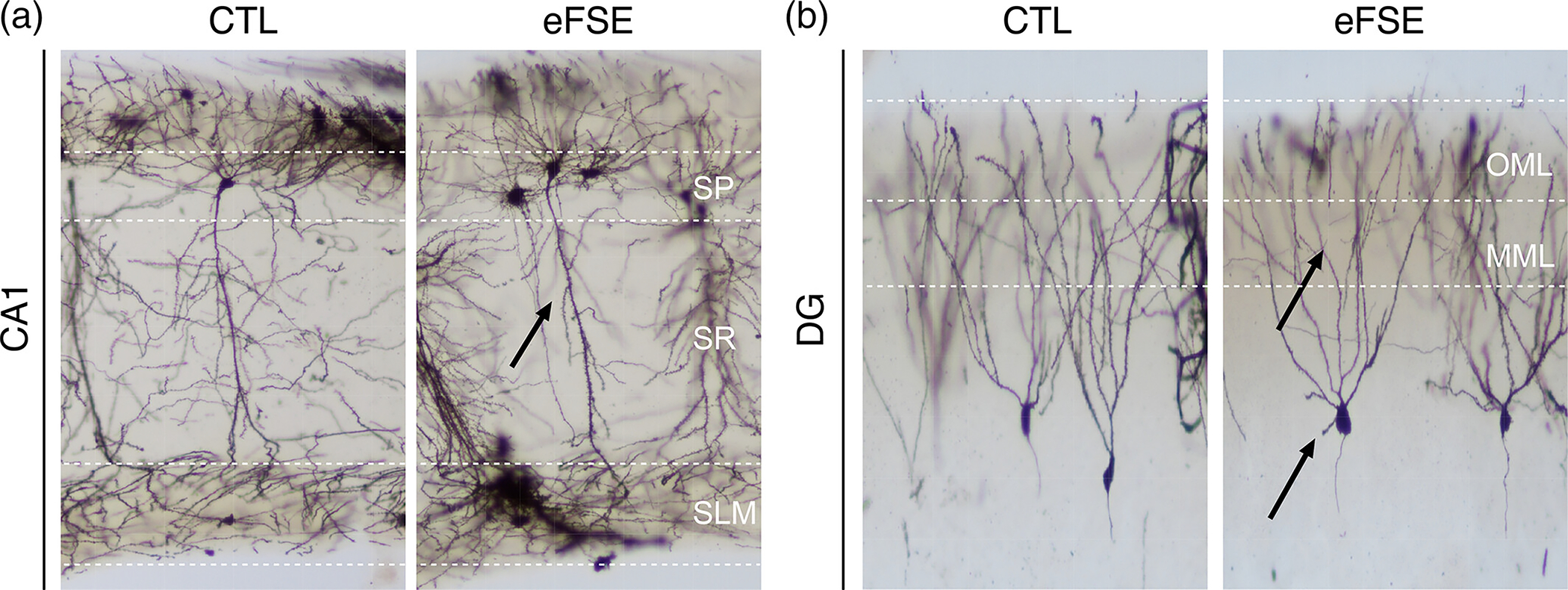
Golgi-stain of hippocampal neurons. Prior work has shown that eFSE causes attenuation of CA1 apical dendrites (a), overgrowth of DG dendritic arbors, and maintenance of immature DG basal dendrites. (b) This raises the question as to whether eFSE fundamentally alters the biophysics of dendritic current oscillations and the frequency of signal coding mechanisms at entorhinal-hippocampal synaptic inputs along both the CA1 and DG somatodendritic axes.

The active avoidance task is hippocampus-dependent (Cimadevilla et al., 2000; Cimadevilla et al., 2001) and was developed to assay spatial learning and memory (Pastalkova et al., 2006). Rats (P50-P60) were anesthetized with isoflurane and implanted with a stainless-steel clip (Eagle Claw, Colorado USA) in the skin of their upper back, between the forelimbs, 1 day prior to the task. Rats were placed on an arena that constantly rotated at 1 revolution per minute (diameter = 82 cm) containing an unmarked 60° arc where an aversive shock was delivered (0.4 mA). A cable with light emitting diode was attached to the clip to deliver the shock when a zone entry was detected. Rats must learn to continuously avoid the unmarked shock zone (Figure 1a, b) using unique visual cues in the room, located distally outside of the arena. Rats were habituated to the arena for 10 min at the start of the experiment and then completed 16 10-min trials across 2 days (8 trials per day).

### 2.4 | Data analysis

#### Active avoidance:

Behavioral data, including a number of shocks and entrances, was analyzed using Track Analysis software (Bio-Signal Group, Brooklyn, NY).

#### Single unit analysis:

Unit activity was filtered from LFP recordings and clustered using the automated spike-sorting software Kilosort2 extension for MATLAB (Pachitariu et al., 2016). All units were examined using the Python-based visualization software Phy (Rossant et al., 2016) and manually reviewed and combined or cleaned as needed. Extracellular action potentials from CA1 SP were only included in subsequent analysis if they exhibited: (1) An average unit amplitude >50 μV; (2) a total spike count >200 spikes and a firing rate of <9 Hz; (3) An autocorrelogram shape representing a refractory period of >1 ms and did not display characteristics of multiunit activity; (4) A wide waveform shape and peak-trough width consistent with excitatory neurons (Fox & Ranck, 1981; Robbins et al., 2013); and (5) Automated cluster analysis (Kilosort2) with a contamination percentage of <5% indicating statistical independence of each isolated cluster. Narrow waveform spike clusters (peak-trough time of <350 μs) identified in the SP cell layer were designated as “likely inhibitory” and analyzed separately (5 CTL units and 6 eFSE units). Clusters were designated as “excitatory” if they exhibited peak-trough widths of 450–600 μs.

Each cell’s preferred phase of firing relative to local theta was analyzed as previously described (Kloc et al., 2021; Mouchati et al., 2020). LFPs were filtered using a Chebyshev type 2 filter and phase was extracted using a Hilbert transform. Spike times were extracted from Kilosort. The trough of the oscillation cycle was denoted 0°/360° and the peak of each cycle was denoted 180°. For each target region of CA1, Rayleigh’s parametric test for nonuniformity of circular data (Fisher, 1993) was calculated as shown in Equation (1) below where n is the sum of the number of incidences in cases of binned angle data and r is the resultant vector length of the distribution. The phase angle and length of the phase vector (RBAR) for each cell were calculated and plotted. The circular mean of each cell in each group was determined by averaging the angle value of each spike and dividing it by the total spike count

(1) $$RBAR=n^{*}r.$$

Greater RBAR values indicate a higher degree of phase preference. As the Rayleigh test is parametric, it assumes a Von Mises distribution of the data set. If the distribution was uniform (i.e., κ=0) this assumption was violated, the test was not run and the mean direction of the resultant vector was not calculated (Berens, 2009; Fisher, 1993). At the population level, the mean resultant length and direction of the vector are calculated through unit vector addition where N represents the number of cells in the population (Fisher, 1993):

(2) $$C=\sum_{i=1}^{n}\cos\theta i,$$

(3) $$S=\sum_{i=1}^{n}\sin\theta i,$$

(4) $$\text{RBAR}=\sqrt{C^{2}+S^{2}/N}.$$

The mean direction of the population associated with resultant length is defined by:

(5) $$\theta_{1},\ldots \theta_{n}={tan}^{-1}S/C+2\pi .$$

#### 2.4.1 | Spectral properties

Power and frequency band properties were analyzed for tail pinch signal (TPS) at slow theta (1–5 Hz), slow gamma (30–50 Hz), and medium gamma (70–90 Hz) bandwidths using custom MATLAB scripts as described previously (Mouchati et al., 2020) using the MATLAB signal processing toolbox “spectrogram” function (MATLAB v2019a; MathWorks). Power was normalized relative to peak amplitudes for each channel during each recording epoch.

#### 2.4.2 | Coherence and voltage correlation

Coherent oscillatory activity was calculated as a measure of signal phase coordination along the DG or CA1 somatodendritic axes (Brankack et al., 1993; Buzsaki et al., 1986) referenced to the distalmost input region of apical dendrites at the outer molecular layer (OML) or *stratum lacunosum-moleculare* (SLM), respectively. The magnitude-squared coherence estimate was applied to determine coherence across groups as a function of depth and frequency (Kloc et al., 2021). The magnitude-squared coherence is a function of the power spectral densities, $P_{xx}(f)$ and $P_{yy}(f)$ and the cross power spectral density, $P_{xy}(f)$, of x and y. To further evaluate signal coordination on each somatodendritic axis, voltage cross-correlation in both groups was analyzed as a function of depth using custom code (Adhikari et al., 2010). LFPs were bandpass filtered and instantaneous amplitude was calculated.

#### 2.4.3 | Current source density

The spatial and temporal distribution of local synaptic currents was assessed by current-source density analysis (CSD) (Brankack et al., 1993; Nicholson & Freeman, 1975; Rappelsberger et al., 1981). LFPs were filtered to 1–8 Hz to encompass slow theta oscillations corresponding with the ~3 Hz tail pinch response. CSD was estimated as the second spatial derivative along every second channel of the 64-channel laminar silicon probe (40 μm increments, 32 channels total). An 8 s epoch within 30 s of the tail pinch was selected for CSD analysis in each animal from each group. As the slow theta frequency could be 0.25–0.5 Hz faster than the average in the first 2 s following tail pinch, we avoided including these epochs in the CSD analysis and selected 8-s epochs toward the middle of tail pinch response. The entire tail-pinch period was processed in all other analyses. The current sinks and sources were then isolated across the 8-s epoch and then averaged (Figure S1). GEE analyses tested for a Group (CTL, eFSE) × Depth interaction for mean sink or source. A single channel near the OML of the DG in the eFSE group was used as a statistical comparator.

#### 2.4.4 | Comodulation index

Cross-frequency coupling (CFC) of phase-amplitude was quantified via modulation index (MI) (Tort et al., 2008; Tort et al., 2010) and applied to unfiltered LFP’s on each channel of the laminar silicon probe. This analysis examines the modulation of the amplitude of high-frequency oscillations by the phase of low-frequency oscillations as an adaptation of the Kullback–Leibler distance function and calculates how much an empirical amplitude distribution-like function over phase bins deviates from the uniform distribution. High-frequency amplitude by low-frequency phase CFC occurs in the hippocampus, where theta phase modulates amplitude of 30–100 Hz gamma oscillations (Bragin, Jando, Nadasdy, Hetke, et al., 1995). In our study, we wished to know if there were group differences in how slow oscillation phase modulated the amplitude of gamma oscillations during the tail-pinch response as a function of depth. The phase-frequency vector in the current study was defined as 1–26 Hz in 0.5 Hz increments while the amplitude-frequency vector was defined as 20–200 Hz in 5 Hz increments. The CFC matrix for the MI was then averaged across the phase-frequency axis at 2.25–5.25 Hz, in correspondence with the predominately 3 Hz oscillation generated by the tail-pinch response. The MI and comodulograms were generated using MATLAB (R2021B) and plotted on a relative jet color scale.

#### 2.4.5 | Statistics

Experimenters were blind to treatment group throughout all experiments and most data analysis. The blind was broken after active avoidance analysis and most electrophysiological analyses were complete. All data described are presented as mean ± standard error (SE). For all statistical tests, p<.05 was considered significant. For active avoidance behavior, the primary outcome measure is the number of shocks and entrances. Generalized linear models (GLM; IBM SPSS v26.0, Armonk NY) or generalized estimating equations (GEE) (Ziegler et al., 1998) were used where noted to generate Wald values to determine significant differences within and between groups. GLM and GEE were used to test for main group effect, main frequency effect, and group-bydepth interactions. These analyses provide a detailed description of what signals/properties were significantly different between CTL and eFSE as a function of recording location. Models were adjusted for repeated measures according to the distribution of each analyzed variable, that is, tweedie probability distribution (including the purely continuous normal, gamma, and inverse Gaussian distributions) with log link models were used for nonparametrically distributed data. Poisson log-linear distribution was used for count data in behavioral analyses. Goodness of fit was determined using the corrected quasi-likelihood under independence model criterion.

For CFC analysis, samples of the mean MI in this phase-frequency band were then taken from channels defined by the CSD and stereotaxic coordinates (Paxinos & Watson, 2014) to be within each region of interest on the somatodendritic axis of CA1 (SP, SR, and SLM) or the DG (OML, medial molecular layer [MML], and dentate granule cell layer [DGC]). GEE was then used to analyze for Group (CTL vs. eFSE) × Regional (SP, SR, and SLM, OML, MML, DGC) interactions for MI at 30–60 Hz, 70–100 Hz and 120–200 Hz where SP in rats with eFSE was used as a statistical comparator. Finally, an additional series of analyses were done in each region to examine for potential Group (CTL vs. eFSE) interactions across amplitude frequency (20–200 Hz).

For phase preference analysis, circular statistics for phase preference were evaluated using Rayleigh’s test for non-uniformity of nonlinear data (Fisher, 1995) within the Circular Statistics Toolbox in MATLAB (Berens, 2009).

Finally, we used receiver operating characteristics curves (ROC) to analyze the diagnostic ability of a binary classifier (SPSS 27.0; Armonk, NY). The true positive rate or sensitivity is plotted against the false positive rate, calculated as one-specificity, at various threshold settings. We used 5 or fewer shocks in 2 consecutive sessions within the 2 active avoidance training days as a binary state measure of performance. Based on our CSD results, we used mean source signal from one channel approximate to the MML DG layer or the average of 6 channels along the DG somatodendritic axis as putative predictors of active avoidance outcomes. The area under curve (AUC) value is used to determine the ability of the test measure to predict cognitive outcomes. An AUC value at 0.50 is statistically no better than chance, 0.51–0.69 is considered a poor predictor, 0.70–0.89 is considered a good predictor, and 0.90–0.99 is considered an excellent predictor (Carter et al., 2016).

### 2.5 | Electrophysiological recordings and experimental logic

Following assessment of spatial cognition on the active avoidance task, wideband LFPs (1–6000 Hz; sampling rate = 30.3 kHz) were recorded across the depth of the CA1 from adult rats as previously described (Kloc et al., 2021). LFPs were measured using a 64-channel laminar silicon probe (interelectrode distance = 20 μm; H3, Cambridge NeuroTech, Cambridge, UK). Rats were anesthetized with urethane (1.6 mg/kg in sterile saline; Sigma Aldrich, St. Louis, MO) and injected intraperitoneally. Dorsal hippocampus was identified bilaterally at −4 mm AP/2.2–2.5 mm ML relative to Bregma. The probe, attached to a digital multiplexing head-stage, was slowly lowered in 1 mm intervals at ~0.05 mm/s every 10–15 min until the tip of the probe was in the hilar region of the dentate gyrus. Probe depth was initially measured relative to skull surface and brain surface (i.e., when first channels at the tip of the probe were grounded against brain signal and free of 60 Hz noise). Continuously sampled LFP recordings were made via the DigitalLynx SX acquisition system and Cheetah software (v6.4, Neuralynx, Boseman, MT) for a minimum of 1 h to ensure that cell firing activity and EEG quality were stable.

After stereotaxic depth measurements from skull and brain surface, probe position was secondarily determined relative to the CA1 stratum pyramidale (SP). Experiment-to-experiment position variability across animals was limited by determining the location of SP and single unit action potentials, and phase reversal from oscillations in descending anatomical regions of hippocampus. Across rats, the experimenter would lower the probe until channels 8–10 were in the superficial SP layer. As in prior studies, SP location was defined offline as the channel with the maximum ripple power (Dvorak et al., 2021) (Figure S2A, Bii). Comparison of recordings made across groups, either at ripple or theta frequency (Figure S2A–C) did not indicate significant differences in hippocampal size between control and eFSE rats. Both electrophysiological measures and analysis of coronal Golgi-stained hippocampal sections indicate that eFSE does not alter the distance between SP and the Hilus (Figure S3).

A tail pinch was held at the base of the tail for approximately 20 s and triggered steady-state theta oscillations throughout the hippocampus for at least 1 min. Foot or tail pinch is an established method of generating slow theta oscillations under urethane (Brankack et al., 1993; Dickson et al., 1994; Hanada et al., 1999; Holscher et al., 1997; Klausberger et al., 2003; Kramis et al., 1975; Lockmann et al., 2016; Vanderwolf, 1988) in both hippocampus and entorhinal cortex (EC) (Dickson et al., 1994) that temporally organizes excitatory and inhibitory neurons (Klausberger et al., 2003; Klausberger & Somogyi, 2008). Theta induction under urethane anesthesia controls for confounding differences in movement speed between eFSE and control animals that are central to the eFSE cognitive deficits seen in the active avoidance task (Ahmed & Mehta, 2012; Barry et al., 2020; Mouchati et al., 2020; Zheng et al., 2015). Slower hippocampal theta frequencies under urethane anesthesia (Type 2 theta) are distinct from movement-related theta with faster frequencies (Type 1 theta) in that they are acetylcholine dependent (Kramis et al., 1975) and associated with sensory processing during arousal (Montoya & Sainsbury, 1985). However, both theta types are dependent on the EC (Dickson et al., 1994; Montoya & Sainsbury, 1985) and reflect the alternating frequency of current propagation in both directions along the somatodendritic axes of hippocampal dendritic membranes (Brankack et al., 1993; Buzsaki et al., 1986). Each alternating cycle of EC synaptic inputs to the apical dendrites, their propagation to the cell layer, and their return from the cell layer to the apical dendrites following action potentials, represents a fine balance of inhibition and excitation over a theta scale timeframe (i.e., 4 Hz = 250 ms). Both theta types, therefore, allow for biophysical measurements of the temporal throughput organization between entorhinal inputs and hippocampal cells, and their reversal from cell layer to synaptic inputs. We hypothesize that eFSE-induced alterations of hippocampal dendrites (Figure 1) (Patterson et al., 2017), which underlie the generation of theta currents, will have consequences for the biophysics of hippocampal signal coordination.

**FIGURE 2:**
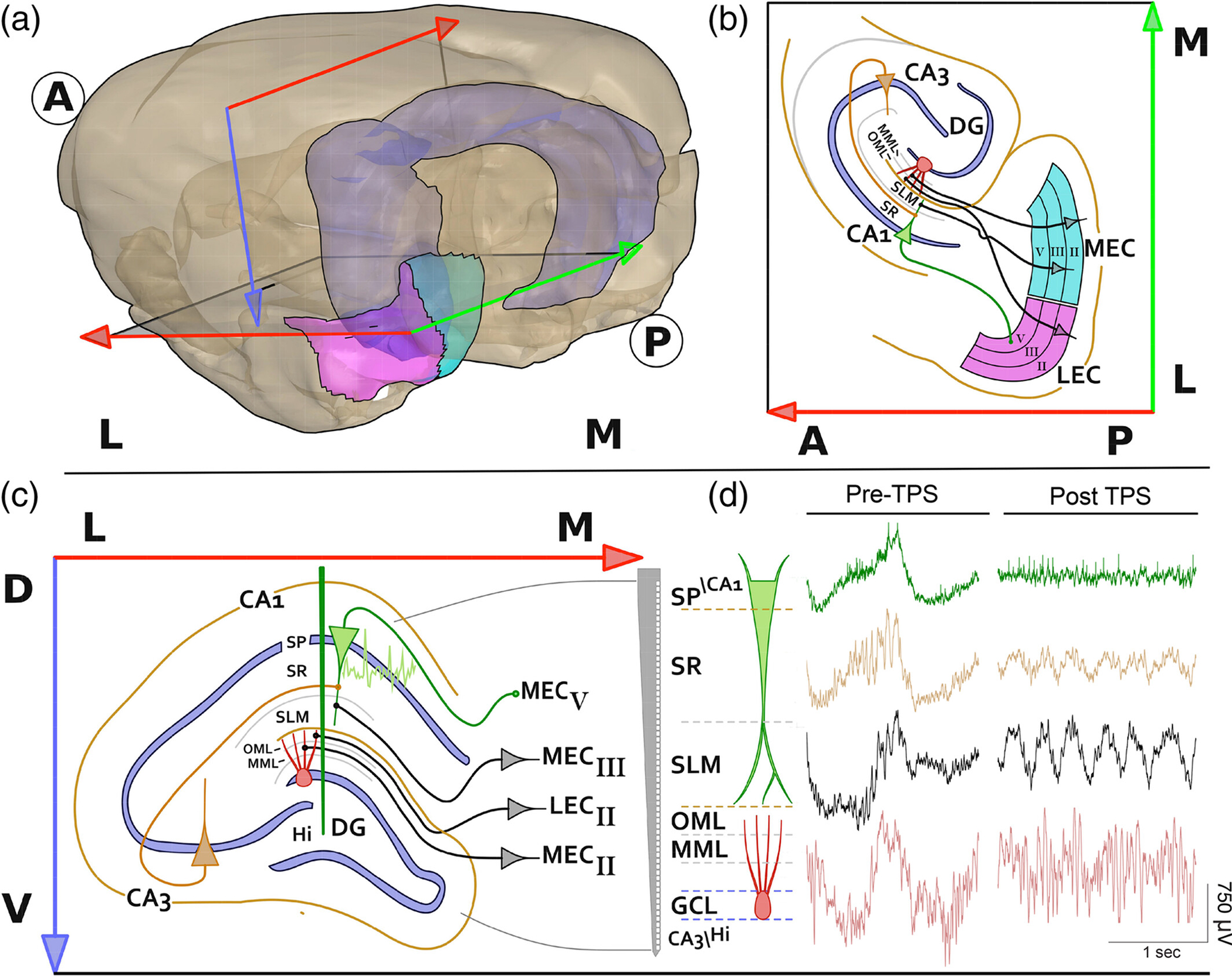
Anatomy of the entorhinal-hippocampal spatial circuit. (a) Dorsal and ventral hippocampus (light blue) relative to the lateral (pink) and medial (aqua green) entorhinal cortex (LEC and MEC) in the coronal plane (red-blue arrows) and the horizontal plane (red-green arrows). Bold A and P denote Anterior and Posterior directions while Bold L and M denote Medial and Lateral directions; (b) horizontal cross section showing direct projections of L3 MEC to the tip of CA1 apical dendrites at *stratum lacunosum-moleculare* (SLM), L2 LEC and MEC projections to the outer molecular layer (OML) and medial molecular layer (MML) of the DG somatodendritic axis respectively. The circuit is complete when CA1/subiculum projects back to L5 of the MEC or LEC; (c) laminar organization of entorhinal-hippocampus projections from L3 MEC to CA1 at SLM, L2 LEC, and MEC to OML and MML of DG and position of a silicon probe (Green line). (d) The silicon probe allows for simultaneous interrogation of *stratum pyramidale* (SP) and DG cell layers as well as axonal projections from LEC and MEC during the transition from spontaneous oscillations to a regular ~3 Hz tail slow theta signal under urethane anesthesia. The high-resolution recording of this slow theta signal post-tail pinch allows for the assay of induced network-level input/output alterations along the somatodendritic axes and putative changes caused by eFSE.

The EC projects to the hippocampus via the trisynaptic loop (L2 MEC/LEC → DG, DG → CA3, CA3 → CA1) and direct projections from L3 MEC to the CA1 apical dendritic tufts in SLM (Hafting et al., 2005; Sargolini et al., 2006; Witter & Moser, 2006) (Figure 2a–c). The circuit is completed with CA1 projections back to L5 of the EC (Ohara et al., 2023). Tail pinch reliably shifted the hippocampal circuit from spontaneous activity to robust 2.5–4 Hz oscillations that were largest in the SLM and OML of DG, corresponding to inputs from the medial EC layer III and lateral EC layer II, respectively (Figure 2c, d). We used oscillations elicited by tail pinch to assay signal coordination and the efficacy of coding mechanisms along the neuronal axis of CA1 and DG neurons. Moreover, as spatial learning and memory requires signal coordination within and between hippocampal subfields and the neocortex (Barry et al., 2016, 2020; Fernandez-Ruiz et al., 2021; Mouchati et al., 2020; Schomburg et al., 2014) we predict that changes to hippocampal signals and the coding mechanisms of entorhinal inputs (Fernandez-Ruiz et al., 2012; Fernandez-Ruiz et al., 2021) could act as biomarkers of spatial learning and memory deficits post eFSE.

To address our hypotheses, we make 4 primary electrophysiological measures as a function of depth along the CA1 and DG axes: (1) Voltage and frequency; (2) CSD; (3) Phase coherence; and (4) Cross frequency coupling between slow theta and gamma oscillations.

## 3 | RESULTS

### 3.1 | Early life eFSE impairs spatial cognition in adulthood

**FIGURE 3:**
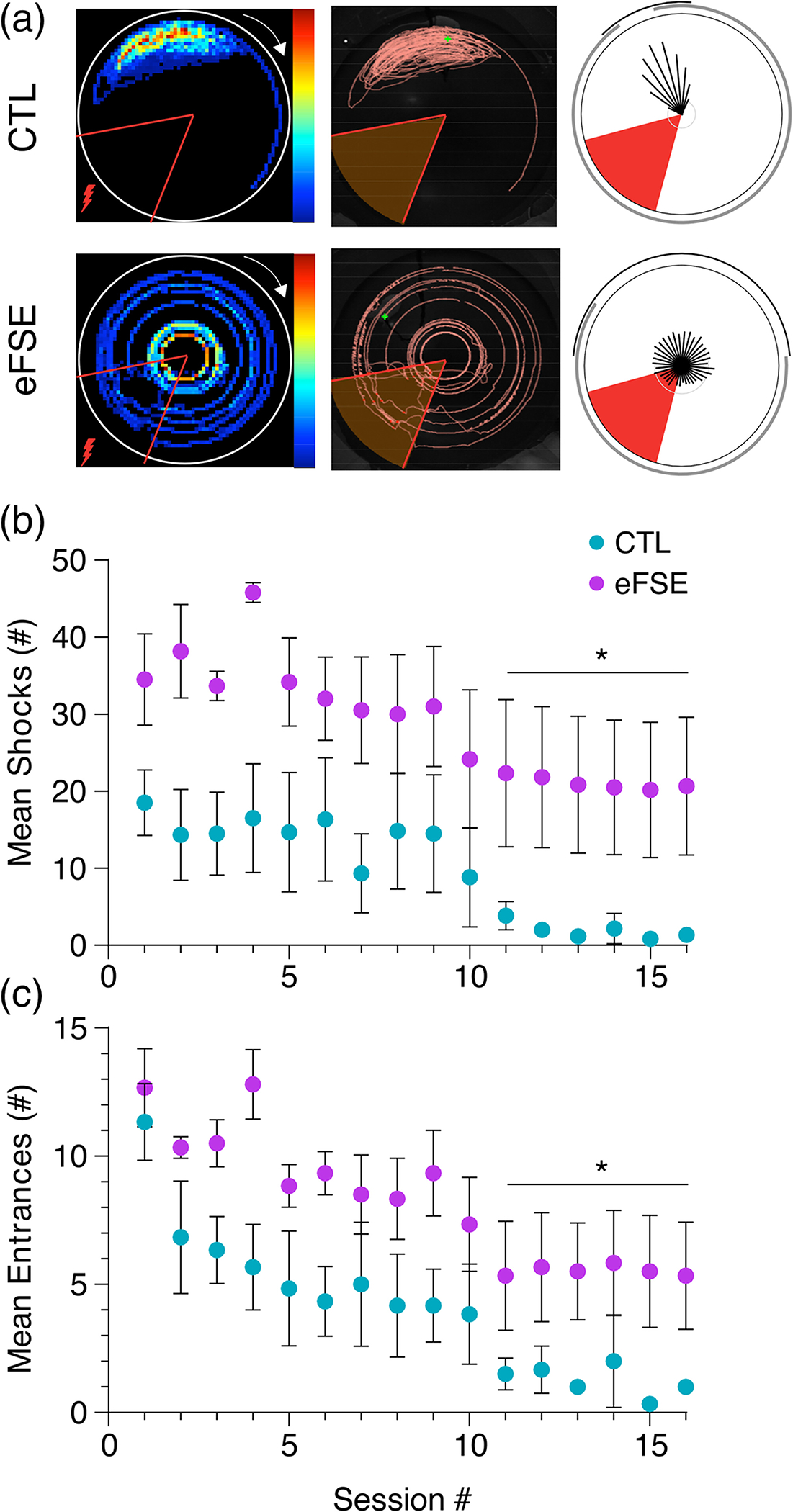
Rats experiencing eFSE at developmental critical periods of entorhinal-hippocampal development exhibit spatial cognitive deficits as adults. (a) Dwell-time (left), path (middle), and circular statistics (right) relative to shock zone (red triangle) for a representative normothermic CTL (top) and eFSE animal (bottom). During 16 training sessions distributed over 2 days, rats with eFSE receive more shocks (b) and make more shock zone entrances (c) than normothermic CTL animals.

On average, the eFSE group (N=6) received significantly more shocks (GEEmaingroupeffectWaldvalue=31.33,p<.0001) (Figure 3a, b) and made significantly more shock zone entrances (GEEmaingroupeffectWaldvalue=15.24,p<.0001) than CTL (N=6) across all sessions (Figure 3c). These data indicate that eFSE early in life results in enduring impairment of active avoidance, apparent in adult rats tested months following eFSE induction. We now use high-density electrophysiology techniques to compare corticohippocampal coding mechanisms and the temporal coordination of hippocampal signals along the CA1 and DG dendritic axes of CTL and eFSE animals. We predict that changes in these signals in eFSE animals will correlate with poor cognitive outcomes on the active avoidance task.

### 3.2 | Tail pinch changes entorhinal-hippocampal circuit state from spontaneous activity to prolonged theta

**FIGURE 4:**
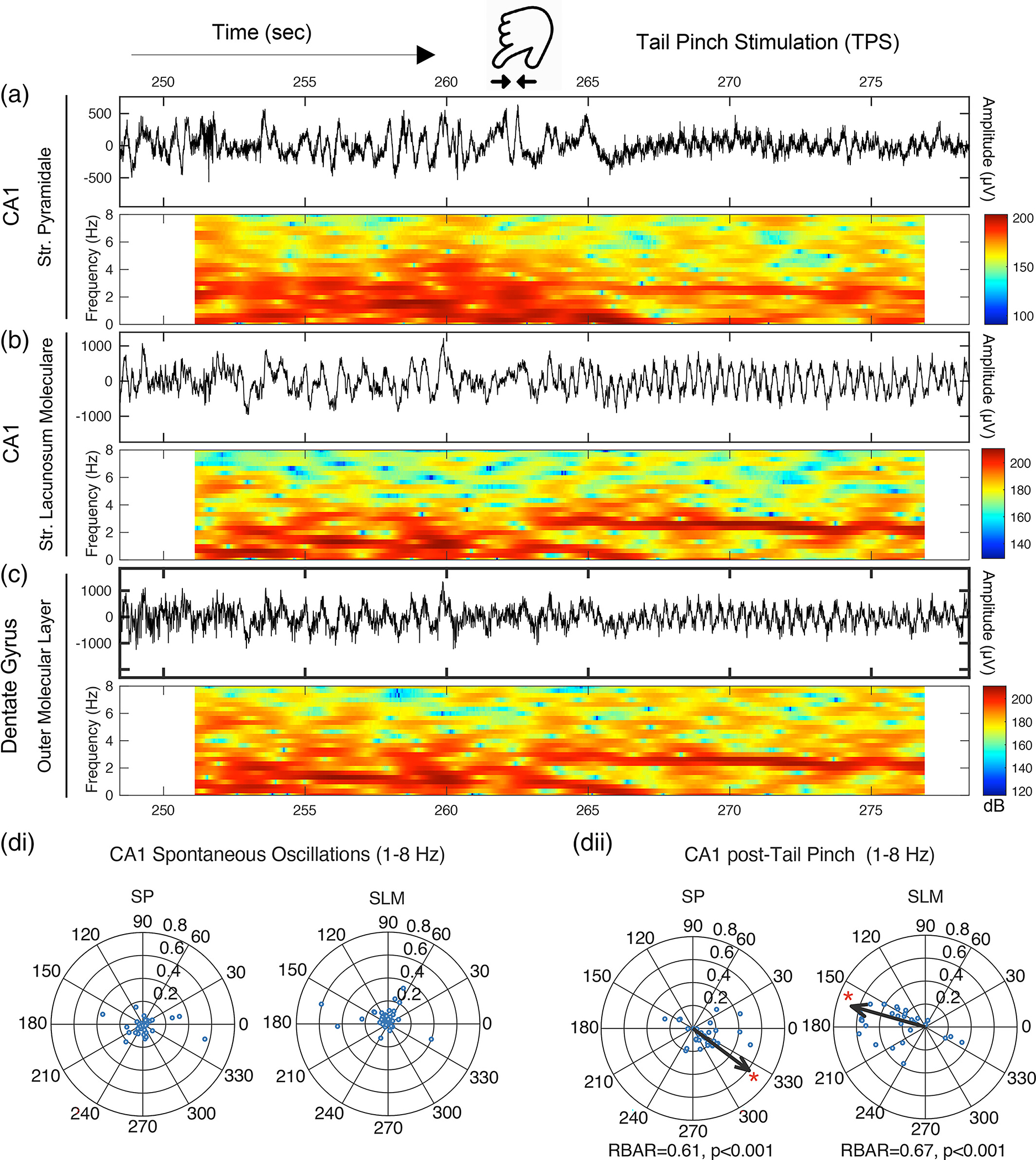
Tail-pinch shifts hippocampal network state from large irregular activity to continuous ~3 Hz slow theta oscillations and increases CA1 theta phase preference. Example of LFP and spectrogram representation of the tail-pinch elicited slow theta oscillations from a CTL rat with recordings from CA1: (a) SP; (b) SLM; and (c) DG OML. The transition from spontaneous large irregular amplitude oscillations to the regular slow theta oscillations of the TPS typically occurs within 2 s. (di-ii) Circular statistics analyzing phase preference of CA1 pyramidal cell action potentials relative to 1–8 HZ LFP oscillations from SP or SLM during spontaneous oscillations and after a tail pinch. Each open circle represents phase preference (*r*) of a single cell. Arrow angle indicates the preferred phase angle while arrow length indicates the resultant phase vector of the cell ensemble. The cell ensemble during spontaneous oscillations, relative to both SP and SLM theta, was statistically uniform. The mean resultant vector was therefore not calculated. Following tail pinch, the circular distribution of CA1 cellular action potentials became phase-locked to 1–8 Hz SLM peaks and local SP troughs, illustrating the ~180° phase lag of L3 MEC inputs at SLM and pyramidal cell action potentials in the SP layer.

While spontaneous oscillations are dominated by low-frequency delta (0.5–1 Hz) (Figure 4a–c), tail pinch shifts the dominant frequency to ~3 Hz in CA1 SP with larger amplitude oscillations in SLM and OML (corresponding to EC inputs at the apical dendrites of CA1 pyramidal neurons and dentate granule cell dendrites). Circular statistics for the 1–8 Hz phase preference of CA1 pyramidal cell action potentials were compared before (Figure 4di) and after (Figure 4dii) the onset of tail pinch, further revealing a network state change with increased temporal organization between the MEC L3 synaptic input pathway and the CA1 somatic output layer at theta frequency. Firing rates of excitatory neurons were not different between groups (CTL=2.98±0.45,N=15;eFSE=3.12±0.6,N=16; Kolmogorov–Smirnov test, p>.05). Likewise, firing rates of putative interneurons were not different between groups (CTL=2.34±1.05,N=5;eFSE=4.61±1.88,N=6; Kolmogorov–Smirnov test, p>.05). Phase preference for clustered excitatory and inhibitory units from CTL and eFSE neurons were not significantly different following tail pinch. Excitatory cells were therefore pooled to further demonstrate the shift in phasic coordination of CA1 action potentials between spontaneous and induced slow theta oscillations.

During spontaneous activity, CA1 neurons (N=37) relative to theta oscillations in SP and SLM exhibited a statistically uniform circular distribution (Figure 4di). The mean resultant vector was, therefore, not calculated. However, following tail pinch (Figure 4dii), the CA1 resultant vector for excitatory neurons (N=31) was significant at $angle=164.2^{\circ }$ relative to SLM ⁡(p<.001), and $angle=323.14^{\circ }$ relative to SP (p<.001). Similarly, the resultant vector for putative inhibitory neurons (N=11) was significant at $angle=118^{\circ }$ relative to SLM (p<.01), and $angle=288^{\circ }$ relative to SP (p<.001) (Figure S4). These results are consistent with the expected phase lag along the CA1 somatodendritic axis (Buzsaki et al., 1986; Mizuseki et al., 2009). Tail pinch stimulation, therefore, induces a short-term continuous network state (also see Figure S2ci) where dendritic currents oscillate across somatodendritic membranes at slow theta frequency.

**FIGURE 5:**
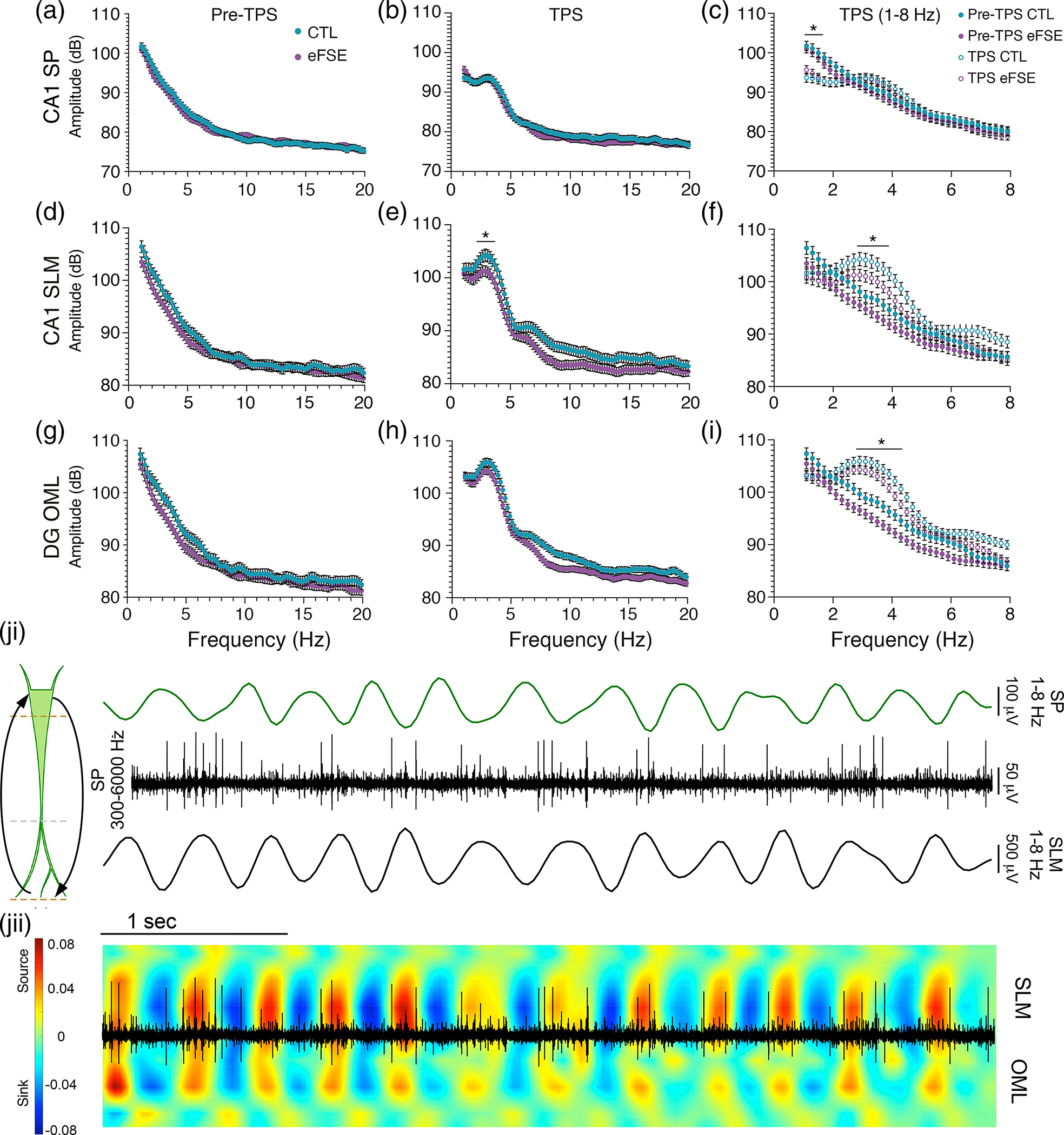
Mean CA1 signal amplitude at 1–20 Hz from eFSE and CTL animals several seconds pre- and post-tail pinch shows dominance of ~3 Hz slow theta signal. (a), (b) CA1 SP, (d), (e) CA1 SLM and G-H) DG OML. (c), (f), (i) Frequency for pre- and post-tail pinch signal (TPS) in each group at 1–8 Hz. (c) SP, (f) SLM, and (i) OML. For both CTL and eFSE groups, tail pinch moves the hippocampal network from spontaneous oscillations to a steady ~3 Hz oscillation state. The mean amplitude of the TPS in CTL was significantly larger than eFSE animals at the ~3 Hz peak in SLM, and OML. Asterisks denote statistical significance; (ji) Sensory stimulation elicits cyclical inputs from entorhinal cortex and outputs from the CA1 cell layer, creating a slow theta dipole along the CA1 apical dendrites. Example LFPs from a CTL rat illustrating an SP signal filtered at 1–8 Hz (Top), the same SP signal filtered at 300–6000 Hz (middle) exhibiting cellular action potentials, and a 1–8 Hz filtered SLM signal (bottom). CA1 cells in the SP layer fire toward the trough of local theta oscillations and the peak of SLM oscillations; (jii) Overlay of 300–6000 Hz filtered SP multiunit activity and the current source density plot of SLM signal filtered at 1–8 Hz. Somatic spikes from CA1 coincide with current sinks and sources underlying the input/output coordination between L3 MEC and CA1 SP.

Power versus frequency analysis highlights the signal property transition between spontaneous oscillations and slow theta (Figure 5). During spontaneous activity, the mean power in both groups is highest at 1 Hz and inversely related to frequency in SP, SLM, and OML (Figure 5a, d, g) with no significant group effect by depth. Post tail pinch, the dominant frequency increases in SP, SLM, and OML (Figure 5b, e, h). When directly compared, the amplitude of 2.5–4 Hz oscillations in both groups is significantly larger than spontaneous oscillations in SP, (GEEmaingroupeffectWaldvalue=7.334,p=.007) SLM (GEEmaingroupeffectWaldvalue=38.97,p<.0001) and OML (GEEmaingroupeffectWaldvalue=10.298,p=.001; Figure 5c, f, i). Comparing the eFSE (N=6) and control (N=5) groups across the bandwidth revealed that eFSE slow theta power was marginally weaker than CTL in SP (GEEmaingroupeffectWaldvalue=4.052,p=.044), and significantly weaker than CTL at SLM (GEEWaldvalue=104.55,p<.0001), and OML (GEEmaingroupeffectWaldvalue=11.704,p=.001).

During tail pinch, CA1 neuronal action potentials are phase-locked relative to the SP trough and SLM peak, corresponding with the alternating sink-and-source motif over time (Figure 5j). This cyclical signal input into the apical dendritic tufts, propagation to the soma, and subsequent reversal creates a dipole along the CA1 apical dendrites. These results indicate that during both spontaneous oscillations and tail pinch elicitation of slow theta there is an eFSE trend toward decreased signal power in CA1 and DG, which is more pronounced in CA1 at the ~3 Hz peak. It is unlikely that this difference is due to changes in electrode impedance over time as recordings were staggered between eFSE and CTL animals. The more parsimonious explanation for these differences is our previous finding of attenuated apical dendrites in CA1 pyramidal cells (Patterson et al., 2017). As current flow in the dendrites is the primary source of hippocampal theta (Johnston et al., 2001), reduced arborization of CA1 dendritic arbors could correlate with a trend toward reduced CA1 theta power in eFSE animals. Alterations of the dendritic arbor could have significant effects on projections of cortical axons at specific dendritic compartments, their essential roles in regulating the spread of unitary LFPs, the generation of transmembrane currents and the frequency coding mechanisms of EC-HC pathway throughput (Fernandez-Ruiz et al., 2021; Sinha & Narayanan, 2022; Telenczuk et al., 2020). As a function of recording depth in each group, we, therefore, measure the CSD, properties of voltage and frequency, coherence, and CFC between theta and gamma oscillations.

### 3.3 | eFSE increases CSD along the DG somatodendritic axes and predicts severity of cognitive deficit

**FIGURE 6:**
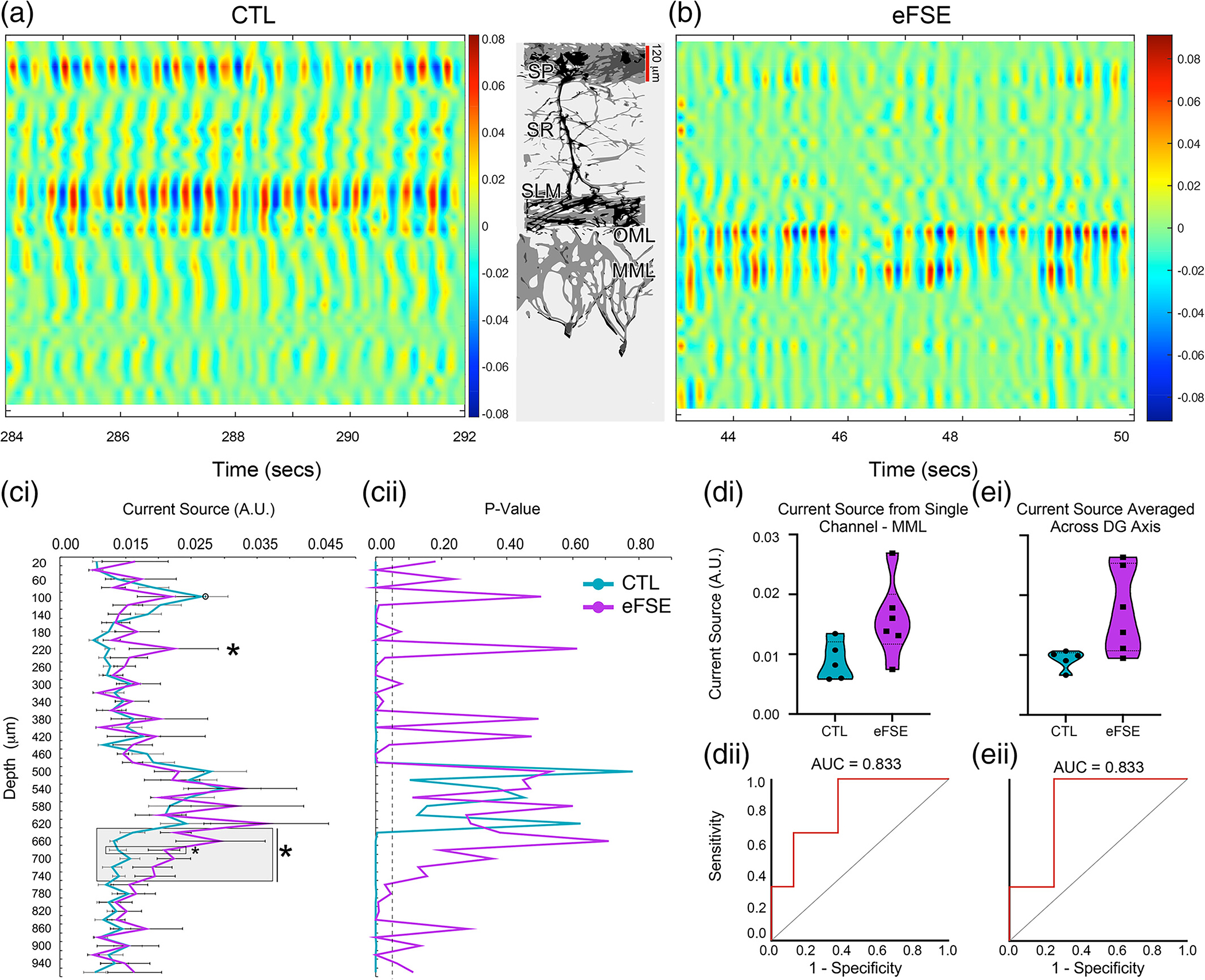
Current source density (CSD) analysis post-tail pinch for CTL and eFSE rats along the CA1 and DG somatodendritic axes and mapping of entorhinal-hippocampal inputs. (a), (b) Example CSD plots from the 1–8 Hz filtered LFPs recorded from a CTL and eFSE rat. Sinks in blue and sources in red represent the theta dipole between the CA1 cell layer and SLM, resulting from L3 MEC projections to SLM and simultaneous projections of L2 LEC to the DG OML. In comparison to CTL, eFSE sinks and sources along the SP-SLM dipole appear weaker while sinks and sources at the DG OML and MML appear larger. (ci-ii) Mean current source density across 8 s post-tail pinch in eFSE and CTL groups. In comparison to CTL, eFSE current sources are significantly larger at SR and along the DG somatodendritic axis. For GEE, open circle at CTL SP denotes the comparator channel. Asterisks denote statistically significant differences between groups. (di)–(ei) Violin plots for the channel corresponding to MML and several channels averaged along the DG axis (gray box ci) illustrate a significantly higher current source across eFSE in comparison to CTL. (dii)–(eii) ROC curve analysis indicates that higher current source levels in (di) and (ei) are predictive of worse cognitive outcomes in eFSE animals on the active avoidance task.

Tail pinch under urethane anesthesia changes the state of the entorhinal-hippocampal circuit, causing a transition from spontaneous activity to temporally organization of activity from entorhinal synaptic inputs at the tips of CA1 and DG apical dendrites. After establishing the efficacy of tail pinch-induced slow theta on modulating hippocampal throughput, we sought to visualize the field-scale organization of EC-HC synaptic inputs via current source density (CSD) analysis. In CTL animals, sink and source signal peaks at 1–8 Hz were found in SP and SLM, corresponding to MEC L3 inputs at SLM and the CA1 theta dipole (Brankack et al., 1993; Buzsaki et al., 1986) with the cell layer (Figure 6a), and OML corresponding to LEC L2 inputs to the outer dendritic arbors of DGC neurons. While the spatial organization as a function of depth was similar in eFSE and CTL rats, eFSE rats exhibit increased sinks and sources along the DG granule cell somatodendritic axis, particularly at MEC L2 inputs in the MML (Figure 6b).

Using current source in the CTL pyramidal cell layer as a statistical comparator, a significant Group × Depth interaction was found for mean CSD ($Wald\;value=4.90\times 10^{13},\;p<.0001$). As suggested by Figure 6a, b, mean eFSE current sources were significantly larger than CTL along the CA1 somatodendritc axis at SR and along the DG somatodendritic axis between the MML and granule cell layers (Figure 6 ci, cii). The current source of the CTL SP layer was equal to that of the eFSE animals and there were no group differences relative to SLM and OML regions. However, group differences were found at SR and along the DG somatodendritic axis between the MML and granule cell layers, where CSD remained significantly higher in eFSE animals (Table S1). A direct group comparison at SR revealed marginal group differences (Waldvalue=4.17,p=.041), while comparisons at MML revealed more robust group differences (Waldvalue=7.67,p=.006). This result suggests that, in eFSE animals, CA1 dendritic currents corresponding to intrahippocampal CA3 inputs and DG dendritic currents corresponding to MEC L2 inputs were significantly higher than currents in the corresponding regions of CTL animals. The increased CSD along the DG somatodendritic axis of eFSE animals may correspond to previous morphological descriptions of hyperarborization in DG granule cell dendrites (Patterson et al., 2017).

Not only do the increased currents in the DG suggest altered biophysics in eFSE hippocampal circuitry, but higher DG CSD levels were also predictive of worse performance on the active avoidance task. ROC analysis for the channel corresponding to the DG MML (Figure 6di, dii) or the average of several channels along the DG somatodendritic axis (Figure 6ei, eii) were significant predictors of whether animals were able to get fewer than five shocks in two consecutive sessions (AUC=0.833). This suggests that increased activity at the MML, and along the DG somatodendritic axis, increases the probability of negative cognitive outcomes post eFSE.

### 3.4 | eFSE alters signal properties and coordination in CA1 and dentate gyrus

**FIGURE 7:**
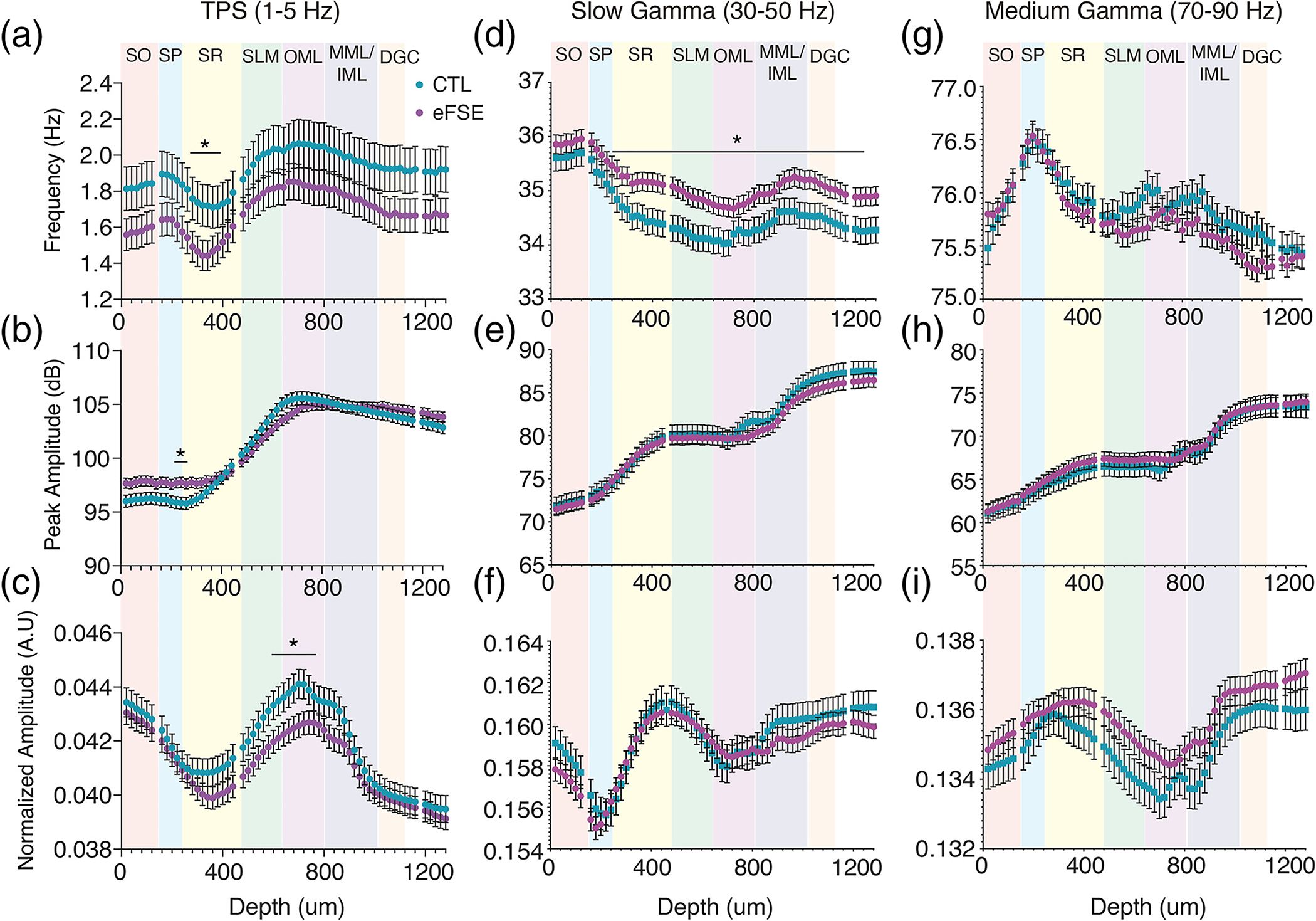
CTL and eFSE mean spectral properties as a function of depth shows specific alterations of hippocampal oscillations. Frequency, peak amplitude, and normalized amplitude of: (a)–(c) LFPs filtered at slow theta (1–5 Hz); (d)–(f) slow gamma (30–50 Hz); and (g)–(i) medium gamma (70–90 Hz). Slow theta peak and normalized power were similar between CTL and eFSE rats. In eFSE rats, theta frequencies were slower at 1–5 Hz and faster at 30–50 Hz oscillations by depth. Normalized 1–5 Hz LFP amplitude was lower in eFSE animals than CTL between SLM and OML, consistent with Figure 6. Slow oscillations are largest in the SLM-OML region while gamma oscillations are largest in SR and the MML-DGC regions.

Analysis of spectral properties exclusively during tail pinch in CTL and eFSE noted specific differences in oscillatory activity in CA1 and DG. Compared with CTL, mean slow theta frequency (1–5 Hz) was only significantly lower in eFSE in the SR (Table S2) but not in SLM or dentate OML (Figure 7a). Peak slow theta power only differed significantly between groups at depths corresponding to regions of transition between SP and SR (Figure 7b, Table S3). Normalized eFSE theta power was significantly lower in SLM and OML, further suggesting EC-HC synaptic activation is weaker during the tail pinch (Table S4).

Slow gamma (30–50 Hz) frequency was significantly higher in eFSE than CTL from SR to the DGC (Figure 7d; Table S5), yet there were no significant group differences in slow gamma power or medium gamma (70–90 Hz) frequency or power. Therefore, apart from slow oscillation power differences at SLM and slow gamma frequencies as a function of depth, baseline signal properties of oscillations generated during tail pinch are largely intact post eFSE.

**FIGURE 8:**
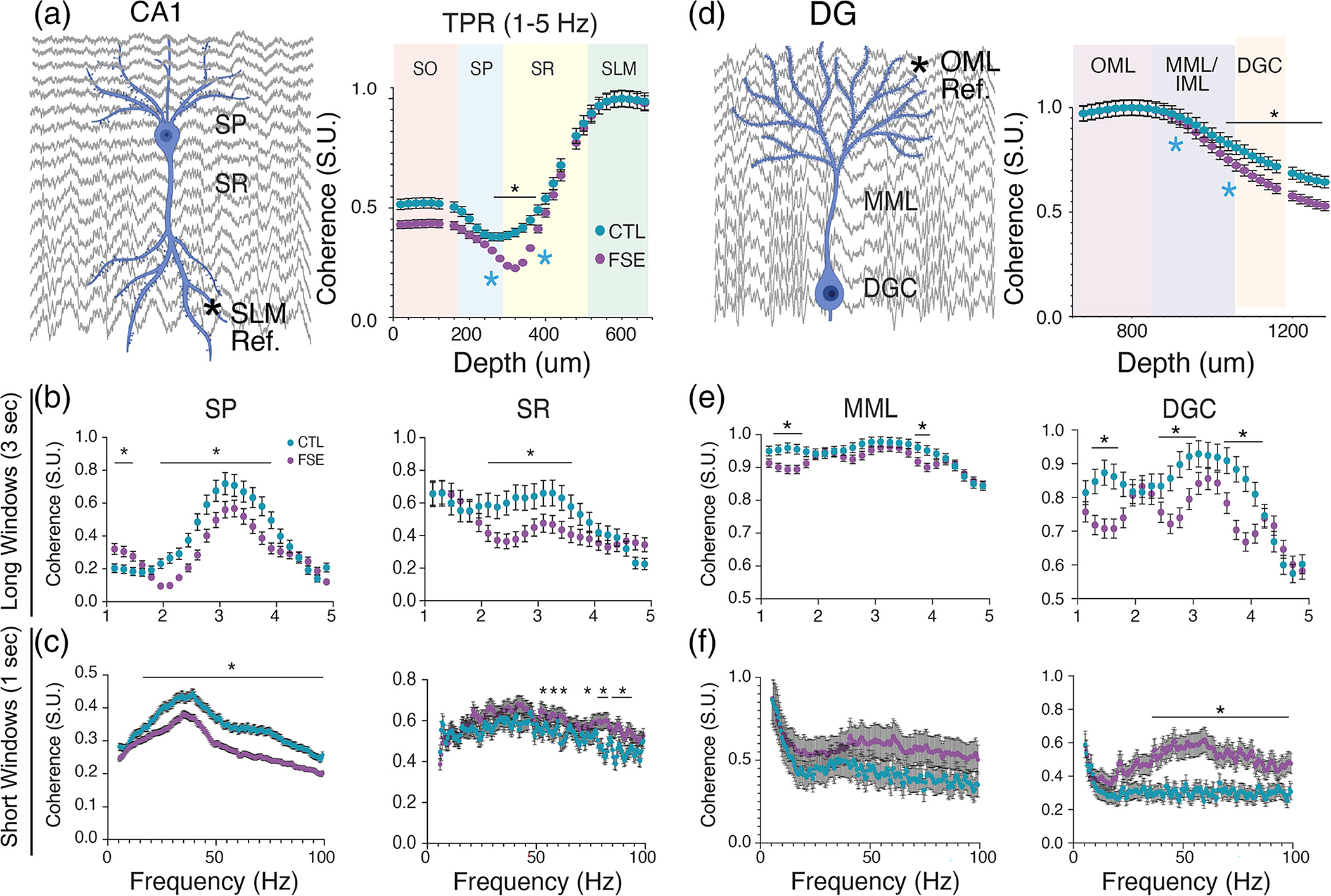
Coordination of tail pinch signal in CA1 and DG is decreased in eFSE relative to CTL. (a) Mean group coherence along the CA1 somatodendritic axis of LFPs filtered at 1–5 Hz, referenced to SLM (left) and analyzed as a function of depth (right). Coherence decreases toward the soma for both groups but is significantly lower in animals with eFSE than CTL between SR and SP layers. (b) Mean coherence by frequency analysis for sampled regions from the SP and SR layers (blue asterisks in right A) using long time windows (3 s). For both SP-SLM and SR-SLM, the 3 Hz slow theta coherence peak is significantly lower in eFSE than CTL, indicative of attenuated phase coordination along the CA1 axis. (c) Coherence by frequency analysis with short time windows (1 s) was used to analyze gamma coherence as a function of depth. In SP-SLM, gamma oscillation coherence was significantly lower in eFSE than CTL rats at signals >40 Hz. (d) Coherence along the DG somatodendritic axis of LFPs filtered at 1–5 Hz, referenced to OML (left), and analyzed as a function of depth (right) for CTL and eFSE rats. Relative to CTL rats, eFSE coherence at 1–5 Hz is significantly decreased toward the granule cell layer. (e) Mean coherence by frequency analysis for sampled regions from the MML and DGC layers (blue asterisks in right D) using long time windows (3 s). While 1–5 Hz MML-OML coherence during TPS is similar in both eFSE and CTL rats, DGC-OML coherence in eFSE rats tends to be lower with narrower peaks than CTL. (f) Coherence by frequency analysis with short time windows (1 s) was used to analyze gamma coherence as a function of depth. While MML-OML gamma oscillation coherence was similar for eFSE and CTL rats, DGC-OML coherence was significantly higher in eFSE than CTL rats across slow and medium gamma bandwidths.

Phase coherence can serve as a measure of signal coordination efficacy within and across brain regions (Laurent et al., 2015; Niedecker et al., 2021). To this end, we examine phase coherence of oscillations in CA1 and DG to determine whether the phase relationship throughout the hippocampus is changed, reflecting physiological differences in the integration and propagation of signals underlying large-scale synaptic inputs. In CA1 we found a Group × Depth interaction for coherence (Wald=3277.80,p<.0001) where all channels were referenced to SLM (Figure 8a; Table S6). Within the slow theta bandwidth (1–5 Hz), phase coherence in eFSE was significantly decreased in SP (Maingroupeffect;Wald=30.66,p<.0001) and SR (MaingroupeffectWald=24.41,p<.0001) in comparison to CTL (Figure 8b; Tables S7–S8). Higher frequency eFSE bandwidths during tail pinch were also significantly less coherent in SP (MaingroupeffectWald=465.5,p<.0001), and SR (MaingroupeffectWald=239.8,p<.0001). However, in SR these differences were less robust frequency by frequency (Figure 8c; Tables S9–S10). These results suggest that, in animals with eFSE, oscillations propagate from SLM to SP in a less coordinated and efficient fashion than CTL.

In dentate gyrus, a significant Group × Depth interaction was found (Wald=5275.93,p<.001). Phase coherence referenced to the OML was not altered in the MML but was significantly lower in the DGC (Figure 8d; Table S11). Within the slow theta bandwidth, group differences were found in the DGC (MaingroupeffectWald=54.22,p<.0001) but not MML (MaingroupeffectWald=1.104,p=.293) where peak coherence was in a much narrower frequency range in the eFSE DG cell layer than CTL (Figure 8e; Tables S12–S13). At higher frequency bandwidths (Figure 8f), there was again no significant group difference in MML (MaingroupeffectWald=0.417,p=.519;), while DGC in eFSE animals was significantly more coherent with OML (MaingroupeffectWald=1007.12,p<.0001; Table S14). These results suggest that in comparison to CTL, eFSE granule cell dendrites were poor low-pass filters and allowed propagation of high-frequency oscillations from the OML to the granule cell layer.

Voltage correlation (Adhikari et al., 2010) is a complementary measure of signal coordination efficacy that measures the timing of amplitude epochs during oscillatory signaling. While there were significant group effects relative to phase coherence, there was no statistical main group difference in voltage correlation (Datanotshown;Wald=0.203,p=.652). Therefore, eFSE effects are more likely to effect phase integration and propagation along the DG and CA1 somatodendritic axis than the amplitude envelope. As in other seizure models, coordinated hippocampal signal timing is more of a concern than coordinated signal size (Laurent et al., 2015).

### 3.5 | Altered CA1 and DG theta-gamma coupling post-eFSE

**FIGURE 9:**
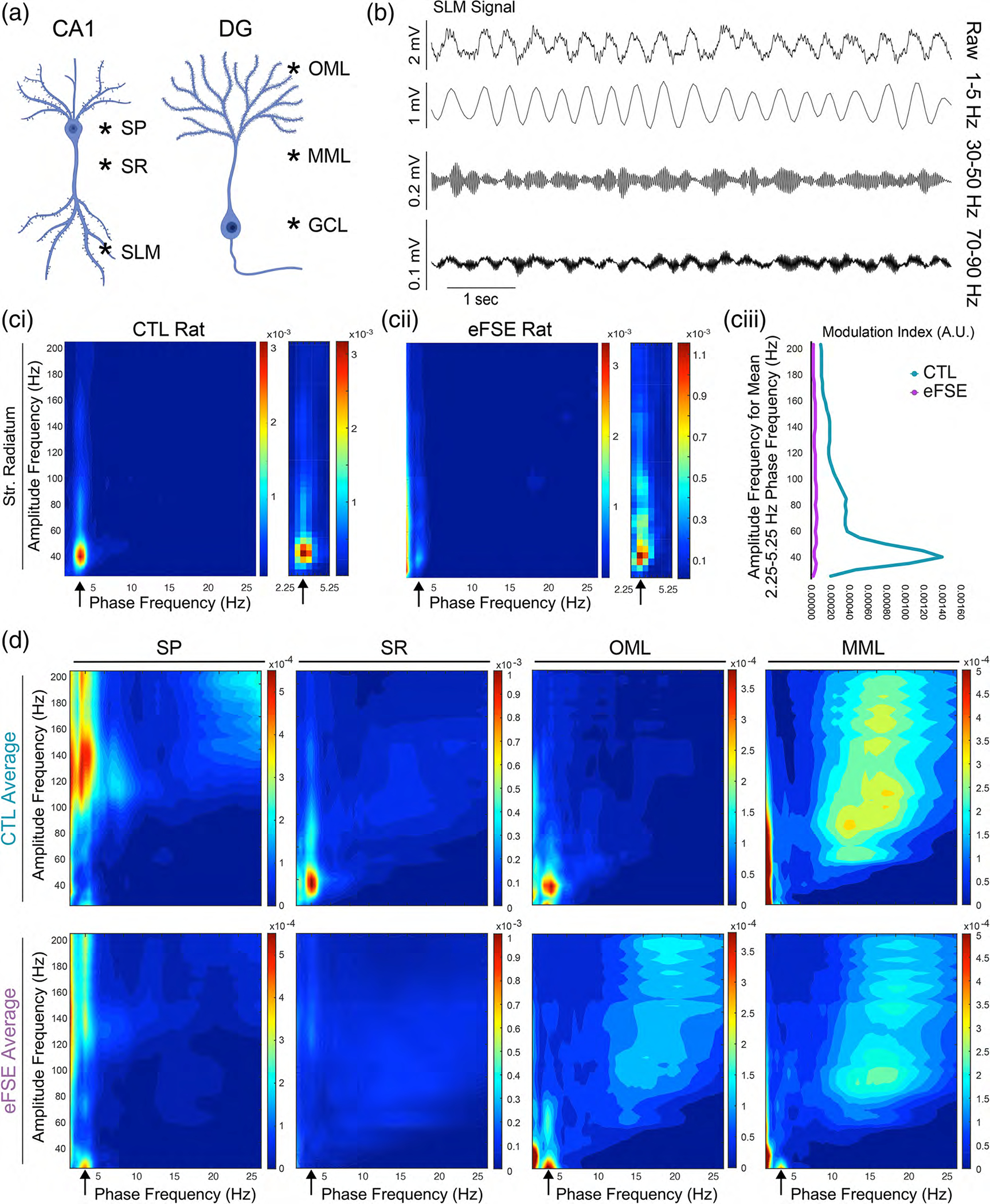
Cross frequency coupling on the CA1 and DG axes. (a) Regions of interest on the CA1 and DG somatodendritic axes sampled from each rat in each group. (b) Illustration of frequency dynamics from an unfiltered LFP in the SLM of a control animal following tail pinch, and the same signal filtered at 1–5 Hz, 30–50 Hz (slow gamma), and 70–90 Hz (medium gamma). (ci) Example comodulogram from a CTL rat at SR illustrates that the phase frequency stemming from the ~3 Hz TPS is strongly coupled to the amplitude-frequency at ~40 Hz. (cii) Although this slow gamma coupling is present in the eFSE example at SR, it is more diffuse and attenuated in comparison to the CTL example. (ciii) Plots of the mean modulation index of the 2.25–5.25 Hz phase frequency against the amplitude frequency indicating that slow theta and slow gamma coupling may be reduced in the eFSE animal. (d) Averaged modulation index of cross-frequency coupling across CTL (top) and eFSE (bottom) animals at SP, SR, OML, and MML. In CA1, eFSE animals exhibit reduced 3 Hz coupling with fast gamma at SP and slow gamma at SR and SLM. In DG, eFSE animals show less differentiation than CTL along the somatodendritic axis at OML and MML, with increased 3 Hz coupling at slower gamma frequencies.

CFC represents coordination within and between neural networks on multiple timescales (Tort et al., 2008), where slow frequencies have been found to modulate the amplitude of fast oscillations. Theta and gamma CFC (Belluscio et al., 2012; Bragin, Jando, Nadasdy, Hetke, et al., 1995) has been proposed to underpin organization of sequential memory and working memory (Lisman, 2005; Tort et al., 2008; Tort et al., 2009) while disruption of CFC is associated with psychiatric impairment (Kirihara et al., 2012), local inhibitory dysfunction and increased risk of seizure (Jansen et al., 2021). In order to further examine the network effects of altered physiology and signal coordination in eFSE animals, we analyzed CFC in both CTL and eFSE rats as a function of depth on the CA1 somatodendritic axis (SP, SR, and SLM; Left Figure 9a) and the DG somatodendritic axis (OML, MML, and DGC; Right Figure 9a). An example of phase and amplitude coupling between slow oscillations and gamma oscillations is shown in Figure 9b through an unfiltered SLM signal and the same signal filtered in theta, slow gamma, and medium gamma bandwidths. Examples of CFC comodulogram plots from a CTL rat along each somatodendritic axis in CA1 and DG are also shown in Figure S5. Individual examples of CFC at SR in a CTL and eFSE rat and the method of extracting MI values from the 2.25–5.25 phase frequency against the 25–200 Hz amplitude frequency are shown in Figure 9ci–cii.

**FIGURE 10:**
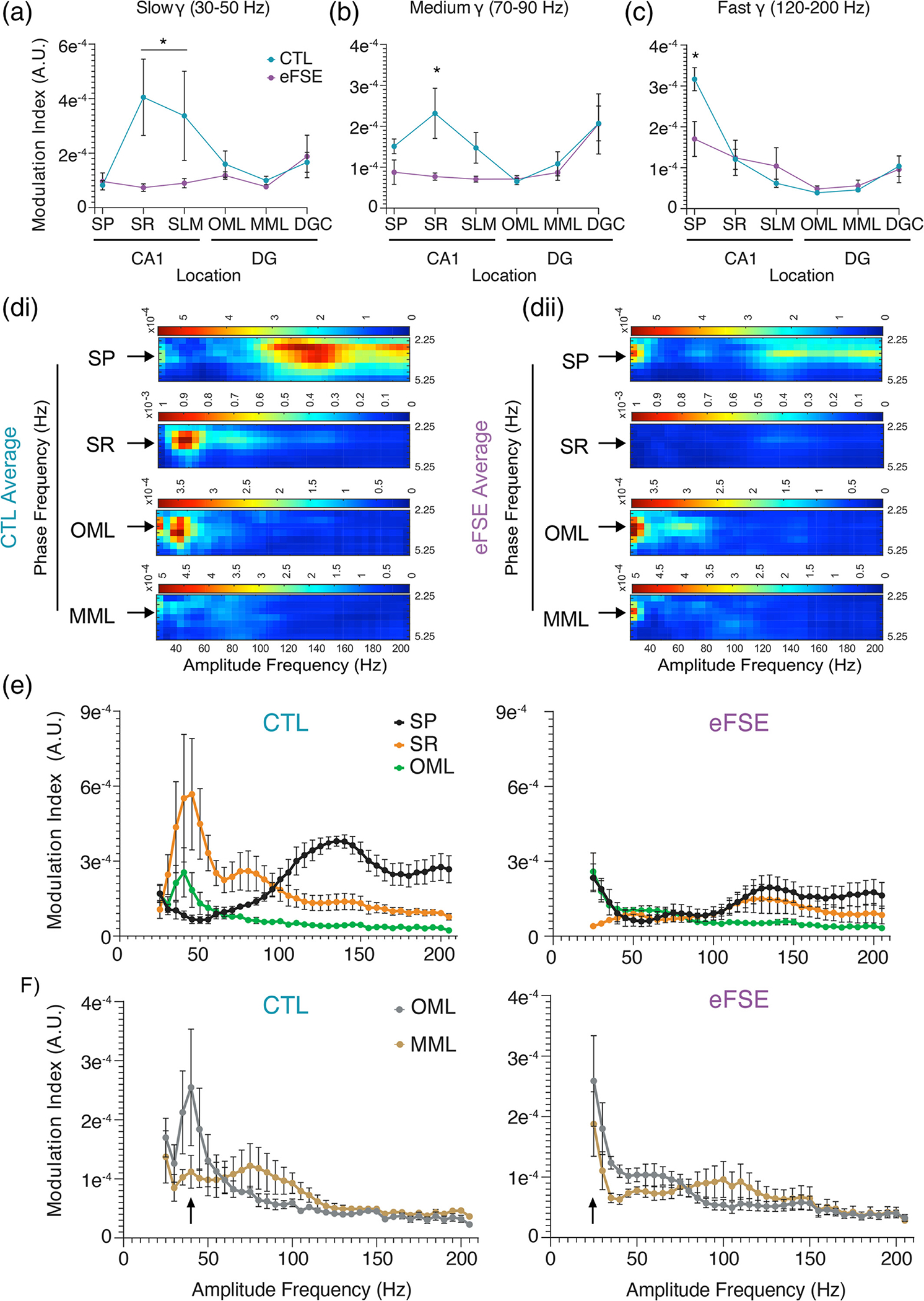
Cross frequency coupling (CFC) in eFSE animals is altered by depth. (a)–(c) Modulation index of CA1 and DG regions of interest for slow gamma, medium gamma, and fast oscillations (120–200 Hz). In CTL rats, LFPs during tail pinch exhibit significant CFC with slow gamma oscillations at SR and fast oscillations at SP. In eFSE animals, CFC of gamma and fast oscillations during TPS is significantly attenuated at SR and SP. (d) From Figure 9d, averaged modulation index for 2.25–5.25 Hz phase frequency in CTL (di) and eFSE animals (dii). As averaging across bandwidths can miss key details about frequency tuning, the modulation index for 2.25–5.25 Hz was analyzed across 25–200 Hz amplitude frequencies via GEE. (e) Rats with eFSE exhibit attenuated CFC across gamma bandwidths at SP, SR, and OML. (f) The CTL rat pattern of differential CFC at OML and MML synapses relative to slow and medium gamma is contrasted by eFSE downward shifts in CFC at the same 25 Hz frequency.

The mean MI for each region and group is shown in Figure 9d. Apart from a lack of medium gamma domination in SLM, the CTL pattern of 3 Hz coupling with fast gamma in SP and a tendency toward 3 Hz and slow gamma coupling along each somatodendritic axis has some overlap with previous work showing laminar CFC patterns while rats performed a spatial alternation task (Buzsaki & Schomburg, 2015). In eFSE rats, 3 Hz-gamma coupling was attenuated along the CA1 axis. While the DG axis in CTL animals shows differential coupling at the OML and MML synaptic input regions, the gamma coupling amplitude frequencies are similar in eFSE animals and shifted to slower frequencies. We therefore analyzed the CFC data in two ways: (1) By averaging MI across slow, medium, and fast gamma bandwidths at each region (Figure 10a–c); and (2) By analyzing MI across 25–200 Hz amplitude frequencies for each region (Figure 10d–f).

A significant Group × Depth interaction was found for mean MI in the slow gamma bandwidth (Waldvalue=12313.15,p<.00001). In the eFSE group, there was no statistical difference between SP and all other regions, denoting an absence of CFC along each axis (Figure 10a). However, in CTL animals there is a statistically significant increase in MI in CA1 SR and SLM (Table S15) indicating peak coupling between 3 Hz and slow gamma at SR. In the medium gamma bandwidth, there was a significant Group × Depth interaction for mean MI in the CTL group (Waldvalue=794.29,p<.00001). In the eFSE group, there was no statistical difference between SP and all other regions, denoting an absence of CFC along each axis (Figure 10b). MI in CTL animals exhibited a statistically significant increase in both CA1 SR and the DGC, with peak coupling between 3 Hz and medium gamma at SR (Table S16). There was also a significant Group × Depth interaction for mean MI in the fast gamma bandwidth (120–200 Hz) in CTL animals (Waldvalue=3106.18,p<.00001). Slow theta coupling with fast gamma oscillations was localized to CA1 SP for both CTL and eFSE animals (Figure 10c). However, the level of coupling in eFSE SP was significantly lower than CTL SP and was only statistically greater than eFSE OML and MML (Table S17). By averaging across gamma bandwidths, we found that in CTL animals the 3 Hz slow theta signal was significantly coupled to gamma along the CA1 somatodendritic axis and fast oscillations in CA1 SP. However, in eFSE animals coupling between 3 Hz and both gamma and fast oscillations was significantly attenuated. As this analysis did not account for putative frequency tuning differences within each bandwidth, we conducted an additional analysis examining putative Group × Amplitude Frequency changes at each region of interest on the CA1 and DG somatodendritic axes (Figure 10di, dii). In correspondence with previous analysis, Group × Amplitude Frequency (25–200 Hz) showed a peak CFC in CTL animals between 3 Hz and ~140 Hz in SP and ~40 Hz in both SR and OML (left Figure 10e). Although slow theta amplitude was significantly reduced in the SLM and OML of rats with eFSE in comparison to CTL, differences were nonsignificant in other locations on the somatodendritic axis and no significant group differences were found in the amplitude of slow gamma. Therefore, despite the presence of slow theta and slow gamma LFPs in rats with eFSE, 3 Hz oscillations at SP, SR, and OML did not couple with local gamma in the same manner as CTL animals (right Figure 10e). For analysis of Group × Frequency interactions, frequencies corresponding to MI peaks in CTL animals were used as statistical comparators in each region. In CTL there were significant Group × Frequency interactions for CFC at SP, SR, and OML (Tables S18–S20) where eFSE CFC was significantly different from CTL peaks in each region of interest; SP CFC peaked at 135 Hz ($Wald\;value=8.17\times 10^{11},\;p<.00001$), SR CFC peaked at 45 Hz ($Wald\;value\;=4.98\times 10^{11},\;p<.00001$), and OML peaked at 40 Hz (Waldvalue=559.57,p<.00001). SP coupling with fast oscillations and SR and OML coupling with slow gamma oscillations were therefore significantly reduced in eFSE animals in comparison to CTL.

In CTL animals, there was differential CFC in the OML and MML, where OML showed more coupling to slow gamma and MML showed more coupling with medium gamma (left Figure 10f). This regional pattern has some commonalities with gamma coupling in freely moving rats performing object or spatial memory tasks (Fernandez-Ruiz et al., 2021). In correspondence with CTL and eFSE CSD differences between OML and MML, there were group differences in slow theta and gamma coupling at these synapses. These group differences in CFC were not detected when averaged across the bandwidth as eFSE coupling at OML and MML remained high but shifted to lower frequencies (right Figure 10f). At OML, there was a significant Group × Frequency interaction (Waldvalue=559.57,p<.00001) where mean CTL CFC peak at 40 Hz was larger than all other points in CTL except adjacent frequencies at 35 and 45 Hz, as well as at every eFSE CFC frequency except 25–30 Hz (Table S21). Similarly, GEE found a significant Group × Frequency interaction at MML ($Wald\;value=2.3\times 10^{9},\;p<.00001$) where the CTL CFC levels at 40 Hz were greater than CTL fast oscillations at 125–200 Hz, and equal across slow and medium gamma ranges at 25–100 Hz (Table S21). CTL CFC levels at 40 Hz were significantly greater than eFSE levels at 155–200 Hz and 40 Hz. Importantly, as in OML, the CTL 40 Hz MI was also equal to 25–30 Hz in the eFSE animals at MML. Therefore, rather than exhibiting differential gamma coupling at OML, eFSE animals exhibit peak coupling at OML and MML synapses that are significantly shifted downward to 25 Hz.

Taken together with CSD measures indicating increased eFSE synaptic activity at MML, OML and MML coupling at the same frequency may impede DGC segregation of MEC L2 and LEC L2 signal projections and the upstream segregation of cell assemblies (Fernandez-Ruiz et al., 2021). Diminished phase-amplitude modulation in CA1 and homogenization of phase-amplitude modulation across the DG could alter the ability of hippocampal dendrites to interpret and integrate neocortical inputs, providing a mechanism for eFSE induced spatial cognitive impairment.

## 4 | DISCUSSION

eFSE is associated with spatial cognitive deficits and changes to the morphology of both CA1 and DG dendrites in the hippocampus (Barry et al., 2016, 2020; Kloc et al., 2022; Patterson et al., 2017). We wanted to know what the functional consequences were to these eFSE-induced physiological changes as coordination between the cortex and both hippocampal subregions has been proposed to be necessary for cognition (Bellmund et al., 2020; Fernandez-Ruiz et al., 2021; Gerlei et al., 2021; Igarashi et al., 2014; Lopez-Madrona et al., 2020; Niedecker et al., 2021; Olafsdottir et al., 2016; Quirk et al., 2021; Roy et al., 2017; Suh et al., 2011). Using high-density silicon probes, our study focused on how eFSE-induced changes to the electrophysiology of specific dendritic compartments in CA1 and DG affected the efficacy of entorhinal-hippocampal synaptic inputs along the somatodendritic axis of each subfield. This approach allowed us to measure how eFSE changed the nature of these synaptic input/somatic output relationships that underscore the temporal coordination of neurons via local dendritic current oscillation frequencies (Barry et al., 2016). We hypothesized that status-induced alterations to CA1 and DG physiology would alter both the post-synaptic coding of neocortical-hippocampal input pathways at specific frequencies and their throughput to somatic cell layers. In agreement with this hypothesis, we discovered a reorganization of synaptic current sinks and sources along the CA1 and DG somatodendritic axes, diminished theta-gamma amplitude-phase coupling at specific entorhinal synaptic inputs, and altered intrahippocampal phase coordination.

To our knowledge, this was the highest density assay of cortico-hippocampal circuit function to date in a developmental pathophysiology model. The 20 μm spacing between laminar electrodes provided access to the somatic cell layers of CA1 pyramidal cells and DG granular cells and the field currents generated between each of their dendritic compartments and axon terminals from the entorhinal cortices. The laminar probe thereby gave us unprecedented access to both CA1 and DG structures simultaneously and allowed for a detailed assessment of how their function could be altered by a prolonged seizure in early development. Notably, our electrophysiological findings align with structural assessments of hippocampal neurons impacted by eFSE. Whereas eFSE does not induce cell loss, CA1 dendritic branching is attenuated, and DG granule cell apical dendrites are hyperarborized (Patterson et al., 2017). The current findings illustrate the consequences of these physiological changes and their alteration of the operational frequencies at specific entorhinal synaptic input pathways. Disrupted communication between the neocortex and hippocampus at these CA1 and DG dendritic compartments, and theta phase incoherence between cortical inputs and the CA1 soma, could serve as a novel mechanism for the temporal discoordination of CA1 place cells relative to local field oscillations and associated deficits in learning and memory.

In CA1, CSD analysis found that synaptic input activation is decreased, while coherence analysis by depth showed that signal propagation efficacy was also decreased. Attenuation of CA1 dendritic branches, particularly at SR, may be sufficient for local gamma coupling impairments shown here and in previous work for theta and gamma voltage correlation between CA1 and CA3 (Barry et al., 2016). These differential effects to hippocampal subfields may decrease intrahippocampal connectivity and the balance of sensorimotor and recall processing needed to support spatial task demands (Barry et al., 2020; Patterson et al., 2017). In DG, CSD analysis also revealed that entorhinal synaptic inputs are hyperactive, particularly at MECDG synapses in the medial compartment of the molecular layer. Remarkably, current source levels in this region had predictive power regarding the severity of eFSE cognitive deficits in the active avoidance task. Further work needs to be done to determine whether eFSE-induced CSD increases at dentate synapses are due to the synaptic hyperactivity caused by dendritic overgrowth (Getz et al., 2022; Luikart et al., 2011; Williams et al., 2015), developmental misguidance of L2 projection terminals targeting dendritic compartments, or general restructuring of axonal projections along the proximodistal axis (Bender et al., 2007; Laurent et al., 2015; Xu et al., 2021). It is also possible that eFSE could affect the inhibitory activity of hilar or molecular layer interneurons projecting to the perforant path (HIPP or MOPP cells) which regulate the OML inputs from LEC2, or the “Total Molecular” cells that are positioned to regulate feedback inhibition of medial perforant path inputs (Andersen, 2007; Ewell & Jones, 2010; Frotscher, 1991; Li et al., 2013; Raza et al., 2017; Soriano & Frotscher, 1993). However, for this hypothesis to be adequately proven, the somatic activity of the inhibitory cells would need to be significantly decreased in comparison to controls. Even if interneuron activity is unchanged by eFSE, the efficacy of dendritic inhibition could still be affected by eFSE-associated dendritic overgrowth (Patterson et al., 2017) as the dendritic targets of many interneurons form a critical substrate for inhibitory microcircuits. This could be tested by enhancing local DG inhibitory neuron activity and measuring the effects of increased inhibition on DG throughput (Mattis et al., 2022). While the mechanism of relative eFSE hyperactivity at OML and MML is yet to be determined, it is clear the governance of the medial and lateral perforant pathway is altered by eFSE. Given the known morphological changes in this model, it is, therefore, possible that dendritic overgrowth could be sufficient for local hyperactivity, rather than excessive inputs from the EC (Getz et al., 2022; Mattis et al., 2022). As it is possible that increased MML synaptic activity could allow more signals to penetrate the hippocampal circuit, thereby partially explaining a clear tendency toward increased theta scale CSD in the SR of eFSE animals, future work should also examine the gating efficacy of the MML in other bandwidths and during dentate spikes (Dvorak et al., 2021). Poor gating of the DG could lead to temporal discoordination between CA1 and CA3, a known phenotype in the eFSE model (Barry et al., 2016, 2020). We also showed that phase coherence between the OML and DGC was significantly higher in eFSE than CTL rats across gamma bandwidths. High-frequency gamma oscillations in eFSE rats were more likely to penetrate from the OML to the DGC in comparison to CTL rats. Therefore, dendritic hyperarborization in DG cells post eFSE (Patterson et al., 2017) makes distal DG dendrites less effective low-pass filters (Ewell & Jones, 2010; Getz et al., 2022; Krueppel et al., 2011; Luikart et al., 2011; Williams et al., 2015). Yet, it is unclear how synaptic field hyperactivity and increased penetration of high-frequency oscillations between the OML and granule cell layer affect overall dentate gyrus function. Changes to the regulation of dendritic synaptic inputs in the perforant pathway could also affect the generation of dentate spikes, or large amplitude, short duration field potentials localized to the DG. Dentate spikes are initiated through entorhinal activation and are thought to cause a synchronized inhibition of granule cells and downstream CA1 and CA3 subfields (Bragin, Jando, Nadasdy, van Landeghem, & Buzsaki, 1995; Penttonen et al., 1997) and may be necessary for the alternation between recall and sensory information processes underpinning spatial active avoidance (Barry et al., 2020; Chung et al., 2021; Dvorak et al., 2018; Dvorak et al., 2021). This would partially explain why eFSE animals with poor cognitive outcomes fail to exhibit increased CA1 slow gamma amplitude during active avoidance epochs typically associated with memory recall (Barry et al., 2020). More experiments are needed to understand how eFSE might induce changes to processes affecting the generation and timing of dentate spikes as well as their governance of CA1 gamma oscillations (Dvorak et al., 2021).

CFC analysis reinforces our hypothesis that eFSE alters both intrahippocampal connectivity and hippocampal connectivity with the neocortex. Recent work has shown that decreased theta-gamma coupling can serve as a biomarker of impaired local inhibition and epileptogenesis in an animal model of Dravet syndrome (Jansen et al., 2021). The relevance of CFC for cognition is supported by a recent study by Fernandez-Ruiz et al. showing that the medial and lateral entorhinal cortices communicate with hippocampus via distinct frequencies within the gamma bandwidth. Optogenetic MEC perturbation disrupted fast gamma-mediated spatial processing while LEC perturbation disrupted slow gamma-mediated object processing (Fernandez-Ruiz et al., 2021). These findings map well to the proposed roles of the LEC in object processing (LEC=“what”) (Knierim et al., 2014; Mishkin et al., 1983; Wang et al., 2018) and of the MEC in spatial processing (MEC=“where”) (Butler et al., 2019; Campbell et al., 2021; Goodale & Milner, 1992; Jacob et al., 2017). Our results show that gamma coupling to slow oscillations in CTL rats at the OML and MML, which receive inputs from the LEC and MEC respectively, occurs in two different frequencies of gamma (Figure 10e). In eFSE rats, this distinction is lost and information from EC to hippocampus converges onto both synapses at one frequency range within the gamma bandwidth. If gamma frequency coupling in the DG is necessary for segregating LEC and MEC inputs to the cell layer via OML and MML inputs, the eFSE DG effectively consolidates two cortical information streams into one.

We suggest that structural changes disrupting the neocortical-hippocampal generation of theta oscillations (Inostroza et al., 2013; Laurent et al., 2015) and gamma-mediated segregation of hippocampal cell assemblies (Fernandez-Ruiz et al., 2021) could derail communication within and between the hippocampus and neocortex, chronically affecting post-synaptic information processing in neocortical-hippocampal circuit operations. This form of discoordination is distinct from inter-ictal epileptiform discharges (IEDs) found in most seizure models. IEDs can transiently interfere with signal coordination within and between the hippocampus and the neocortex, disrupting encoding or the recall of information (Gelinas et al., 2016; Kleen et al., 2010; Kleen et al., 2013). A recent study by Getz et al. illustrates this point in the Pten knockout model of autism spectrum disorder, where dendritic overgrowth and somatic hyperexcitability localized to ~30% of DG granule cells was sufficient for causing spatial cognitive deficits (Getz et al., 2022). Pharmacologically preventing dendritic overgrowth during development, rectified this cognitive deficit, allowing for the flexible association of an object cue with novel hidden goal locations. Similarly, preventing gene transcription silencing processes immediately post eFSE induction blocked DG dendritic overgrowth and associated spatial deficits in the active avoidance task (Patterson et al., 2017).

Finally, the study demonstrates the utility of stimulation-elicited slow theta oscillations under urethane as an assay of neocortical-hippocampal circuit function. The high spatial resolution of CSD mapping along the CA1 and DG somatodendritic axes allowed for detailed measurements of the properties and efficacy of synaptic inputs, signal integration, and propagation. Apart from the absence of significant modulation by fast or medium gamma at SLM and OML inputs under urethane, the depth profile of slow theta and gamma signaling measures in urethane anesthetized rats generally resembled previous reports in freely moving rats with 7–8 Hz dominant theta signals (Fernandez-Ruiz et al., 2021; Schomburg et al., 2014). Importantly, we were able to find some evidence of differential theta and gamma coupling between the OML and MML in control animals that were absent in eFSE animals. Whereas OML and MML are differentially coupled relative to 40 Hz in CTL, they are uniformly coupled to 25 Hz in eFSE. This might indicate, at least in controls, that some elements of a general entorhinal-hippocampus coding scheme for synaptic inputs can persist at specific dendritic compartments under urethane. The principal challenge to potential comparisons between urethane theta and freely moving animals is the fundamental observation of cholinergic dependence of slow theta under urethane in comparison to 7–8 Hz mobility theta (Dickson et al., 1994; Kramis et al., 1975). However, our previous report noting the dysfunctionality of CA1 slow gamma during slow immobility theta in eFSE rats with poor cognitive outcomes in active avoidance (Barry et al., 2020), is consistent with the poor coupling of CA1 slow theta and slow gamma shown here. This reinforces the notion that memory and physiology dynamics during immobility, rather than solely during mobility, are important to consider in both basic and translational experiments (Pofahl et al., 2021). Future experiments should therefore analyze putative differences in hippocampal cholinergic modulation in eFSE animals and whether these differences could also explain sex differences in cortico-hippocampal coordination and cognitive outcomes post early-life seizures (Kloc et al., 2022; Niedecker et al., 2021). A direct comparison between freely moving and urethane conditions could be revealing as even though parvalbumin-positive neurons dominate hippocampal mobility theta, nicotinic and muscarinic receptors may still play a role in the septohippocampal modulation of theta frequency and amplitude, either directly or indirectly through septal GABAergic or glutamatergic neurons (Dannenberg et al., 2015; Gu & Yakel, 2022).

Experiments using urethane anesthesia have established physiological throughput mechanisms in both the hippocampus (Lockmann et al., 2016) and EC (Dickson et al., 1994). More importantly, they have established the temporal organization of hippocampal excitatory and inhibitory neurons (Klausberger et al., 2003; Klausberger & Somogyi, 2008) and the temporal structure of pathway-specific field potentials (Fernandez-Ruiz et al., 2012). Our current results reinforce the notion that the tail pinch assay under urethane is also useful in translational models where severe cognitive deficits and the inability to perform a given task, or changes in motor output, preclude direct electrophysiological comparisons between model and control animals during behavior.

## 5 | CONCLUSIONS

These translational results support our hypothesis that eFSE-induced alteration of hippocampal physiology and structure affects the reception and propagation of neocortical-hippocampal signal projections along the CA1 and DG somatodendritic axes. Based on these observations, we propose a novel mechanism of eFSE cognitive deficit where post-synaptic entorhinal-hippocampal signal integration failure represents a chronic information processing challenge in systems responsible for spatial processing. The congruence of structural and synaptic input changes provoked by acquired or genetic perturbations raises the possibility that preventing these physiological changes, by epigenomic or pharmacological interventions (Getz et al., 2022; Patterson et al., 2017), could mitigate the spatial cognitive deficits observed here and in prior work (Barry et al., 2016, 2020; Kloc et al., 2022). Our results set a foundation for addressing whether the restoration of dendritic structure or the hippocampal synaptic coding mechanisms by theta and gamma oscillations, or both, are sufficient for preventing cognitive deficits associated with developmental pathophysiology. These studies could also provide mechanistic insights into sex differences in cognitive outcomes following early life seizures (Kloc et al., 2022; Niedecker et al., 2021) and test whether maintenance of cortico-hippocampal coordination post eFSE spares females from cognitive impairment.

## Supplementary Material

Supplemental Figures

Supplemental Tables

## Data Availability

Data available on request from the authors
