# Supplementary material for: Spatial learning impairments and discoordination of entorhinal-hippocampal circuit coding following prolonged febrile seizures
Source: Hippocampus. Author manuscript; Available in PMC 2024 Aug 1. (PMC10529121; doi:10.1002/hipo.23541)
Supplement: Supplemental Figures [file NIHMS1913455-supplement-Supplemental_Figures.pdf]

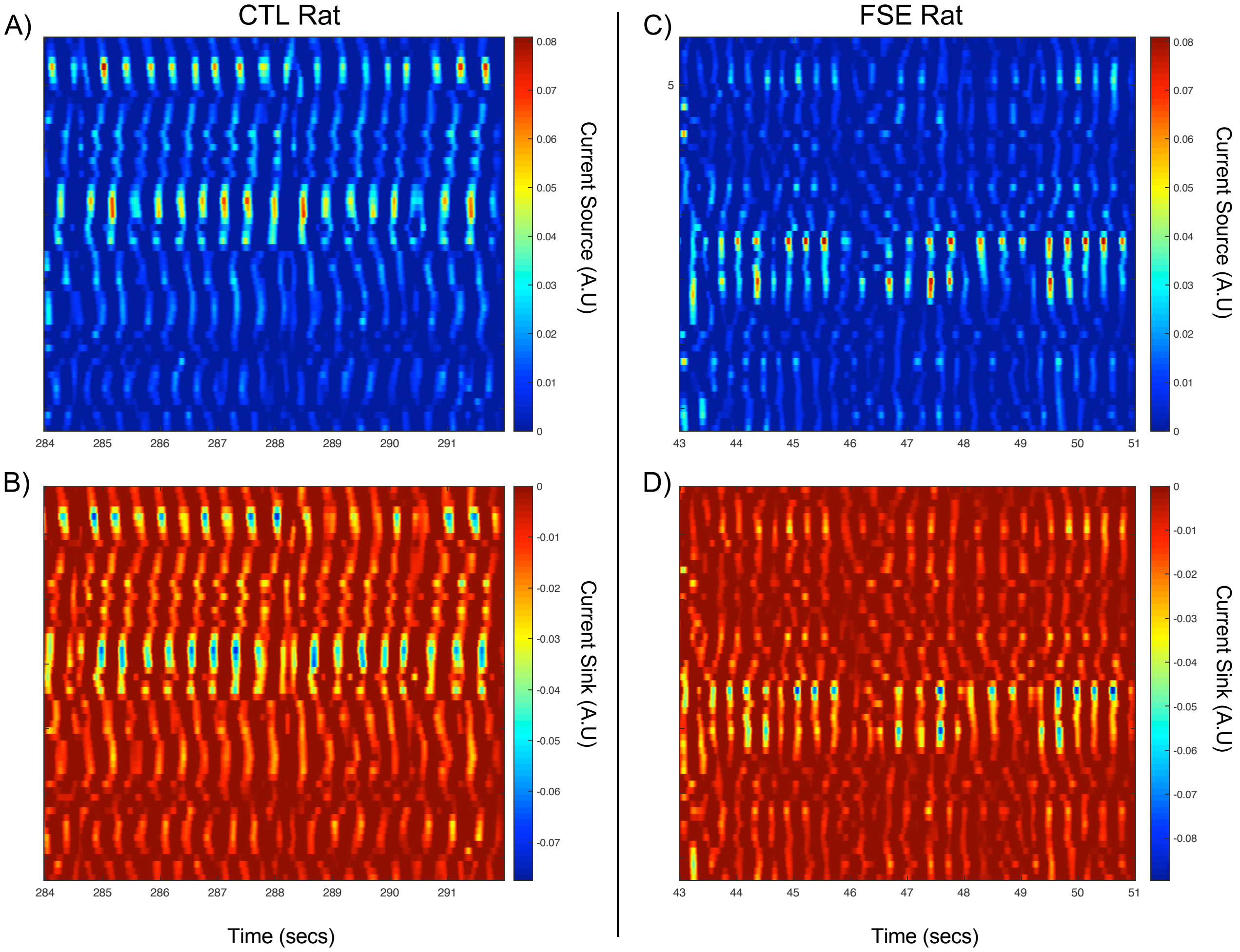
A)
CTL Rat
Current Source (A.U)
0.08
0.07
0.06
0.05
0.04
0.03
0.02
0.01
0
284
285
286
287
288
289
290
291
B)
Current Sink (A.U)
0
-0.01
-0.02
-0.03
-0.04
-0.05
-0.06
-0.07
Time (secs)
C)
FSE Rat
5
43
44
45
46
47
48
49
50
51
D)
-0.08
Time (secs)

Supp. Fig. 1

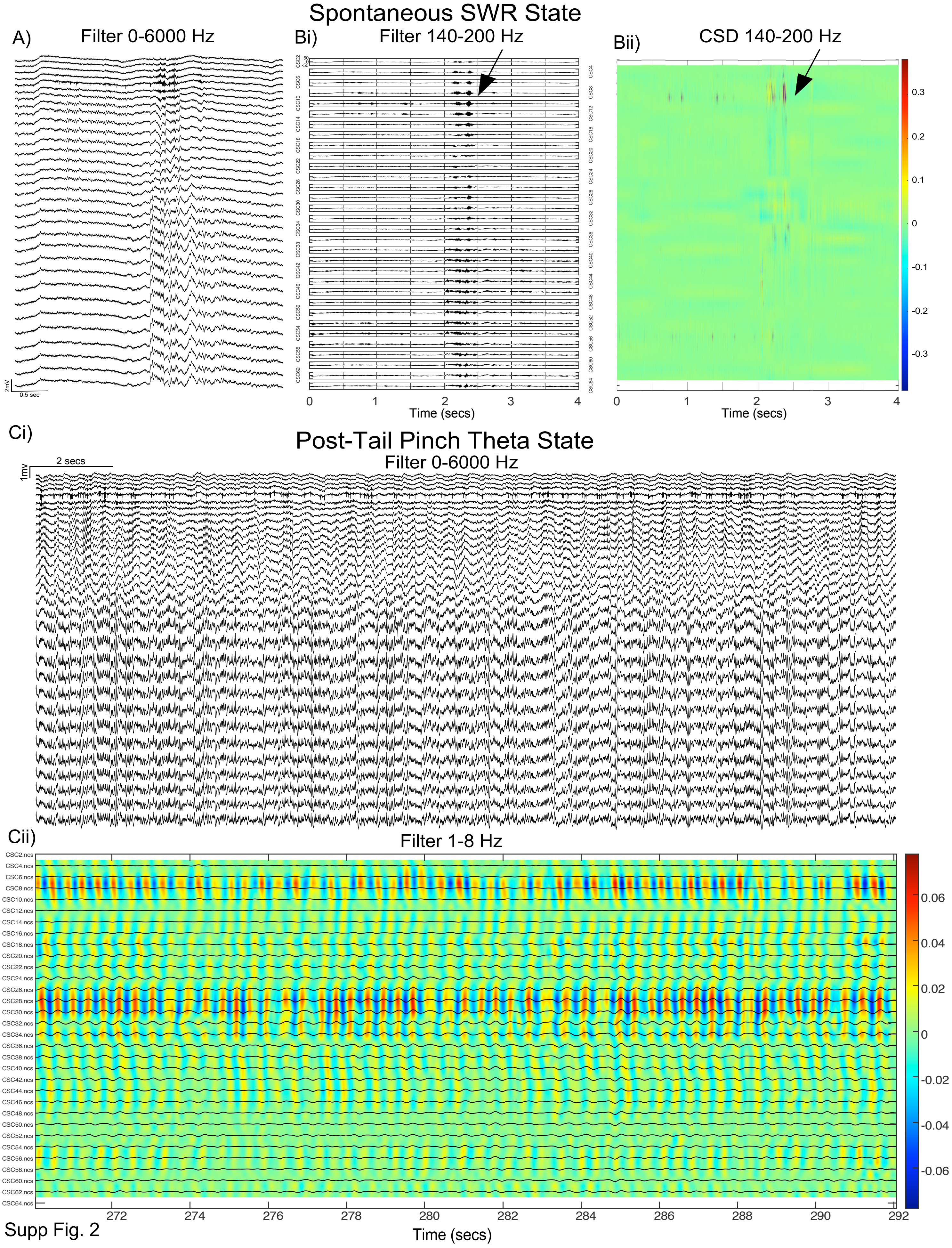
Spontaneous SWR State
A)
Filter 0-6000 Hz
2mV
0.5 sec
Bi)
Filter 140-200 Hz
Time (secs)
Bii)
CSD 140-200 Hz
Time (secs)
0.3
0.2
0.1
0
-0.1
-0.2
-0.3
Ci)
Post-Tail Pinch Theta State
Filter 0-6000 Hz
2 secs
1mv
Cii)
Filter 1-8 Hz
Time (secs)
0.06
0.04
0.02
0
-0.02
-0.04
-0.06

Supp Fig. 2

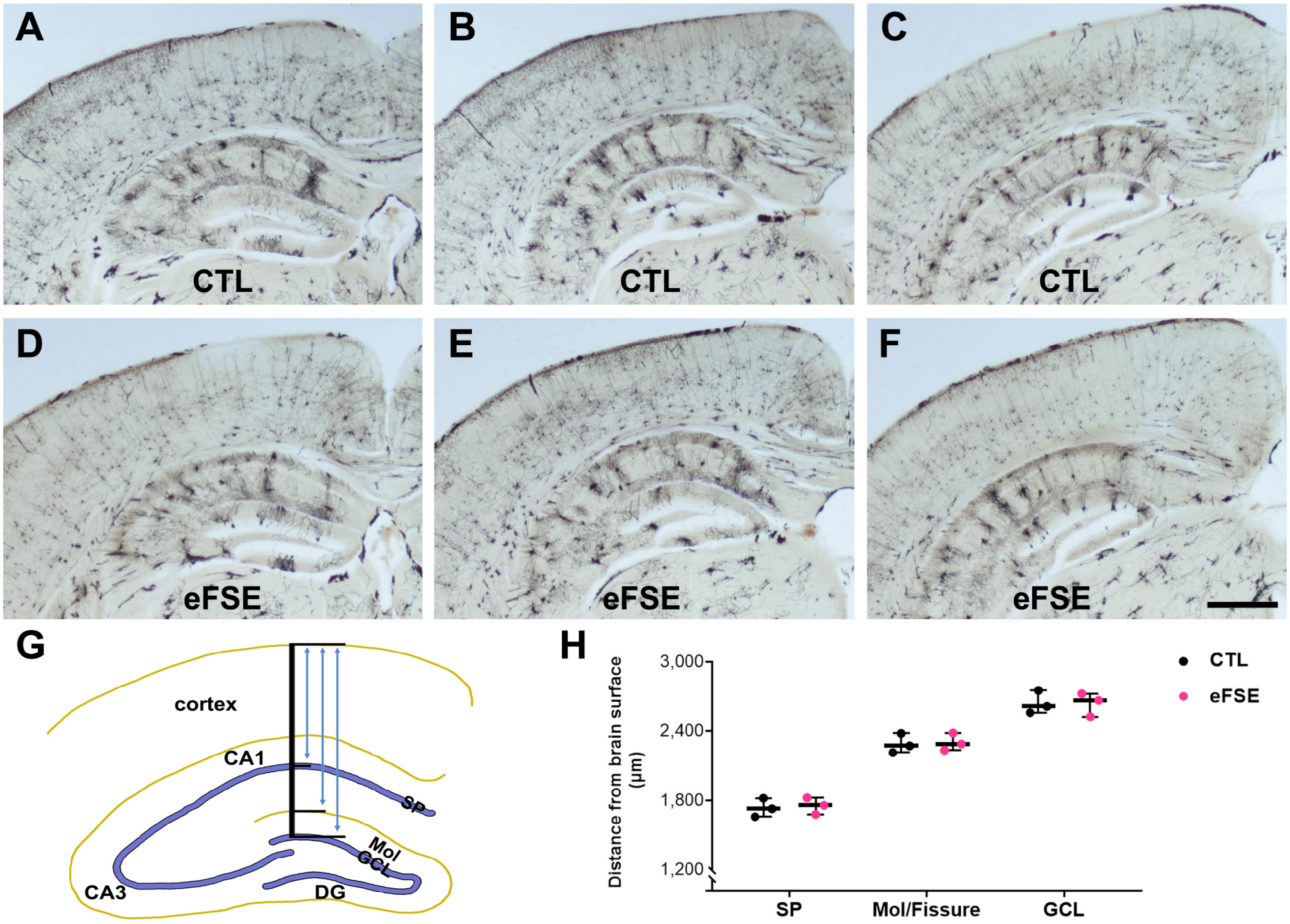
A
CTL
B
CTL
C
CTL
D
eFSE
E
eFSE
F
eFSE
G
cortex
CA1
SP
Mol
GCL
CA3
DG
H
Distance from brain surface (µm)
3,000
2,400
1,800
1,200
SP
Mol/Fissure
GCL
CTL
eFSE

Supp. Fig. 3

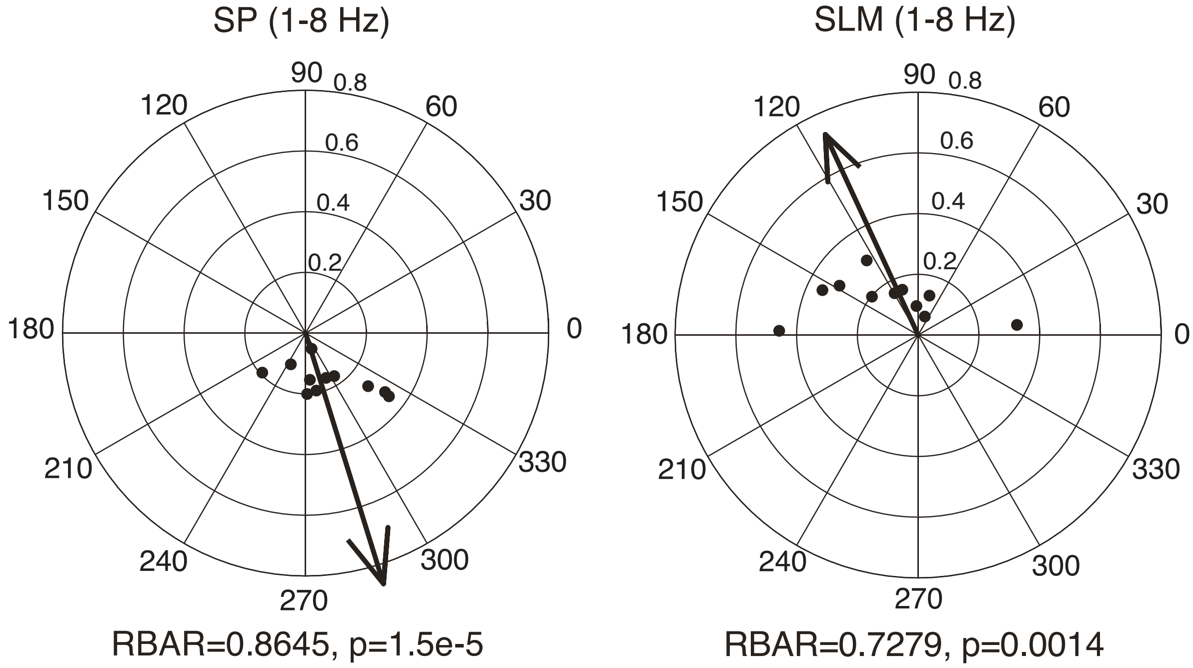
SP (1-8 Hz)
90
120
60
150
30
180
0
210
330
240
300
270
0.2
0.4
0.6
0.8
RBAR=0.8645, p=1.5e-5
SLM (1-8 Hz)
90
120
60
150
30
180
0
210
330
240
300
270
0.2
0.4
0.6
0.8
RBAR=0.7279, p=0.0014

Supp. Fig. 4

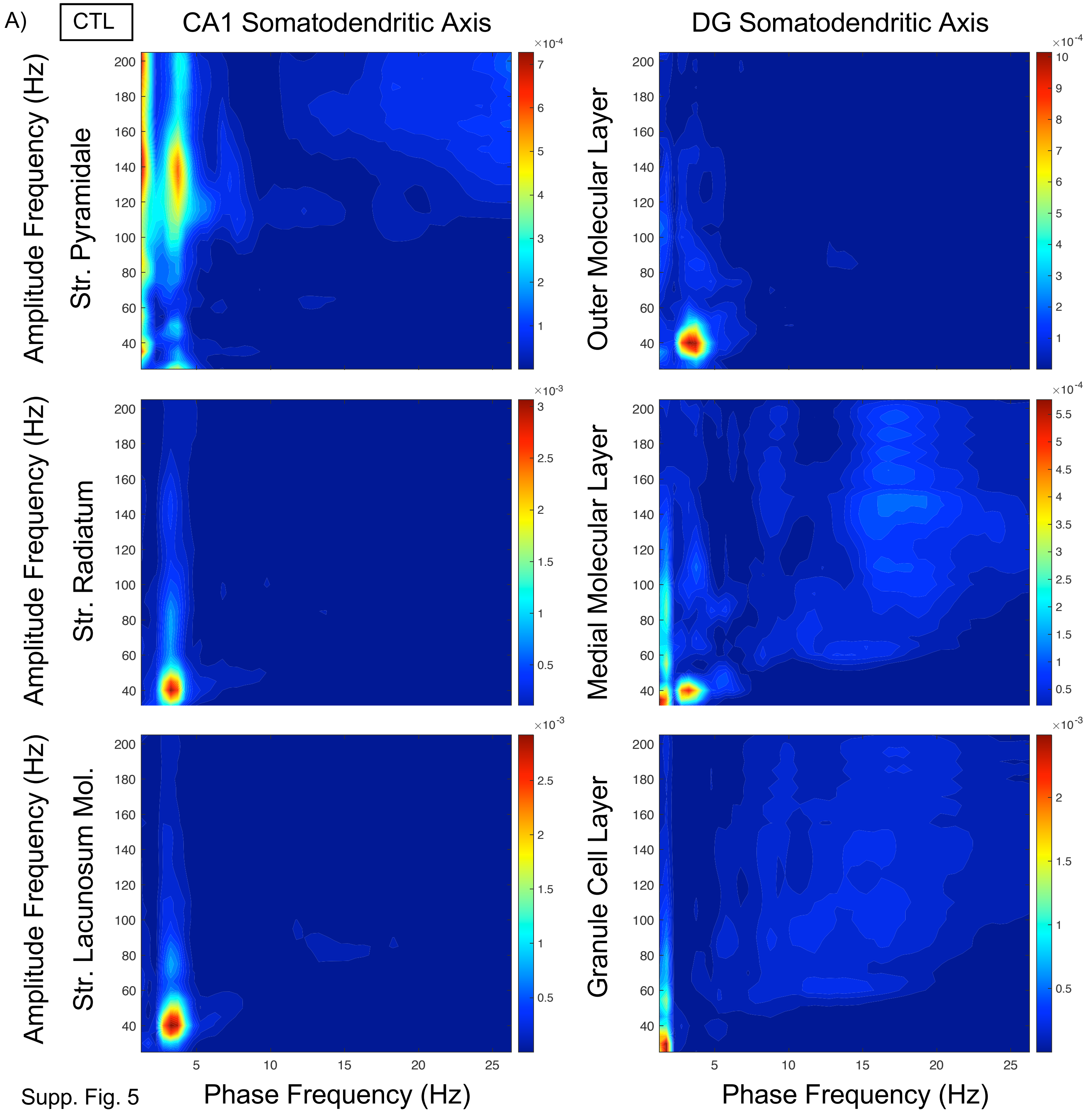
A)
CTL
CA1 Somatodendritic Axis
DG Somatodendritic Axis
Amplitude Frequency (Hz)
Str. Pyramidale
Str. Radiatum
Str. Lacunosum Mol.
Outer Molecular Layer
Medial Molecular Layer
Granule Cell Layer
Phase Frequency (Hz)
×10^-4
×10^-3

Supp. Fig. 5
