# Supplementary material for: Spatial learning impairments and discoordination of entorhinal-hippocampal circuit coding following prolonged febrile seizures
Source: Hippocampus. Author manuscript; Available in PMC 2024 Aug 1. (PMC10529121; doi:10.1002/hipo.23541)
Supplement: Supplemental Tables [file NIHMS1913455-supplement-Supplemental_Tables.docx]

**Supplemental Results**

**Signal Properties**

Supplemental Table 1: GEE results of group x depth interaction analyzing CSD source in CTL and FSE animals. Statistical Comparator (0a) = Ch 5 (SP Layer in CTL). These results are also plotted in Fig. 6Cii.

| Parameter | Hypothesis Test | |  |
| --- | --- | --- | --- |
|  | Wald Chi-Square | Sig. |  |
| [Group =eFSE] * [Channel=1.0] | 1.773 | 0.1830 |  |
| [Group =eFSE] * [Channel=2.0] | 36.223 | 0.0000 |  |
| [Group =eFSE] * [Channel=3.0] | 1.39 | 0.2380 |  |
| [Group =eFSE] * [Channel=4.0] | 11.33 | 0.0010 |  |
| [Group =eFSE] * [Channel=5.0] | 0.451 | 0.5020 |  |
| [Group =eFSE] * [Channel=6.0] | 6.591 | 0.0100 |  |
| [Group =eFSE] * [Channel=7.0] | 10.837 | 0.0010 |  |
| [Group =eFSE] * [Channel=8.0] | 8.836 | 0.0030 |  |
| [Group =eFSE] * [Channel=9.0] | 3.19 | 0.0740 |  |
| [Group =eFSE] * [Channel=10.0] | 16.305 | 0.0000 |  |
| [Group =eFSE] * [Channel=11.0] | 0.258 | 0.6120 |  |
| [Group =eFSE] * [Channel=12.0] | 4.847 | 0.0280 |  |
| [Group =eFSE] * [Channel=13.0] | 9.858 | 0.0020 |  |
| [Group =eFSE] * [Channel=14.0] | 13.869 | 0.0000 |  |
| [Group =eFSE] * [Channel=15.0] | 3.1 | 0.0780 |  |
| [Group =eFSE] * [Channel=16.0] | 31.755 | 0.0000 |  |
| [Group =eFSE] * [Channel=17.0] | 5.175 | 0.0230 |  |
| [Group =eFSE] * [Channel=18.0] | 8.807 | 0.0030 |  |
| [Group =eFSE] * [Channel=19.0] | 0.465 | 0.4950 |  |
| [Group =eFSE] * [Channel=20.0] | 30.864 | 0.0000 |  |
| [Group =eFSE] * [Channel=21.0] | 0.51 | 0.4750 |  |
| [Group =eFSE] * [Channel=22.0] | 4.125 | 0.0420 |  |
| [Group =eFSE] * [Channel=23.0] | 15.504 | 0.0000 |  |
| [Group =eFSE] * [Channel=24.0] | 8.316 | 0.0040 |  |
| [Group =eFSE] * [Channel=25.0] | 0.392 | 0.5310 |  |
| [Group =eFSE] * [Channel=26.0] | 0.577 | 0.4480 |  |
| [Group =eFSE] * [Channel=27.0] | 0.523 | 0.4690 |  |
| [Group =eFSE] * [Channel=28.0] | 2.518 | 0.1130 |  |
| [Group =eFSE] * [Channel=29.0] | 0.274 | 0.6010 |  |
| [Group =eFSE] * [Channel=30.0] | 1.189 | 0.2760 |  |
| [Group =eFSE] * [Channel=31.0] | 1.119 | 0.2900 |  |
| [Group =eFSE] * [Channel=32.0] | 0.778 | 0.3780 |  |
| [Group =eFSE] * [Channel=33.0] | 0.139 | 0.7090 |  |
| [Group =eFSE] * [Channel=34.0] | 1.665 | 0.1970 |  |
| [Group =eFSE] * [Channel=35.0] | 0.854 | 0.3550 |  |
| [Group =eFSE] * [Channel=36.0] | 2.293 | 0.1300 |  |
| [Group =eFSE] * [Channel=37.0] | 2.008 | 0.1560 |  |
| [Group =eFSE] * [Channel=38.0] | 4.879 | 0.0270 |  |
| [Group =eFSE] * [Channel=39.0] | 3.959 | 0.0470 |  |
| [Group =eFSE] * [Channel=40.0] | 7.153 | 0.0070 |  |
| [Group =eFSE] * [Channel=41.0] | 6.878 | 0.0090 |  |
| [Group =eFSE] * [Channel=42.0] | 14.305 | 0.0000 |  |
| [Group =eFSE] * [Channel=43.0] | 1.171 | 0.2790 |  |
| [Group =eFSE] * [Channel=44.0] | 36.216 | 0.0000 |  |
| [Group =eFSE] * [Channel=45.0] | 2.25 | 0.1340 |  |
| [Group =eFSE] * [Channel=46.0] | 32.248 | 0.0000 |  |
| [Group =eFSE] * [Channel=47.0] | 3.377 | 0.0660 |  |
| [Group =eFSE] * [Channel=48.0] | 2.491 | 0.1140 |  |
| [Group =CTL] * [Channel=1.0] | 234.957 | 0.0000 |  |
| [Group =CTL] * [Channel=2.0] | 269.687 | 0.0000 |  |
| [Group =CTL] * [Channel=3.0] | 39.488 | 0.0000 |  |
| [Group =CTL] * [Channel=4.0] | 157.904 | 0.0000 |  |
| [Group =CTL] * [Channel=6.0] | 14.866 | 0.0000 |  |
| [Group =CTL] * [Channel=7.0] | 16.302 | 0.0000 |  |
| [Group =CTL] * [Channel=8.0] | 108.732 | 0.0000 |  |
| [Group =CTL] * [Channel=9.0] | 54.779 | 0.0000 |  |
| [Group =CTL] * [Channel=10.0] | 65.257 | 0.0000 |  |
| [Group =CTL] * [Channel=11.0] | 60.999 | 0.0000 |  |
| [Group =CTL] * [Channel=12.0] | 106.675 | 0.0000 |  |
| [Group =CTL] * [Channel=13.0] | 51.139 | 0.0000 |  |
| [Group =CTL] * [Channel=14.0] | 130.003 | 0.0000 |  |
| [Group =CTL] * [Channel=15.0] | 132.472 | 0.0000 |  |
| [Group =CTL] * [Channel=16.0] | 184.736 | 0.0000 |  |
| [Group =CTL] * [Channel=17.0] | 106.343 | 0.0000 |  |
| [Group =CTL] * [Channel=18.0] | 44.994 | 0.0000 |  |
| [Group =CTL] * [Channel=19.0] | 17.201 | 0.0000 |  |
| [Group =CTL] * [Channel=20.0] | 19.791 | 0.0000 |  |
| [Group =CTL] * [Channel=21.0] | 9.96 | 0.0020 |  |
| [Group =CTL] * [Channel=22.0] | 40.882 | 0.0000 |  |
| [Group =CTL] * [Channel=23.0] | 16.74 | 0.0000 |  |
| [Group =CTL] * [Channel=24.0] | 21.087 | 0.0000 |  |
| [Group =CTL] * [Channel=25.0] | 0.077 | 0.7820 |  |
| [Group =CTL] * [Channel=26.0] | 2.631 | 0.1050 |  |
| [Group =CTL] * [Channel=27.0] | 0.805 | 0.3690 |  |
| [Group =CTL] * [Channel=28.0] | 0.567 | 0.4510 |  |
| [Group =CTL] * [Channel=29.0] | 2.026 | 0.1550 |  |
| [Group =CTL] * [Channel=30.0] | 2.335 | 0.1270 |  |
| [Group =CTL] * [Channel=31.0] | 0.241 | 0.6240 |  |
| [Group =CTL] * [Channel=32.0] | 7.513 | 0.0060 |  |
| [Group =CTL] * [Channel=33.0] | 20.185 | 0.0000 |  |
| [Group =CTL] * [Channel=34.0] | 24.688 | 0.0000 |  |
| [Group =CTL] * [Channel=35.0] | 38.987 | 0.0000 |  |
| [Group =CTL] * [Channel=36.0] | 20.574 | 0.0000 |  |
| [Group =CTL] * [Channel=37.0] | 68.292 | 0.0000 |  |
| [Group =CTL] * [Channel=38.0] | 31.484 | 0.0000 |  |
| [Group =CTL] * [Channel=39.0] | 12.046 | 0.0010 |  |
| [Group =CTL] * [Channel=40.0] | 16.175 | 0.0000 |  |
| [Group =CTL] * [Channel=41.0] | 19.058 | 0.0000 |  |
| [Group =CTL] * [Channel=42.0] | 64.446 | 0.0000 |  |
| [Group =CTL] * [Channel=43.0] | 143.8 | 0.0000 |  |
| [Group =CTL] * [Channel=44.0] | 59.609 | 0.0000 |  |
| [Group =CTL] * [Channel=45.0] | 11.247 | 0.0010 |  |
| [Group =CTL] * [Channel=46.0] | 16.185 | 0.0000 |  |
| [Group =CTL] * [Channel=47.0] | 18.107 | 0.0000 |  |
| [Group =CTL] * [Channel=48.0] | 21.747 |  |  |
| [Group =CTL] * [Channel=5.0] |  |  |  |

Supplemental Table 2. TPS (CTL-5Hz) mean frequency group x depth GEE analysis using group x -depth comparison.

| Parameter | B | Hypothesis Test | |  |
| --- | --- | --- | --- | --- |
|  |  | Wald Chi-Square | Sig. |  |
| [Group=CTL] * [Depth=0] | -0.146 | 3.303 | 0.069 |  |
| [Group=FSE] * [Depth=0] | 0^a^ |  |  |  |
| [Group=CTL] * [Depth=40] | -0.140 | 3.047 | 0.081 |  |
| [Group=FSE] * [Depth=40] | 0^a^ |  |  |  |
| [Group=CTL] * [Depth=60] | -0.142 | 2.979 | 0.084 |  |
| [Group=FSE] * [Depth=60] | 0^a^ |  |  |  |
| [Group=CTL] * [Depth=80] | -0.138 | 2.757 | 0.097 |  |
| [Group=FSE] * [Depth=80] | 0^a^ |  |  |  |
| [Group=CTL] * [Depth=100] | -0.135 | 2.467 | 0.116 |  |
| [Group=FSE] * [Depth=100] | 0^a^ |  |  |  |
| [Group=CTL] * [Depth=120] | -0.087 | 2.144 | 0.143 |  |
| [Group=FSE] * [Depth=120] | 0^a^ |  |  |  |
| [Group=CTL] * [Depth=140] | -0.139 | 2.270 | 0.132 |  |
| [Group=FSE] * [Depth=140] | 0^a^ |  |  |  |
| [Group=CTL] * [Depth=160] | -0.135 | 2.235 | 0.135 |  |
| [Group=FSE] * [Depth=160] | 0^a^ |  |  |  |
| [Group=CTL] * [Depth=180] | -0.133 | 2.108 | 0.147 |  |
| [Group=FSE] * [Depth=180] | 0^a^ |  |  |  |
| [Group=CTL] * [Depth=200] | -0.142 | 2.521 | 0.112 |  |
| [Group=FSE] * [Depth=200] | 0^a^ |  |  |  |
| [Group=CTL] * [Depth=220] | -0.151 | 2.715 | 0.099 |  |
| [Group=FSE] * [Depth=220] | 0^a^ |  |  |  |
| [Group=CTL] * [Depth=240] | -0.155 | 3.440 | 0.064 |  |
| [Group=FSE] * [Depth=240] | 0^a^ |  |  |  |
| [Group=CTL] * [Depth=260] | -0.154 | 4.308 | 0.038 |  |
| [Group=FSE] * [Depth=260] | 0^a^ |  |  |  |
| [Group=CTL] * [Depth=280] | -0.162 | 7.260 | 0.007 |  |
| [Group=FSE] * [Depth=280] | 0^a^ |  |  |  |
| [Group=CTL] * [Depth=300] | -0.164 | 12.943 | 0.000 |  |
| [Group=FSE] * [Depth=300] | 0^a^ |  |  |  |
| [Group=CTL] * [Depth=320] | -0.160 | 18.040 | 0.000 |  |
| [Group=FSE] * [Depth=320] | 0^a^ |  |  |  |
| [Group=CTL] * [Depth=340] | -0.140 | 10.504 | 0.001 |  |
| [Group=FSE] * [Depth=340] | 0^a^ |  |  |  |
| [Group=CTL] * [Depth=360] | -0.135 | 6.087 | 0.014 |  |
| [Group=FSE] * [Depth=360] | 0^a^ |  |  |  |
| [Group=CTL] * [Depth=380] | -0.121 | 3.158 | 0.076 |  |
| [Group=FSE] * [Depth=380] | 0^a^ |  |  |  |
| [Group=CTL] * [Depth=400] | -0.107 | 1.794 | 0.180 |  |
| [Group=FSE] * [Depth=400] | 0^a^ |  |  |  |
| [Group=CTL] * [Depth=420] | -0.107 | 1.371 | 0.242 |  |
| [Group=FSE] * [Depth=420] | 0^a^ |  |  |  |
| [Group=CTL] * [Depth=440] | -0.086 | 2.126 | 0.145 |  |
| [Group=FSE] * [Depth=440] | 0^a^ |  |  |  |
| [Group=CTL] * [Depth=460] | -0.108 | 1.057 | 0.304 |  |
| [Group=FSE] * [Depth=460] | 0^a^ |  |  |  |
| [Group=CTL] * [Depth=480] | -0.106 | 0.904 | 0.342 |  |
| [Group=FSE] * [Depth=480] | 0^a^ |  |  |  |
| [Group=CTL] * [Depth=500] | -0.109 | 0.894 | 0.344 |  |
| [Group=FSE] * [Depth=500] | 0^a^ |  |  |  |
| [Group=CTL] * [Depth=520] | -0.118 | 1.034 | 0.309 |  |
| [Group=FSE] * [Depth=520] | 0^a^ |  |  |  |
| [Group=CTL] * [Depth=540] | -0.111 | 0.908 | 0.341 |  |
| [Group=FSE] * [Depth=540] | 0^a^ |  |  |  |
| [Group=CTL] * [Depth=560] | -0.105 | 0.765 | 0.382 |  |
| [Group=FSE] * [Depth=560] | 0^a^ |  |  |  |
| [Group=CTL] * [Depth=600] | -0.098 | 0.677 | 0.411 |  |
| [Group=FSE] * [Depth=600] | 0^a^ |  |  |  |
| [Group=CTL] * [Depth=620] | -0.090 | 0.582 | 0.446 |  |
| [Group=FSE] * [Depth=620] | 0^a^ |  |  |  |
| [Group=CTL] * [Depth=640] | -0.085 | 0.493 | 0.483 |  |
| [Group=FSE] * [Depth=640] | 0^a^ |  |  |  |
| [Group=CTL] * [Depth=660] | -0.092 | 0.606 | 0.436 |  |
| [Group=FSE] * [Depth=660] | 0^a^ |  |  |  |
| [Group=CTL] * [Depth=680] | -0.098 | 0.681 | 0.409 |  |
| [Group=FSE] * [Depth=680] | 0^a^ |  |  |  |
| [Group=CTL] * [Depth=700] | -0.107 | 0.763 | 0.382 |  |
| [Group=FSE] * [Depth=700] | 0^a^ |  |  |  |
| [Group=CTL] * [Depth=720] | -0.108 | 0.780 | 0.377 |  |
| [Group=FSE] * [Depth=720] | 0^a^ |  |  |  |
| [Group=CTL] * [Depth=740] | -0.111 | 0.792 | 0.374 |  |
| [Group=FSE] * [Depth=740] | 0^a^ |  |  |  |
| [Group=CTL] * [Depth=760] | -0.112 | 0.769 | 0.380 |  |
| [Group=FSE] * [Depth=760] | 0^a^ |  |  |  |
| [Group=CTL] * [Depth=780] | -0.111 | 0.729 | 0.393 |  |
| [Group=FSE] * [Depth=780] | 0^a^ |  |  |  |
| [Group=CTL] * [Depth=800] | -0.111 | 0.754 | 0.385 |  |
| [Group=FSE] * [Depth=800] | 0^a^ |  |  |  |
| [Group=CTL] * [Depth=820] | -0.105 | 0.712 | 0.399 |  |
| [Group=FSE] * [Depth=820] | 0^a^ |  |  |  |
| [Group=CTL] * [Depth=840] | -0.111 | 0.859 | 0.354 |  |
| [Group=FSE] * [Depth=840] | 0^a^ |  |  |  |
| [Group=CTL] * [Depth=860] | -0.111 | 0.911 | 0.340 |  |
| [Group=FSE] * [Depth=860] | 0^a^ |  |  |  |
| [Group=CTL] * [Depth=880] | -0.115 | 1.063 | 0.302 |  |
| [Group=FSE] * [Depth=880] | 0^a^ |  |  |  |
| [Group=CTL] * [Depth=900] | -0.114 | 1.159 | 0.282 |  |
| [Group=FSE] * [Depth=900] | 0^a^ |  |  |  |
| [Group=CTL] * [Depth=920] | -0.112 | 1.185 | 0.276 |  |
| [Group=FSE] * [Depth=920] | 0^a^ |  |  |  |
| [Group=CTL] * [Depth=940] | -0.121 | 1.489 | 0.222 |  |
| [Group=FSE] * [Depth=940] | 0^a^ |  |  |  |
| [Group=CTL] * [Depth=960] | -0.118 | 1.588 | 0.208 |  |
| [Group=FSE] * [Depth=960] | 0^a^ |  |  |  |
| [Group=CTL] * [Depth=1000] | -0.130 | 2.327 | 0.127 |  |
| [Group=FSE] * [Depth=1000] | 0^a^ |  |  |  |
| [Group=CTL] * [Depth=1020] | -0.138 | 2.947 | 0.086 |  |
| [Group=FSE] * [Depth=1020] | 0^a^ |  |  |  |
| [Group=CTL] * [Depth=1040] | -0.145 | 3.757 | 0.053 |  |
| [Group=FSE] * [Depth=1040] | 0^a^ |  |  |  |
| [Group=CTL] * [Depth=1060] | -0.135 | 3.315 | 0.069 |  |
| [Group=FSE] * [Depth=1060] | 0^a^ |  |  |  |
| [Group=CTL] * [Depth=1080] | -0.143 | 3.755 | 0.053 |  |
| [Group=FSE] * [Depth=1080] | 0^a^ |  |  |  |
| [Group=CTL] * [Depth=1100] | -0.135 | 3.614 | 0.057 |  |
| [Group=FSE] * [Depth=1100] | 0^a^ |  |  |  |
| [Group=CTL] * [Depth=1120] | -0.137 | 3.902 | 0.048 |  |
| [Group=FSE] * [Depth=1120] | 0^a^ |  |  |  |
| [Group=CTL] * [Depth=1140] | -0.140 | 3.982 | 0.046 |  |
| [Group=FSE] * [Depth=1140] | 0^a^ |  |  |  |
| [Group=CTL] * [Depth=1180] | -0.131 | 3.997 | 0.046 |  |
| [Group=FSE] * [Depth=1180] | 0^a^ |  |  |  |
| [Group=CTL] * [Depth=1200] | -0.133 | 4.028 | 0.045 |  |
| [Group=FSE] * [Depth=1200] | 0^a^ |  |  |  |
| [Group=CTL] * [Depth=1220] | -0.119 | 3.058 | 0.080 |  |
| [Group=FSE] * [Depth=1220] | 0^a^ |  |  |  |
| [Group=CTL] * [Depth=1240] | -0.131 | 3.703 | 0.054 |  |
| [Group=FSE] * [Depth=1240] | 0^a^ |  |  |  |

Supplemental Table 3. TPS Peak TPS Power group x depth GEE analysis.

| Parameter | B | Hypothesis Test | |
| --- | --- | --- | --- |
|  |  | Wald Chi-Square | Sig. |
| [Group=CTL] * [Depth=0] | 0.017 | 1.852 | 0.174 |
| [Group=FSE] * [Depth=0] | 0^a^ |  |  |
| [Group=CTL] * [Depth=20] | 0.016 | 1.624 | 0.203 |
| [Group=FSE] * [Depth=20] | 0^a^ |  |  |
| [Group=CTL] * [Depth=40] | 0.017 | 1.685 | 0.194 |
| [Group=FSE] * [Depth=40] | 0^a^ |  |  |
| [Group=CTL] * [Depth=60] | 0.017 | 1.933 | 0.164 |
| [Group=FSE] * [Depth=60] | 0^a^ |  |  |
| [Group=CTL] * [Depth=80] | 0.017 | 1.803 | 0.179 |
| [Group=FSE] * [Depth=80] | 0^a^ |  |  |
| [Group=CTL] * [Depth=100] | 0.016 | 1.710 | 0.191 |
| [Group=FSE] * [Depth=100] | 0^a^ |  |  |
| [Group=CTL] * [Depth=120] | 0.014 | 2.038 | 0.153 |
| [Group=FSE] * [Depth=120] | 0^a^ |  |  |
| [Group=CTL] * [Depth=140] | 0.016 | 1.830 | 0.176 |
| [Group=FSE] * [Depth=140] | 0^a^ |  |  |
| [Group=CTL] * [Depth=160] | 0.016 | 1.796 | 0.180 |
| [Group=FSE] * [Depth=160] | 0^a^ |  |  |
| [Group=CTL] * [Depth=180] | 0.017 | 1.967 | 0.161 |
| [Group=FSE] * [Depth=180] | 0^a^ |  |  |
| [Group=CTL] * [Depth=200] | 0.019 | 2.918 | 0.088 |
| [Group=FSE] * [Depth=200] | 0^a^ |  |  |
| [Group=CTL] * [Depth=220] | 0.020 | 3.828 | 0.050 |
| [Group=FSE] * [Depth=220] | 0^a^ |  |  |
| [Group=CTL] * [Depth=240] | 0.019 | 3.989 | 0.046 |
| [Group=FSE] * [Depth=240] | 0^a^ |  |  |
| [Group=CTL] * [Depth=260] | 0.019 | 4.922 | 0.027 |
| [Group=FSE] * [Depth=260] | 0^a^ |  |  |
| [Group=CTL] * [Depth=280] | 0.015 | 3.698 | 0.054 |
| [Group=FSE] * [Depth=280] | 0^a^ |  |  |
| [Group=CTL] * [Depth=300] | 0.013 | 3.058 | 0.080 |
| [Group=FSE] * [Depth=300] | 0^a^ |  |  |
| [Group=CTL] * [Depth=320] | 0.011 | 1.767 | 0.184 |
| [Group=FSE] * [Depth=320] | 0^a^ |  |  |
| [Group=CTL] * [Depth=340] | 0.006 | 0.597 | 0.440 |
| [Group=FSE] * [Depth=340] | 0^a^ |  |  |
| [Group=CTL] * [Depth=360] | 0.005 | 0.373 | 0.542 |
| [Group=FSE] * [Depth=360] | 0^a^ |  |  |
| [Group=CTL] * [Depth=380] | 0.002 | 0.056 | 0.813 |
| [Group=FSE] * [Depth=380] | 0^a^ |  |  |
| [Group=CTL] * [Depth=400] | -0.002 | 0.129 | 0.719 |
| [Group=FSE] * [Depth=400] | 0^a^ |  |  |
| [Group=CTL] * [Depth=420] | -0.005 | 0.630 | 0.427 |
| [Group=FSE] * [Depth=420] | 0^a^ |  |  |
| [Group=CTL] * [Depth=440] | 0.015 | 2.219 | 0.136 |
| [Group=FSE] * [Depth=440] | 0^a^ |  |  |
| [Group=CTL] * [Depth=460] | -0.006 | 0.828 | 0.363 |
| [Group=FSE] * [Depth=460] | 0^a^ |  |  |
| [Group=CTL] * [Depth=480] | -0.007 | 1.229 | 0.268 |
| [Group=FSE] * [Depth=480] | 0^a^ |  |  |
| [Group=CTL] * [Depth=500] | -0.008 | 1.161 | 0.281 |
| [Group=FSE] * [Depth=500] | 0^a^ |  |  |
| [Group=CTL] * [Depth=520] | -0.008 | 0.857 | 0.354 |
| [Group=FSE] * [Depth=520] | 0^a^ |  |  |
| [Group=CTL] * [Depth=540] | -0.010 | 1.056 | 0.304 |
| [Group=FSE] * [Depth=540] | 0^a^ |  |  |
| [Group=CTL] * [Depth=560] | -0.011 | 1.427 | 0.232 |
| [Group=FSE] * [Depth=560] | 0^a^ |  |  |
| [Group=CTL] * [Depth=600] | -0.014 | 2.068 | 0.150 |
| [Group=FSE] * [Depth=600] | 0^a^ |  |  |
| [Group=CTL] * [Depth=620] | -0.014 | 2.067 | 0.150 |
| [Group=FSE] * [Depth=620] | 0^a^ |  |  |
| [Group=CTL] * [Depth=640] | -0.015 | 3.176 | 0.075 |
| [Group=FSE] * [Depth=640] | 0^a^ |  |  |
| [Group=CTL] * [Depth=660] | -0.013 | 2.894 | 0.089 |
| [Group=FSE] * [Depth=660] | 0^a^ |  |  |
| [Group=CTL] * [Depth=680] | -0.011 | 2.618 | 0.106 |
| [Group=FSE] * [Depth=680] | 0^a^ |  |  |
| [Group=CTL] * [Depth=700] | -0.008 | 1.394 | 0.238 |
| [Group=FSE] * [Depth=700] | 0^a^ |  |  |
| [Group=CTL] * [Depth=720] | -0.007 | 1.195 | 0.274 |
| [Group=FSE] * [Depth=720] | 0^a^ |  |  |
| [Group=CTL] * [Depth=740] | -0.005 | 0.715 | 0.398 |
| [Group=FSE] * [Depth=740] | 0^a^ |  |  |
| [Group=CTL] * [Depth=760] | -0.004 | 0.446 | 0.504 |
| [Group=FSE] * [Depth=760] | 0^a^ |  |  |
| [Group=CTL] * [Depth=780] | -0.004 | 0.324 | 0.569 |
| [Group=FSE] * [Depth=780] | 0^a^ |  |  |
| [Group=CTL] * [Depth=800] | -0.002 | 0.106 | 0.745 |
| [Group=FSE] * [Depth=800] | 0^a^ |  |  |
| [Group=CTL] * [Depth=820] | -0.002 | 0.067 | 0.795 |
| [Group=FSE] * [Depth=820] | 0^a^ |  |  |
| [Group=CTL] * [Depth=840] | 0.000 | 0.002 | 0.968 |
| [Group=FSE] * [Depth=840] | 0^a^ |  |  |
| [Group=CTL] * [Depth=860] | -1.573E-05 | 0.000 | 0.998 |
| [Group=FSE] * [Depth=860] | 0^a^ |  |  |
| [Group=CTL] * [Depth=880] | 0.000 | 0.001 | 0.971 |
| [Group=FSE] * [Depth=880] | 0^a^ |  |  |
| [Group=CTL] * [Depth=900] | 0.001 | 0.007 | 0.934 |
| [Group=FSE] * [Depth=900] | 0^a^ |  |  |
| [Group=CTL] * [Depth=920] | 0.001 | 0.009 | 0.923 |
| [Group=FSE] * [Depth=920] | 0^a^ |  |  |
| [Group=CTL] * [Depth=940] | 0.002 | 0.071 | 0.790 |
| [Group=FSE] * [Depth=940] | 0^a^ |  |  |
| [Group=CTL] * [Depth=960] | 0.003 | 0.142 | 0.707 |
| [Group=FSE] * [Depth=960] | 0^a^ |  |  |
| [Group=CTL] * [Depth=1000] | 0.005 | 0.431 | 0.512 |
| [Group=FSE] * [Depth=1000] | 0^a^ |  |  |
| [Group=CTL] * [Depth=1020] | 0.006 | 0.847 | 0.357 |
| [Group=FSE] * [Depth=1020] | 0^a^ |  |  |
| [Group=CTL] * [Depth=1040] | 0.006 | 0.799 | 0.371 |
| [Group=FSE] * [Depth=1040] | 0^a^ |  |  |
| [Group=CTL] * [Depth=1060] | 0.006 | 0.748 | 0.387 |
| [Group=FSE] * [Depth=1060] | 0^a^ |  |  |
| [Group=CTL] * [Depth=1080] | 0.007 | 1.085 | 0.298 |
| [Group=FSE] * [Depth=1080] | 0^a^ |  |  |
| [Group=CTL] * [Depth=1100] | 0.007 | 1.296 | 0.255 |
| [Group=FSE] * [Depth=1100] | 0^a^ |  |  |
| [Group=CTL] * [Depth=1120] | 0.007 | 1.325 | 0.250 |
| [Group=FSE] * [Depth=1120] | 0^a^ |  |  |
| [Group=CTL] * [Depth=1140] | 0.008 | 1.609 | 0.205 |
| [Group=FSE] * [Depth=1140] | 0^a^ |  |  |
| [Group=CTL] * [Depth=1180] | 0.008 | 1.740 | 0.187 |
| [Group=FSE] * [Depth=1180] | 0^a^ |  |  |
| [Group=CTL] * [Depth=1200] | 0.009 | 1.860 | 0.173 |
| [Group=FSE] * [Depth=1200] | 0^a^ |  |  |
| [Group=CTL] * [Depth=1220] | 0.007 | 1.317 | 0.251 |
| [Group=FSE] * [Depth=1220] | 0^a^ |  |  |
| [Group=CTL] * [Depth=1240] | 0.009 | 1.689 | 0.194 |
| [Group=FSE] * [Depth=1240] | 0^a^ |  |  |

Supplemental Table 4. Normalized TPS Power group x depth GEE analysis.

| Parameter | B | Hypothesis Test | |
| --- | --- | --- | --- |
|  |  | Wald Chi-Square | Sig. |
| [Group=CTL] * [Depth=0] | -0.010 | 5.240 | 0.022 |
| [Group=FSE] * [Depth=0] | 0^a^ |  |  |
| [Group=CTL] * [Depth=20] | -0.011 | 5.178 | 0.023 |
| [Group=FSE] * [Depth=20] | 0^a^ |  |  |
| [Group=CTL] * [Depth=40] | -0.010 | 4.144 | 0.042 |
| [Group=FSE] * [Depth=40] | 0^a^ |  |  |
| [Group=CTL] * [Depth=60] | -0.010 | 3.094 | 0.079 |
| [Group=FSE] * [Depth=60] | 0^a^ |  |  |
| [Group=CTL] * [Depth=80] | -0.010 | 2.771 | 0.096 |
| [Group=FSE] * [Depth=80] | 0^a^ |  |  |
| [Group=CTL] * [Depth=100] | -0.011 | 2.834 | 0.092 |
| [Group=FSE] * [Depth=100] | 0^a^ |  |  |
| [Group=CTL] * [Depth=120] | -0.001 | 0.046 | 0.831 |
| [Group=FSE] * [Depth=120] | 0^a^ |  |  |
| [Group=CTL] * [Depth=140] | -0.011 | 2.077 | 0.150 |
| [Group=FSE] * [Depth=140] | 0^a^ |  |  |
| [Group=CTL] * [Depth=160] | -0.010 | 1.300 | 0.254 |
| [Group=FSE] * [Depth=160] | 0^a^ |  |  |
| [Group=CTL] * [Depth=180] | -0.008 | 0.655 | 0.418 |
| [Group=FSE] * [Depth=180] | 0^a^ |  |  |
| [Group=CTL] * [Depth=200] | -0.006 | 0.310 | 0.578 |
| [Group=FSE] * [Depth=200] | 0^a^ |  |  |
| [Group=CTL] * [Depth=220] | -0.005 | 0.259 | 0.611 |
| [Group=FSE] * [Depth=220] | 0^a^ |  |  |
| [Group=CTL] * [Depth=240] | -0.007 | 0.562 | 0.453 |
| [Group=FSE] * [Depth=240] | 0^a^ |  |  |
| [Group=CTL] * [Depth=260] | -0.010 | 1.321 | 0.250 |
| [Group=FSE] * [Depth=260] | 0^a^ |  |  |
| [Group=CTL] * [Depth=280] | -0.014 | 3.742 | 0.053 |
| [Group=FSE] * [Depth=280] | 0^a^ |  |  |
| [Group=CTL] * [Depth=300] | -0.018 | 8.278 | 0.004 |
| [Group=FSE] * [Depth=300] | 0^a^ |  |  |
| [Group=CTL] * [Depth=320] | -0.020 | 12.714 | 0.000 |
| [Group=FSE] * [Depth=320] | 0^a^ |  |  |
| [Group=CTL] * [Depth=340] | -0.021 | 13.019 | 0.000 |
| [Group=FSE] * [Depth=340] | 0^a^ |  |  |
| [Group=CTL] * [Depth=360] | -0.021 | 9.531 | 0.002 |
| [Group=FSE] * [Depth=360] | 0^a^ |  |  |
| [Group=CTL] * [Depth=380] | -0.021 | 7.502 | 0.006 |
| [Group=FSE] * [Depth=380] | 0^a^ |  |  |
| [Group=CTL] * [Depth=400] | -0.022 | 5.820 | 0.016 |
| [Group=FSE] * [Depth=400] | 0^a^ |  |  |
| [Group=CTL] * [Depth=420] | -0.024 | 5.287 | 0.021 |
| [Group=FSE] * [Depth=420] | 0^a^ |  |  |
| [Group=CTL] * [Depth=440] | -0.001 | 0.040 | 0.842 |
| [Group=FSE] * [Depth=440] | 0^a^ |  |  |
| [Group=CTL] * [Depth=460] | -0.024 | 3.513 | 0.061 |
| [Group=FSE] * [Depth=460] | 0^a^ |  |  |
| [Group=CTL] * [Depth=480] | -0.024 | 2.996 | 0.083 |
| [Group=FSE] * [Depth=480] | 0^a^ |  |  |
| [Group=CTL] * [Depth=500] | -0.025 | 2.903 | 0.088 |
| [Group=FSE] * [Depth=500] | 0^a^ |  |  |
| [Group=CTL] * [Depth=520] | -0.027 | 2.927 | 0.087 |
| [Group=FSE] * [Depth=520] | 0^a^ |  |  |
| [Group=CTL] * [Depth=540] | -0.028 | 3.325 | 0.068 |
| [Group=FSE] * [Depth=540] | 0^a^ |  |  |
| [Group=CTL] * [Depth=560] | -0.030 | 3.830 | 0.050 |
| [Group=FSE] * [Depth=560] | 0^a^ |  |  |
| [Group=CTL] * [Depth=600] | -0.030 | 5.297 | 0.021 |
| [Group=FSE] * [Depth=600] | 0^a^ |  |  |
| [Group=CTL] * [Depth=620] | -0.030 | 6.271 | 0.012 |
| [Group=FSE] * [Depth=620] | 0^a^ |  |  |
| [Group=CTL] * [Depth=640] | -0.031 | 8.413 | 0.004 |
| [Group=FSE] * [Depth=640] | 0^a^ |  |  |
| [Group=CTL] * [Depth=660] | -0.032 | 10.175 | 0.001 |
| [Group=FSE] * [Depth=660] | 0^a^ |  |  |
| [Group=CTL] * [Depth=680] | -0.034 | 10.539 | 0.001 |
| [Group=FSE] * [Depth=680] | 0^a^ |  |  |
| [Group=CTL] * [Depth=700] | -0.032 | 8.190 | 0.004 |
| [Group=FSE] * [Depth=700] | 0^a^ |  |  |
| [Group=CTL] * [Depth=720] | -0.027 | 5.062 | 0.024 |
| [Group=FSE] * [Depth=720] | 0^a^ |  |  |
| [Group=CTL] * [Depth=740] | -0.022 | 2.330 | 0.127 |
| [Group=FSE] * [Depth=740] | 0^a^ |  |  |
| [Group=CTL] * [Depth=760] | -0.021 | 1.551 | 0.213 |
| [Group=FSE] * [Depth=760] | 0^a^ |  |  |
| [Group=CTL] * [Depth=780] | -0.025 | 1.875 | 0.171 |
| [Group=FSE] * [Depth=780] | 0^a^ |  |  |
| [Group=CTL] * [Depth=800] | -0.030 | 2.202 | 0.138 |
| [Group=FSE] * [Depth=800] | 0^a^ |  |  |
| [Group=CTL] * [Depth=820] | -0.031 | 1.872 | 0.171 |
| [Group=FSE] * [Depth=820] | 0^a^ |  |  |
| [Group=CTL] * [Depth=840] | -0.027 | 1.243 | 0.265 |
| [Group=FSE] * [Depth=840] | 0^a^ |  |  |
| [Group=CTL] * [Depth=860] | -0.021 | 0.805 | 0.370 |
| [Group=FSE] * [Depth=860] | 0^a^ |  |  |
| [Group=CTL] * [Depth=880] | -0.016 | 0.540 | 0.462 |
| [Group=FSE] * [Depth=880] | 0^a^ |  |  |
| [Group=CTL] * [Depth=900] | -0.014 | 0.533 | 0.466 |
| [Group=FSE] * [Depth=900] | 0^a^ |  |  |
| [Group=CTL] * [Depth=920] | -0.014 | 0.615 | 0.433 |
| [Group=FSE] * [Depth=920] | 0^a^ |  |  |
| [Group=CTL] * [Depth=940] | -0.012 | 0.498 | 0.480 |
| [Group=FSE] * [Depth=940] | 0^a^ |  |  |
| [Group=CTL] * [Depth=960] | -0.010 | 0.306 | 0.580 |
| [Group=FSE] * [Depth=960] | 0^a^ |  |  |
| [Group=CTL] * [Depth=1000] | -0.006 | 0.089 | 0.766 |
| [Group=FSE] * [Depth=1000] | 0^a^ |  |  |
| [Group=CTL] * [Depth=1020] | -0.005 | 0.055 | 0.815 |
| [Group=FSE] * [Depth=1020] | 0^a^ |  |  |
| [Group=CTL] * [Depth=1040] | -0.004 | 0.046 | 0.830 |
| [Group=FSE] * [Depth=1040] | 0^a^ |  |  |
| [Group=CTL] * [Depth=1060] | -0.004 | 0.048 | 0.826 |
| [Group=FSE] * [Depth=1060] | 0^a^ |  |  |
| [Group=CTL] * [Depth=1080] | -0.004 | 0.054 | 0.817 |
| [Group=FSE] * [Depth=1080] | 0^a^ |  |  |
| [Group=CTL] * [Depth=1100] | -0.005 | 0.073 | 0.787 |
| [Group=FSE] * [Depth=1100] | 0^a^ |  |  |
| [Group=CTL] * [Depth=1120] | -0.006 | 0.115 | 0.735 |
| [Group=FSE] * [Depth=1120] | 0^a^ |  |  |
| [Group=CTL] * [Depth=1140] | -0.006 | 0.125 | 0.724 |
| [Group=FSE] * [Depth=1140] | 0^a^ |  |  |
| [Group=CTL] * [Depth=1180] | -0.006 | 0.171 | 0.679 |
| [Group=FSE] * [Depth=1180] | 0^a^ |  |  |
| [Group=CTL] * [Depth=1200] | -0.006 | 0.218 | 0.641 |
| [Group=FSE] * [Depth=1200] | 0^a^ |  |  |
| [Group=CTL] * [Depth=1220] | -0.007 | 0.326 | 0.568 |
| [Group=FSE] * [Depth=1220] | 0^a^ |  |  |
| [Group=CTL] * [Depth=1240] | -0.008 | 0.512 | 0.474 |
| [Group=FSE] * [Depth=1240] | 0^a^ |  |  |

Supplemental Table 5. GEE analysis of slow gamma frequency, group x depth interaction.

| Parameter | B | Hypothesis Test | |
| --- | --- | --- | --- |
|  |  | Wald Chi-Square | Sig. |
| [Group=CTL] * [Depth=0] | 0.008 | 0.549 | 0.459 |
| [Group=FSE] * [Depth=0] | 0^a^ |  |  |
| [Group=CTL] * [Depth=20] | 0.008 | 0.583 | 0.445 |
| [Group=FSE] * [Depth=20] | 0^a^ |  |  |
| [Group=CTL] * [Depth=40] | 0.009 | 0.810 | 0.368 |
| [Group=FSE] * [Depth=40] | 0^a^ |  |  |
| [Group=CTL] * [Depth=60] | 0.008 | 0.686 | 0.407 |
| [Group=FSE] * [Depth=60] | 0^a^ |  |  |
| [Group=CTL] * [Depth=80] | 0.009 | 0.816 | 0.366 |
| [Group=FSE] * [Depth=80] | 0^a^ |  |  |
| [Group=CTL] * [Depth=100] | 0.010 | 1.265 | 0.261 |
| [Group=FSE] * [Depth=100] | 0^a^ |  |  |
| [Group=CTL] * [Depth=120] | 0.022 | 3.817 | 0.051 |
| [Group=FSE] * [Depth=120] | 0^a^ |  |  |
| [Group=CTL] * [Depth=140] | 0.008 | 1.564 | 0.211 |
| [Group=FSE] * [Depth=140] | 0^a^ |  |  |
| [Group=CTL] * [Depth=160] | 0.009 | 1.796 | 0.180 |
| [Group=FSE] * [Depth=160] | 0^a^ |  |  |
| [Group=CTL] * [Depth=180] | 0.006 | 0.778 | 0.378 |
| [Group=FSE] * [Depth=180] | 0^a^ |  |  |
| [Group=CTL] * [Depth=200] | 0.009 | 1.583 | 0.208 |
| [Group=FSE] * [Depth=200] | 0^a^ |  |  |
| [Group=CTL] * [Depth=220] | 0.012 | 3.358 | 0.067 |
| [Group=FSE] * [Depth=220] | 0^a^ |  |  |
| [Group=CTL] * [Depth=240] | 0.014 | 4.690 | 0.030 |
| [Group=FSE] * [Depth=240] | 0^a^ |  |  |
| [Group=CTL] * [Depth=260] | 0.015 | 6.884 | 0.009 |
| [Group=FSE] * [Depth=260] | 0^a^ |  |  |
| [Group=CTL] * [Depth=280] | 0.014 | 9.885 | 0.002 |
| [Group=FSE] * [Depth=280] | 0^a^ |  |  |
| [Group=CTL] * [Depth=300] | 0.016 | 11.085 | 0.001 |
| [Group=FSE] * [Depth=300] | 0^a^ |  |  |
| [Group=CTL] * [Depth=320] | 0.016 | 11.572 | 0.001 |
| [Group=FSE] * [Depth=320] | 0^a^ |  |  |
| [Group=CTL] * [Depth=340] | 0.017 | 10.553 | 0.001 |
| [Group=FSE] * [Depth=340] | 0^a^ |  |  |
| [Group=CTL] * [Depth=360] | 0.020 | 13.437 | 0.000 |
| [Group=FSE] * [Depth=360] | 0^a^ |  |  |
| [Group=CTL] * [Depth=380] | 0.020 | 12.531 | 0.000 |
| [Group=FSE] * [Depth=380] | 0^a^ |  |  |
| [Group=CTL] * [Depth=400] | 0.021 | 12.419 | 0.000 |
| [Group=FSE] * [Depth=400] | 0^a^ |  |  |
| [Group=CTL] * [Depth=420] | 0.020 | 11.800 | 0.001 |
| [Group=FSE] * [Depth=420] | 0^a^ |  |  |
| [Group=CTL] * [Depth=440] | 0.023 | 4.320 | 0.038 |
| [Group=FSE] * [Depth=440] | 0^a^ |  |  |
| [Group=CTL] * [Depth=460] | 0.021 | 16.794 | 0.000 |
| [Group=FSE] * [Depth=460] | 0^a^ |  |  |
| [Group=CTL] * [Depth=480] | 0.019 | 11.331 | 0.001 |
| [Group=FSE] * [Depth=480] | 0^a^ |  |  |
| [Group=CTL] * [Depth=500] | 0.019 | 13.046 | 0.000 |
| [Group=FSE] * [Depth=500] | 0^a^ |  |  |
| [Group=CTL] * [Depth=520] | 0.018 | 11.088 | 0.001 |
| [Group=FSE] * [Depth=520] | 0^a^ |  |  |
| [Group=CTL] * [Depth=540] | 0.020 | 12.329 | 0.000 |
| [Group=FSE] * [Depth=540] | 0^a^ |  |  |
| [Group=CTL] * [Depth=560] | 0.020 | 11.200 | 0.001 |
| [Group=FSE] * [Depth=560] | 0^a^ |  |  |
| [Group=CTL] * [Depth=600] | 0.019 | 10.532 | 0.001 |
| [Group=FSE] * [Depth=600] | 0^a^ |  |  |
| [Group=CTL] * [Depth=620] | 0.019 | 10.100 | 0.001 |
| [Group=FSE] * [Depth=620] | 0^a^ |  |  |
| [Group=CTL] * [Depth=640] | 0.018 | 13.022 | 0.000 |
| [Group=FSE] * [Depth=640] | 0^a^ |  |  |
| [Group=CTL] * [Depth=660] | 0.019 | 16.193 | 0.000 |
| [Group=FSE] * [Depth=660] | 0^a^ |  |  |
| [Group=CTL] * [Depth=680] | 0.019 | 16.939 | 0.000 |
| [Group=FSE] * [Depth=680] | 0^a^ |  |  |
| [Group=CTL] * [Depth=700] | 0.015 | 4.496 | 0.034 |
| [Group=FSE] * [Depth=700] | 0^a^ |  |  |
| [Group=CTL] * [Depth=720] | 0.016 | 4.061 | 0.044 |
| [Group=FSE] * [Depth=720] | 0^a^ |  |  |
| [Group=CTL] * [Depth=740] | 0.017 | 5.244 | 0.022 |
| [Group=FSE] * [Depth=740] | 0^a^ |  |  |
| [Group=CTL] * [Depth=760] | 0.019 | 5.422 | 0.020 |
| [Group=FSE] * [Depth=760] | 0^a^ |  |  |
| [Group=CTL] * [Depth=780] | 0.020 | 4.210 | 0.040 |
| [Group=FSE] * [Depth=780] | 0^a^ |  |  |
| [Group=CTL] * [Depth=800] | 0.021 | 4.646 | 0.031 |
| [Group=FSE] * [Depth=800] | 0^a^ |  |  |
| [Group=CTL] * [Depth=820] | 0.018 | 3.508 | 0.061 |
| [Group=FSE] * [Depth=820] | 0^a^ |  |  |
| [Group=CTL] * [Depth=840] | 0.016 | 2.696 | 0.101 |
| [Group=FSE] * [Depth=840] | 0^a^ |  |  |
| [Group=CTL] * [Depth=860] | 0.016 | 2.885 | 0.089 |
| [Group=FSE] * [Depth=860] | 0^a^ |  |  |
| [Group=CTL] * [Depth=880] | 0.015 | 2.650 | 0.104 |
| [Group=FSE] * [Depth=880] | 0^a^ |  |  |
| [Group=CTL] * [Depth=900] | 0.016 | 3.180 | 0.075 |
| [Group=FSE] * [Depth=900] | 0^a^ |  |  |
| [Group=CTL] * [Depth=920] | 0.018 | 3.349 | 0.067 |
| [Group=FSE] * [Depth=920] | 0^a^ |  |  |
| [Group=CTL] * [Depth=940] | 0.019 | 4.311 | 0.038 |
| [Group=FSE] * [Depth=940] | 0^a^ |  |  |
| [Group=CTL] * [Depth=960] | 0.020 | 5.192 | 0.023 |
| [Group=FSE] * [Depth=960] | 0^a^ |  |  |
| [Group=CTL] * [Depth=1000] | 0.021 | 6.652 | 0.010 |
| [Group=FSE] * [Depth=1000] | 0^a^ |  |  |
| [Group=CTL] * [Depth=1020] | 0.021 | 8.802 | 0.003 |
| [Group=FSE] * [Depth=1020] | 0^a^ |  |  |
| [Group=CTL] * [Depth=1040] | 0.019 | 7.949 | 0.005 |
| [Group=FSE] * [Depth=1040] | 0^a^ |  |  |
| [Group=CTL] * [Depth=1060] | 0.019 | 7.493 | 0.006 |
| [Group=FSE] * [Depth=1060] | 0^a^ |  |  |
| [Group=CTL] * [Depth=1080] | 0.019 | 8.394 | 0.004 |
| [Group=FSE] * [Depth=1080] | 0^a^ |  |  |
| [Group=CTL] * [Depth=1100] | 0.020 | 10.659 | 0.001 |
| [Group=FSE] * [Depth=1100] | 0^a^ |  |  |
| [Group=CTL] * [Depth=1120] | 0.020 | 10.513 | 0.001 |
| [Group=FSE] * [Depth=1120] | 0^a^ |  |  |
| [Group=CTL] * [Depth=1140] | 0.020 | 10.133 | 0.001 |
| [Group=FSE] * [Depth=1140] | 0^a^ |  |  |
| [Group=CTL] * [Depth=1180] | 0.019 | 10.127 | 0.001 |
| [Group=FSE] * [Depth=1180] | 0^a^ |  |  |
| [Group=CTL] * [Depth=1200] | 0.020 | 10.385 | 0.001 |
| [Group=FSE] * [Depth=1200] | 0^a^ |  |  |
| [Group=CTL] * [Depth=1220] | 0.020 | 10.189 | 0.001 |
| [Group=FSE] * [Depth=1220] | 0^a^ |  |  |
| [Group=CTL] * [Depth=1240] | 0.019 | 9.357 | 0.002 |
| [Group=FSE] * [Depth=1240] | 0^a^ |  |  |

**Coherence Tables**

Supplemental Table 6. CA1 phase coherence group x depth referenced to the SLM. “Parameters” values have been edited for length. Depth values are presented in µm.

|  | | | |
| --- | --- | --- | --- |
| Parameter | B | Hypothesis Test | |
|  |  | Wald Chi-Square | Sig. |
| [Group=CTL] * [Depth = 0] | 0.563 | 14.043 | 0.000 |
| [Group=FSE] * [Depth = 0] | 0^a^ |  |  |
| [Group=CTL] * [Depth = 20] | 0.563 | 14.043 | 0.000 |
| [Group=FSE] * [Depth = 20] | 0^a^ |  |  |
| [Group=CTL] * [Depth = 40] | 0.469 | 10.196 | 0.001 |
| [Group=FSE] * [Depth = 40] | 0^a^ |  |  |
| [Group=CTL] * [Depth = 60] | 0.450 | 9.492 | 0.002 |
| [Group=FSE] * [Depth = 60] | 0^a^ |  |  |
| [Group=CTL] * [Depth = 80] | 0.450 | 9.348 | 0.002 |
| [Group=FSE] * [Depth = 80] | 0^a^ |  |  |
| [Group=CTL] * [Depth = 100] | 0.390 | 7.040 | 0.008 |
| [Group=FSE] * [Depth = 100] | 0^a^ |  |  |
| [Group=CTL] * [Depth = 120] | 0.127 | 0.540 | 0.462 |
| [Group=FSE] * [Depth = 120] | 0^a^ |  |  |
| [Group=CTL] * [Depth = 140] | 0.387 | 6.793 | 0.009 |
| [Group=FSE] * [Depth = 140] | 0^a^ |  |  |
| [Group=CTL] * [Depth = 160] | 0.296 | 3.953 | 0.047 |
| [Group=FSE] * [Depth = 160] | 0^a^ |  |  |
| [Group=CTL] * [Depth = 180] | 0.271 | 3.005 | 0.083 |
| [Group=FSE] * [Depth = 180] | 0^a^ |  |  |
| [Group=CTL] * [Depth = 200] | 0.203 | 1.480 | 0.224 |
| [Group=FSE] * [Depth = 200] | 0^a^ |  |  |
| [Group=CTL] * [Depth = 220] | 0.095 | 0.298 | 0.585 |
| [Group=FSE] * [Depth = 220] | 0^a^ |  |  |
| [Group=CTL] * [Depth = 240] | 0.072 | 0.169 | 0.681 |
| [Group=FSE] * [Depth = 240] | 0^a^ |  |  |
| [Group=CTL] * [Depth = 260] | 0.156 | 0.875 | 0.350 |
| [Group=FSE] * [Depth = 260] | 0^a^ |  |  |
| [Group=CTL] * [Depth = 280] | 0.391 | 5.740 | 0.017 |
| [Group=FSE] * [Depth = 280] | 0^a^ |  |  |
| [Group=CTL] * [Depth = 300] | 0.497 | 8.371 | 0.004 |
| [Group=FSE] * [Depth = 300] | 0^a^ |  |  |
| [Group=CTL] * [Depth = 320] | 0.390 | 4.650 | 0.031 |
| [Group=FSE] * [Depth = 320] | 0^a^ |  |  |
| [Group=CTL] * [Depth = 340] | 0.358 | 4.256 | 0.039 |
| [Group=FSE] * [Depth = 340] | 0^a^ |  |  |
| [Group=CTL] * [Depth = 360] | 0.207 | 1.662 | 0.197 |
| [Group=FSE] * [Depth = 360] | 0^a^ |  |  |
| [Group=CTL] * [Depth = 380] | 0.206 | 2.189 | 0.139 |
| [Group=FSE] * [Depth = 380] | 0^a^ |  |  |
| [Group=CTL] * [Depth = 400] | 0.129 | 1.122 | 0.290 |
| [Group=FSE] * [Depth = 400] | 0^a^ |  |  |
| [Group=CTL] * [Depth = 420] | 0.138 | 1.845 | 0.174 |
| [Group=FSE] * [Depth = 420] | 0^a^ |  |  |
| [Group=CTL] * [Depth = 440] | 0.123 | 0.504 | 0.478 |
| [Group=FSE] * [Depth = 440] | 0^a^ |  |  |
| [Group=CTL] * [Depth = 460] | 0.116 | 2.156 | 0.142 |
| [Group=FSE] * [Depth = 460] | 0^a^ |  |  |
| [Group=CTL] * [Depth = 480] | 0.109 | 2.686 | 0.101 |
| [Group=FSE] * [Depth = 480] | 0^a^ |  |  |
| [Group=CTL] * [Depth = 500] | 0.101 | 3.585 | 0.058 |
| [Group=FSE] * [Depth = 500] | 0^a^ |  |  |
| [Group=CTL] * [Depth = 520] | 0.089 | 4.672 | 0.031 |
| [Group=FSE] * [Depth = 520] | 0^a^ |  |  |
| [Group=CTL] * [Depth = 540] | 0.078 | 6.330 | 0.012 |
| [Group=FSE] * [Depth = 540] | 0^a^ |  |  |
| [Group=CTL] * [Depth = 560] | 0.061 | 7.774 | 0.005 |
| [Group=FSE] * [Depth = 560] | 0^a^ |  |  |
| [Group=CTL] * [Depth = 580] | 0.043 | 9.882 | 0.002 |
| [Group=FSE] * [Depth = 580] | 0^a^ |  |  |
| [Group=CTL] * [Depth = 600] | 0.035 | 16.394 | 0.000 |
| [Group=FSE] * [Depth = 600] | 0^a^ |  |  |
| [Group=CTL] * [Depth = 620] | 0.005 | 1.764 | 0.184 |
| [Group=FSE] * [Depth = 620] | 0^a^ |  |  |
| [Group=CTL] * [Depth = 640] | 0.007 | 1.331 | 0.249 |
| [Group=FSE] * [Depth = 640] | 0^a^ |  |  |
| [Group=CTL] * [Depth = 660] | 0.005 | 0.882 | 0.348 |
| [Group=FSE] * [Depth = 660] | 0^a^ |  |  |
| [Group=CTL] * [Depth = 680] | 0.010 | 2.450 | 0.118 |
| [Group=FSE] * [Depth = 680] | 0^a^ |  |  |

Supplemental Table 7. GEE analysis of group x frequency phase coherence in SP (ref to SLM) of TPS from long window analysis. “Parameters” values have been edited for length. Frequency values are presented in Hz.

|  | | | |
| --- | --- | --- | --- |
| Parameter | B | Hypothesis Test | |
|  |  | Wald Chi-Square | Sig. |
| [Group=CTL ] * [Frequency=1.13932] | -0.433 | 6.543 | 0.011 |
| [Group=FSE ] * [Frequency=1.13932] | 0a |  |  |
| [Group=CTL ] * [Frequency=1.30208] | -0.412 | 7.122 | 0.008 |
| [Group=FSE ] * [Frequency=1.30208] | 0a |  |  |
| [Group=CTL ] * [Frequency=1.46484] | -0.413 | 6.139 | 0.013 |
| [Group=FSE ] * [Frequency=1.46484] | 0a |  |  |
| [Group=CTL ] * [Frequency=1.62760] | -0.251 | 1.758 | 0.185 |
| [Group=FSE ] * [Frequency=1.62760] | 0a |  |  |
| [Group=CTL ] * [Frequency=1.79036] | 0.190 | 0.914 | 0.339 |
| [Group=FSE ] * [Frequency=1.79036] | 0a |  |  |
| [Group=CTL ] * [Frequency=1.95313] | 0.831 | 12.823 | 0.000 |
| [Group=FSE ] * [Frequency=1.95313] | 0a |  |  |
| [Group=CTL ] * [Frequency=2.11589] | 0.994 | 18.136 | 0.000 |
| [Group=FSE ] * [Frequency=2.11589] | 0a |  |  |
| [Group=CTL ] * [Frequency=2.27865] | 0.674 | 10.646 | 0.001 |
| [Group=FSE ] * [Frequency=2.27865] | 0a |  |  |
| [Group=CTL ] * [Frequency=2.44141] | 0.572 | 10.737 | 0.001 |
| [Group=FSE ] * [Frequency=2.44141] | 0a |  |  |
| [Group=CTL ] * [Frequency=2.60417] | 0.498 | 12.246 | 0.000 |
| [Group=FSE ] * [Frequency=2.60417] | 0a |  |  |
| [Group=CTL ] * [Frequency=2.76693] | 0.356 | 11.003 | 0.001 |
| [Group=FSE ] * [Frequency=2.76693] | 0a |  |  |
| [Group=CTL ] * [Frequency=2.92969] | 0.281 | 9.155 | 0.002 |
| [Group=FSE ] * [Frequency=2.92969] | 0a |  |  |
| [Group=CTL ] * [Frequency=3.09245] | 0.227 | 6.152 | 0.013 |
| [Group=FSE ] * [Frequency=3.09245] | 0a |  |  |
| [Group=CTL ] * [Frequency=3.25521] | 0.198 | 4.143 | 0.042 |
| [Group=FSE ] * [Frequency=3.25521] | 0a |  |  |
| [Group=CTL ] * [Frequency=3.41797] | 0.229 | 3.988 | 0.046 |
| [Group=FSE ] * [Frequency=3.41797] | 0a |  |  |
| [Group=CTL ] * [Frequency=3.58073] | 0.295 | 4.625 | 0.032 |
| [Group=FSE ] * [Frequency=3.58073] | 0a |  |  |
| [Group=CTL ] * [Frequency=3.74349] | 0.372 | 5.566 | 0.018 |
| [Group=FSE ] * [Frequency=3.74349] | 0a |  |  |
| [Group=CTL ] * [Frequency=3.90625] | 0.392 | 5.151 | 0.023 |
| [Group=FSE ] * [Frequency=3.90625] | 0a |  |  |
| [Group=CTL ] * [Frequency=4.06901] | 0.247 | 2.252 | 0.133 |
| [Group=FSE ] * [Frequency=4.06901] | 0a |  |  |
| [Group=CTL ] * [Frequency=4.23177] | 0.131 | 0.667 | 0.414 |
| [Group=FSE ] * [Frequency=4.23177] | 0a |  |  |
| [Group=CTL ] * [Frequency=4.39453] | 0.010 | 0.003 | 0.957 |
| [Group=FSE ] * [Frequency=4.39453] | 0a |  |  |
| [Group=CTL ] * [Frequency=4.55729] | -0.173 | 0.791 | 0.374 |
| [Group=FSE ] * [Frequency=4.55729] | 0a |  |  |
| [Group=CTL ] * [Frequency=4.72005] | -0.189 | 0.755 | 0.385 |
| [Group=FSE ] * [Frequency=4.72005] | 0a |  |  |
| [Group=CTL ] * [Frequency=4.88281] | 0.449 | 4.479 | 0.034 |
| [Group=FSE ] * [Frequency=4.88281] | 0a |  |  |
| [Group=CTL ] * [Frequency=5.04557] | 0.756 | 14.548 | 0.000 |
| [Group=FSE ] * [Frequency=5.04557] | 0a |  |  |

Supplemental Table 8. Analysis of group x frequency phase coherence in SR (ref to SLM) of TPS from long window analysis. “Parameters” values have been edited for length. Frequency values are presented in Hz.

|  | | | |
| --- | --- | --- | --- |
| Parameter | B | Hypothesis Test | |
|  |  | Wald Chi-Square | Sig. |
| [Group=CTL ] * [Frequency=1.13932] | 0.000 | 0.000 | 0.998 |
| [Group=FSE ] * [Frequency=1.13932] | 0a |  |  |
| [Group=CTL ] * [Frequency=1.30208] | -0.005 | 0.005 | 0.946 |
| [Group=FSE ] * [Frequency=1.30208] | 0a |  |  |
| [Group=CTL ] * [Frequency=1.46484] | -0.082 | 0.675 | 0.411 |
| [Group=FSE ] * [Frequency=1.46484] | 0a |  |  |
| [Group=CTL ] * [Frequency=1.62760] | -0.100 | 0.624 | 0.429 |
| [Group=FSE ] * [Frequency=1.62760] | 0a |  |  |
| [Group=CTL ] * [Frequency=1.79036] | 0.006 | 0.002 | 0.966 |
| [Group=FSE ] * [Frequency=1.79036] | 0a |  |  |
| [Group=CTL ] * [Frequency=1.95313] | 0.189 | 1.706 | 0.192 |
| [Group=FSE ] * [Frequency=1.95313] | 0a |  |  |
| [Group=CTL ] * [Frequency=2.11589] | 0.352 | 4.466 | 0.035 |
| [Group=FSE ] * [Frequency=2.11589] | 0a |  |  |
| [Group=CTL ] * [Frequency=2.27865] | 0.438 | 5.422 | 0.020 |
| [Group=FSE ] * [Frequency=2.27865] | 0a |  |  |
| [Group=CTL ] * [Frequency=2.44141] | 0.500 | 7.004 | 0.008 |
| [Group=FSE ] * [Frequency=2.44141] | 0a |  |  |
| [Group=CTL ] * [Frequency=2.60417] | 0.519 | 7.770 | 0.005 |
| [Group=FSE ] * [Frequency=2.60417] | 0a |  |  |
| [Group=CTL ] * [Frequency=2.76693] | 0.428 | 5.527 | 0.019 |
| [Group=FSE ] * [Frequency=2.76693] | 0a |  |  |
| [Group=CTL ] * [Frequency=2.92969] | 0.356 | 4.456 | 0.035 |
| [Group=FSE ] * [Frequency=2.92969] | 0a |  |  |
| [Group=CTL ] * [Frequency=3.09245] | 0.326 | 3.948 | 0.047 |
| [Group=FSE ] * [Frequency=3.09245] | 0a |  |  |
| [Group=CTL ] * [Frequency=3.25521] | 0.345 | 4.307 | 0.038 |
| [Group=FSE ] * [Frequency=3.25521] | 0a |  |  |
| [Group=CTL ] * [Frequency=3.41797] | 0.370 | 4.464 | 0.035 |
| [Group=FSE ] * [Frequency=3.41797] | 0a |  |  |
| [Group=CTL ] * [Frequency=3.58073] | 0.353 | 3.205 | 0.073 |
| [Group=FSE ] * [Frequency=3.58073] | 0a |  |  |
| [Group=CTL ] * [Frequency=3.74349] | 0.306 | 2.195 | 0.138 |
| [Group=FSE ] * [Frequency=3.74349] | 0a |  |  |
| [Group=CTL ] * [Frequency=3.90625] | 0.235 | 1.417 | 0.234 |
| [Group=FSE ] * [Frequency=3.90625] | 0a |  |  |
| [Group=CTL ] * [Frequency=4.06901] | 0.168 | 0.711 | 0.399 |
| [Group=FSE ] * [Frequency=4.06901] | 0a |  |  |
| [Group=CTL ] * [Frequency=4.23177] | 0.206 | 0.946 | 0.331 |
| [Group=FSE ] * [Frequency=4.23177] | 0a |  |  |
| [Group=CTL ] * [Frequency=4.39453] | 0.116 | 0.311 | 0.577 |
| [Group=FSE ] * [Frequency=4.39453] | 0a |  |  |
| [Group=CTL ] * [Frequency=4.55729] | -0.138 | 0.468 | 0.494 |
| [Group=FSE ] * [Frequency=4.55729] | 0a |  |  |
| [Group=CTL ] * [Frequency=4.72005] | -0.431 | 4.397 | 0.036 |
| [Group=FSE ] * [Frequency=4.72005] | 0a |  |  |
| [Group=CTL ] * [Frequency=4.88281] | -0.421 | 4.488 | 0.034 |
| [Group=FSE ] * [Frequency=4.88281] | 0a |  |  |
| [Group=CTL ] * [Frequency=5.04557] | -0.148 | 0.565 | 0.452 |
| [Group=FSE ] * [Frequency=5.04557] | 0a |  |  |

Supplemental Table 9. Group x frequency statistical analysis of phase coherence in SP (ref to SLM) of higher frequencies during TPS from short window analysis. “Parameters” values have been edited for length. Frequency values are presented in Hz.

| Parameter | B | Hypothesis Test | |
| --- | --- | --- | --- |
|  |  | Wald Chi-Square | Sig. |
| [Group=CTL ] * [Frequency=6.347656] | 0.741 | 16.904 | 0.000 |
| [Group=FSE ] * [Frequency=6.347656] | 0^a^ |  |  |
| [Group=CTL ] * [Frequency=8.300781] | 0.700 | 10.945 | 0.001 |
| [Group=FSE ] * [Frequency=8.300781] | 0^a^ |  |  |
| [Group=CTL ] * [Frequency=10.253906] | 1.048 | 24.395 | 0.000 |
| [Group=FSE ] * [Frequency=10.253906] | 0^a^ |  |  |
| [Group=CTL ] * [Frequency=12.207031] | 0.637 | 10.309 | 0.001 |
| [Group=FSE ] * [Frequency=12.207031] | 0^a^ |  |  |
| [Group=CTL ] * [Frequency=14.160156] | 0.855 | 16.287 | 0.000 |
| [Group=FSE ] * [Frequency=14.160156] | 0^a^ |  |  |
| [Group=CTL ] * [Frequency=16.113281] | 0.546 | 8.278 | 0.004 |
| [Group=FSE ] * [Frequency=16.113281] | 0^a^ |  |  |
| [Group=CTL ] * [Frequency=18.066406] | 0.060 | 0.085 | 0.770 |
| [Group=FSE ] * [Frequency=18.066406] | 0^a^ |  |  |
| [Group=CTL ] * [Frequency=20.019531] | 0.076 | 0.199 | 0.655 |
| [Group=FSE ] * [Frequency=20.019531] | 0^a^ |  |  |
| [Group=CTL ] * [Frequency=22.460938] | 0.064 | 0.109 | 0.742 |
| [Group=FSE ] * [Frequency=22.460938] | 0^a^ |  |  |
| [Group=CTL ] * [Frequency=24.414063] | -0.245 | 1.514 | 0.219 |
| [Group=FSE ] * [Frequency=24.414063] | 0^a^ |  |  |
| [Group=CTL ] * [Frequency=26.367188] | -0.084 | 0.204 | 0.652 |
| [Group=FSE ] * [Frequency=26.367188] | 0^a^ |  |  |
| [Group=CTL ] * [Frequency=28.320313] | 0.085 | 0.192 | 0.661 |
| [Group=FSE ] * [Frequency=28.320313] | 0^a^ |  |  |
| [Group=CTL ] * [Frequency=30.273438] | -0.334 | 2.999 | 0.083 |
| [Group=FSE ] * [Frequency=30.273438] | 0^a^ |  |  |
| [Group=CTL ] * [Frequency=32.226563] | -0.183 | 1.044 | 0.307 |
| [Group=FSE ] * [Frequency=32.226563] | 0^a^ |  |  |
| [Group=CTL ] * [Frequency=34.179688] | -0.184 | 0.876 | 0.349 |
| [Group=FSE ] * [Frequency=34.179688] | 0^a^ |  |  |
| [Group=CTL ] * [Frequency=36.132813] | -0.361 | 4.257 | 0.039 |
| [Group=FSE ] * [Frequency=36.132813] | 0^a^ |  |  |
| [Group=CTL ] * [Frequency=38.085938] | -0.608 | 9.306 | 0.002 |
| [Group=FSE ] * [Frequency=38.085938] | 0^a^ |  |  |
| [Group=CTL ] * [Frequency=40.039063] | -0.251 | 2.065 | 0.151 |
| [Group=FSE ] * [Frequency=40.039063] | 0^a^ |  |  |
| [Group=CTL ] * [Frequency=42.480469] | -0.631 | 10.877 | 0.001 |
| [Group=FSE ] * [Frequency=42.480469] | 0^a^ |  |  |
| [Group=CTL ] * [Frequency=44.433594] | -0.539 | 9.585 | 0.002 |
| [Group=FSE ] * [Frequency=44.433594] | 0^a^ |  |  |
| [Group=CTL ] * [Frequency=46.386719] | -0.619 | 11.227 | 0.001 |
| [Group=FSE ] * [Frequency=46.386719] | 0^a^ |  |  |
| [Group=CTL ] * [Frequency=48.339844] | -0.419 | 5.170 | 0.023 |
| [Group=FSE ] * [Frequency=48.339844] | 0^a^ |  |  |
| [Group=CTL ] * [Frequency=50.292969] | -0.379 | 4.045 | 0.044 |
| [Group=FSE ] * [Frequency=50.292969] | 0^a^ |  |  |
| [Group=CTL ] * [Frequency=52.246094] | -0.561 | 10.597 | 0.001 |
| [Group=FSE ] * [Frequency=52.246094] | 0^a^ |  |  |
| [Group=CTL ] * [Frequency=54.199219] | -0.572 | 10.052 | 0.002 |
| [Group=FSE ] * [Frequency=54.199219] | 0^a^ |  |  |
| [Group=CTL ] * [Frequency=56.152344] | -0.770 | 21.544 | 0.000 |
| [Group=FSE ] * [Frequency=56.152344] | 0^a^ |  |  |
| [Group=CTL ] * [Frequency=58.105469] | -0.918 | 26.019 | 0.000 |
| [Group=FSE ] * [Frequency=58.105469] | 0^a^ |  |  |
| [Group=CTL ] * [Frequency=60.058594] | -0.025 | 0.037 | 0.848 |
| [Group=FSE ] * [Frequency=60.058594] | 0^a^ |  |  |
| [Group=CTL ] * [Frequency=62.011719] | -0.663 | 11.367 | 0.001 |
| [Group=FSE ] * [Frequency=62.011719] | 0^a^ |  |  |
| [Group=CTL ] * [Frequency=64.453125] | -0.224 | 1.561 | 0.211 |
| [Group=FSE ] * [Frequency=64.453125] | 0^a^ |  |  |
| [Group=CTL ] * [Frequency=66.406250] | -0.320 | 2.904 | 0.088 |
| [Group=FSE ] * [Frequency=66.406250] | 0^a^ |  |  |
| [Group=CTL ] * [Frequency=68.359375] | -0.426 | 4.852 | 0.028 |
| [Group=FSE ] * [Frequency=68.359375] | 0^a^ |  |  |
| [Group=CTL ] * [Frequency=70.312500] | -0.598 | 8.486 | 0.004 |
| [Group=FSE ] * [Frequency=70.312500] | 0^a^ |  |  |
| [Group=CTL ] * [Frequency=72.265625] | -0.678 | 12.292 | 0.000 |
| [Group=FSE ] * [Frequency=72.265625] | 0^a^ |  |  |
| [Group=CTL ] * [Frequency=74.218750] | -0.416 | 5.086 | 0.024 |
| [Group=FSE ] * [Frequency=74.218750] | 0^a^ |  |  |
| [Group=CTL ] * [Frequency=76.171875] | -0.592 | 9.859 | 0.002 |
| [Group=FSE ] * [Frequency=76.171875] | 0^a^ |  |  |
| [Group=CTL ] * [Frequency=78.125000] | -0.650 | 11.961 | 0.001 |
| [Group=FSE ] * [Frequency=78.125000] | 0^a^ |  |  |
| [Group=CTL ] * [Frequency=80.078125] | -0.715 | 13.101 | 0.000 |
| [Group=FSE ] * [Frequency=80.078125] | 0^a^ |  |  |
| [Group=CTL ] * [Frequency=82.031250] | -0.828 | 22.233 | 0.000 |
| [Group=FSE ] * [Frequency=82.031250] | 0^a^ |  |  |
| [Group=CTL ] * [Frequency=84.472656] | -0.444 | 4.534 | 0.033 |
| [Group=FSE ] * [Frequency=84.472656] | 0^a^ |  |  |
| [Group=CTL ] * [Frequency=86.425781] | -0.420 | 5.384 | 0.020 |
| [Group=FSE ] * [Frequency=86.425781] | 0^a^ |  |  |
| [Group=CTL ] * [Frequency=88.378906] | -0.344 | 3.942 | 0.047 |
| [Group=FSE ] * [Frequency=88.378906] | 0^a^ |  |  |
| [Group=CTL ] * [Frequency=90.332031] | -0.488 | 6.016 | 0.014 |
| [Group=FSE ] * [Frequency=90.332031] | 0^a^ |  |  |
| [Group=CTL ] * [Frequency=92.285156] | -0.702 | 13.263 | 0.000 |
| [Group=FSE ] * [Frequency=92.285156] | 0^a^ |  |  |
| [Group=CTL ] * [Frequency=94.238281] | -0.472 | 5.908 | 0.015 |
| [Group=FSE ] * [Frequency=94.238281] | 0^a^ |  |  |
| [Group=CTL ] * [Frequency=96.191406] | 0.095 | 0.283 | 0.595 |
| [Group=FSE ] * [Frequency=96.191406] | 0^a^ |  |  |
| [Group=CTL ] * [Frequency=98.144531] | -0.095 | 0.254 | 0.614 |
| [Group=FSE ] * [Frequency=98.144531] | 0^a^ |  |  |

Supplemental Table 10. Group x frequency statistical analysis of phase coherence in SR (ref to SLM) of higher frequencies during TPS from short window analysis. “Parameters” values have been edited for length. Frequency values are presented in Hz.

| Parameter | B | Hypothesis Test | | |
| --- | --- | --- | --- | --- |
|  |  | Wald Chi-Square | Sig. |  |
| [Group=CTL ] * [Frequency=6.347656] | 0.283 | 2.976 | 0.084 |  |
| [Group=FSE ] * [Frequency=6.347656] | 0^a^ |  |  |  |
| [Group=CTL ] * [Frequency=8.300781] | -0.061 | 0.226 | 0.634 |  |
| [Group=FSE ] * [Frequency=8.300781] | 0^a^ |  |  |  |
| [Group=CTL ] * [Frequency=10.253906] | 0.061 | 0.389 | 0.533 |  |
| [Group=FSE ] * [Frequency=10.253906] | 0^a^ |  |  |  |
| [Group=CTL ] * [Frequency=12.207031] | -0.031 | 0.095 | 0.758 |  |
| [Group=FSE ] * [Frequency=12.207031] | 0^a^ |  |  |  |
| [Group=CTL ] * [Frequency=14.160156] | 0.004 | 0.001 | 0.972 |  |
| [Group=FSE ] * [Frequency=14.160156] | 0^a^ |  |  |  |
| [Group=CTL ] * [Frequency=16.113281] | -0.005 | 0.003 | 0.958 |  |
| [Group=FSE ] * [Frequency=16.113281] | 0^a^ |  |  |  |
| [Group=CTL ] * [Frequency=18.066406] | -0.046 | 0.174 | 0.677 |  |
| [Group=FSE ] * [Frequency=18.066406] | 0^a^ |  |  |  |
| [Group=CTL ] * [Frequency=20.019531] | -0.015 | 0.030 | 0.863 |  |
| [Group=FSE ] * [Frequency=20.019531] | 0^a^ |  |  |  |
| [Group=CTL ] * [Frequency=22.460938] | -0.115 | 1.055 | 0.304 |  |
| [Group=FSE ] * [Frequency=22.460938] | 0^a^ |  |  |  |
| [Group=CTL ] * [Frequency=24.414063] | -0.156 | 1.575 | 0.209 |  |
| [Group=FSE ] * [Frequency=24.414063] | 0^a^ |  |  |  |
| [Group=CTL ] * [Frequency=26.367188] | -0.032 | 0.105 | 0.746 |  |
| [Group=FSE ] * [Frequency=26.367188] | 0^a^ |  |  |  |
| [Group=CTL ] * [Frequency=28.320313] | -0.116 | 1.094 | 0.296 |  |
| [Group=FSE ] * [Frequency=28.320313] | 0^a^ |  |  |  |
| [Group=CTL ] * [Frequency=30.273438] | -0.134 | 1.101 | 0.294 |  |
| [Group=FSE ] * [Frequency=30.273438] | 0^a^ |  |  |  |
| [Group=CTL ] * [Frequency=32.226563] | -0.044 | 0.169 | 0.681 |  |
| [Group=FSE ] * [Frequency=32.226563] | 0^a^ |  |  |  |
| [Group=CTL ] * [Frequency=34.179688] | -0.027 | 0.065 | 0.798 |  |
| [Group=FSE ] * [Frequency=34.179688] | 0^a^ |  |  |  |
| [Group=CTL ] * [Frequency=36.132813] | -0.053 | 0.357 | 0.550 |  |
| [Group=FSE ] * [Frequency=36.132813] | 0^a^ |  |  |  |
| [Group=CTL ] * [Frequency=38.085938] | -0.134 | 1.545 | 0.214 |  |
| [Group=FSE ] * [Frequency=38.085938] | 0^a^ |  |  |  |
| [Group=CTL ] * [Frequency=40.039063] | -0.064 | 0.855 | 0.355 |  |
| [Group=FSE ] * [Frequency=40.039063] | 0^a^ |  |  |  |
| [Group=CTL ] * [Frequency=42.480469] | -0.193 | 3.283 | 0.070 |  |
| [Group=FSE ] * [Frequency=42.480469] | 0^a^ |  |  |  |
| [Group=CTL ] * [Frequency=44.433594] | -0.109 | 1.496 | 0.221 |  |
| [Group=FSE ] * [Frequency=44.433594] | 0^a^ |  |  |  |
| [Group=CTL ] * [Frequency=48.339844] | -0.073 | 1.022 | 0.312 |  |
| [Group=FSE ] * [Frequency=48.339844] | 0^a^ |  |  |  |
| [Group=CTL ] * [Frequency=50.292969] | 0.015 | 0.034 | 0.853 |  |
| [Group=FSE ] * [Frequency=50.292969] | 0^a^ |  |  |  |
| [Group=CTL ] * [Frequency=52.246094] | -0.160 | 4.379 | 0.036 |  |
| [Group=FSE ] * [Frequency=52.246094] | 0^a^ |  |  |  |
| [Group=CTL ] * [Frequency=54.199219] | -0.079 | 1.060 | 0.303 |  |
| [Group=FSE ] * [Frequency=54.199219] | 0^a^ |  |  |  |
| [Group=CTL ] * [Frequency=56.152344] | -0.110 | 2.800 | 0.094 |  |
| [Group=FSE ] * [Frequency=56.152344] | 0^a^ |  |  |  |
| [Group=CTL ] * [Frequency=58.105469] | -0.165 | 6.706 | 0.010 |  |
| [Group=FSE ] * [Frequency=58.105469] | 0^a^ |  |  |  |
| [Group=CTL ] * [Frequency=60.058594] | -0.037 | 0.477 | 0.490 |  |
| [Group=FSE ] * [Frequency=60.058594] | 0^a^ |  |  |  |
| [Group=CTL ] * [Frequency=62.011719] | -0.243 | 9.026 | 0.003 |  |
| [Group=FSE ] * [Frequency=62.011719] | 0^a^ |  |  |  |
| [Group=CTL ] * [Frequency=64.453125] | -0.025 | 0.145 | 0.704 |  |
| [Group=FSE ] * [Frequency=64.453125] | 0^a^ |  |  |  |
| [Group=CTL ] * [Frequency=66.406250] | -0.042 | 0.455 | 0.500 |  |
| [Group=FSE ] * [Frequency=66.406250] | 0^a^ |  |  |  |
| [Group=CTL ] * [Frequency=68.359375] | -0.139 | 2.970 | 0.085 |  |
| [Group=FSE ] * [Frequency=68.359375] | 0^a^ |  |  |  |
| [Group=CTL ] * [Frequency=70.312500] | -0.129 | 2.446 | 0.118 |  |
| [Group=FSE ] * [Frequency=70.312500] | 0^a^ |  |  |  |
| [Group=CTL ] * [Frequency=72.265625] | -0.170 | 4.881 | 0.027 |  |
| [Group=FSE ] * [Frequency=72.265625] | 0^a^ |  |  |  |
| [Group=CTL ] * [Frequency=74.218750] | -0.057 | 1.011 | 0.315 |  |
| [Group=FSE ] * [Frequency=74.218750] | 0^a^ |  |  |  |
| [Group=CTL ] * [Frequency=76.171875] | -0.126 | 3.198 | 0.074 |  |
| [Group=FSE ] * [Frequency=76.171875] | 0^a^ |  |  |  |
| [Group=CTL ] * [Frequency=78.125000] | -0.175 | 7.527 | 0.006 |  |
| [Group=FSE ] * [Frequency=78.125000] | 0^a^ |  |  |  |
| [Group=CTL ] * [Frequency=80.078125] | -0.218 | 8.743 | 0.003 |  |
| [Group=FSE ] * [Frequency=80.078125] | 0^a^ |  |  |  |
| [Group=CTL ] * [Frequency=84.472656] | -0.116 | 2.921 | 0.087 |  |
| [Group=FSE ] * [Frequency=84.472656] | 0^a^ |  |  |  |
| [Group=CTL ] * [Frequency=86.425781] | -0.366 | 28.200 | 0.000 |  |
| [Group=FSE ] * [Frequency=86.425781] | 0^a^ |  |  |  |
| [Group=CTL ] * [Frequency=88.378906] | -0.199 | 9.064 | 0.003 |  |
| [Group=FSE ] * [Frequency=88.378906] | 0^a^ |  |  |  |
| [Group=CTL ] * [Frequency=90.332031] | -0.229 | 8.785 | 0.003 |  |
| [Group=FSE ] * [Frequency=90.332031] | 0^a^ |  |  |  |
| [Group=CTL ] * [Frequency=92.285156] | -0.223 | 12.272 | 0.000 |  |
| [Group=FSE ] * [Frequency=92.285156] | 0^a^ |  |  |  |
| [Group=CTL ] * [Frequency=94.238281] | -0.144 | 4.042 | 0.044 |  |
| [Group=FSE ] * [Frequency=94.238281] | 0^a^ |  |  |  |
| [Group=CTL ] * [Frequency=96.191406] | -0.069 | 1.021 | 0.312 |  |
| [Group=FSE ] * [Frequency=96.191406] | 0^a^ |  |  |  |
| [Group=CTL ] * [Frequency=98.144531] | -0.131 | 3.120 | 0.077 |  |
| [Group=FSE ] * [Frequency=98.144531] | 0^a^ |  |  |  |

Supplemental Table 11. Dentate gyrus phase coherence group x depth referenced to OML. “Parameters” values have been edited for length. Depth values are presented in µm.

|  | | | | |
| --- | --- | --- | --- | --- |
| Parameter | B | Hypothesis Test | | |
|  |  | Wald Chi-Square | Sig. |  |
| [Group=CTL ] * [Depth = 680] | -0.001 | 0.020 | 0.887 |  |
| [Group=FSE ] * [Depth = 680] | 0^a^ |  |  |  |
| [Group=CTL ] * [Depth = 700] | 1.203E-06 | 0.000 | 1.000 |  |
| [Group=FSE ] * [Depth = 700] | 0^a^ |  |  |  |
| [Group=CTL ] * [Depth = 720] | 0.001 | 0.048 | 0.827 |  |
| [Group=FSE ] * [Depth = 720] | 0^a^ |  |  |  |
| [Group=CTL ] * [Depth = 740] | 0.001 | 0.239 | 0.625 |  |
| [Group=FSE ] * [Depth = 740] | 0^a^ |  |  |  |
| [Group=CTL ] * [Depth = 760] | 0.000 | 0.169 | 0.681 |  |
| [Group=FSE ] * [Depth = 760] | 0^a^ |  |  |  |
| [Group=CTL ] * [Depth = 780] | 0.000 |  |  |  |
| [Group=FSE ] * [Depth = 780] | 0^a^ |  |  |  |
| [Group=CTL ] * [Depth = 800] | 0.000 | 0.122 | 0.727 |  |
| [Group=FSE ] * [Depth = 800] | 0^a^ |  |  |  |
| [Group=CTL ] * [Depth = 820] | 0.000 | 0.145 | 0.704 |  |
| [Group=FSE ] * [Depth = 820] | 0^a^ |  |  |  |
| [Group=CTL ] * [Depth = 840] | 0.002 | 0.823 | 0.364 |  |
| [Group=FSE ] * [Depth = 840] | 0^a^ |  |  |  |
| [Group=CTL ] * [Depth = 860] | 0.006 | 2.073 | 0.150 |  |
| [Group=FSE ] * [Depth = 860] | 0^a^ |  |  |  |
| [Group=CTL ] * [Depth = 880] | 0.011 | 3.276 | 0.703 |  |
| [Group=FSE ] * [Depth = 880] | 0^a^ |  |  |  |
| [Group=CTL ] * [Depth = 900] | 0.018 | 3.871 | 0.491 |  |
| [Group=FSE ] * [Depth = 900] | 0^a^ |  |  |  |
| [Group=CTL ] * [Depth = 920] | 0.028 | 4.860 | 0.275 |  |
| [Group=FSE ] * [Depth = 920] | 0^a^ |  |  |  |
| [Group=CTL ] * [Depth = 940] | 0.043 | 5.894 | 0.152 |  |
| [Group=FSE ] * [Depth = 940] | 0^a^ |  |  |  |
| [Group=CTL ] * [Depth = 960] | 0.060 | 6.493 | 0.108 |  |
| [Group=FSE ] * [Depth = 960] | 0^a^ |  |  |  |
| [Group=CTL ] * [Depth = 980] | 0.078 | 6.975 | 0.008 |  |
| [Group=FSE ] * [Depth = 980] | 0^a^ |  |  |  |
| [Group=CTL ] * [Depth = 1000] | 0.101 | 7.889 | 0.005 |  |
| [Group=FSE ] * [Depth = 1000] | 0^a^ |  |  |  |
| [Group=CTL ] * [Depth = 1020] | 0.124 | 8.667 | 0.003 |  |
| [Group=FSE ] * [Depth = 1020] | 0^a^ |  |  |  |
| [Group=CTL ] * [Depth = 1040] | 0.144 | 8.997 | 0.003 |  |
| [Group=FSE ] * [Depth = 1040] | 0^a^ |  |  |  |
| [Group=CTL ] * [Depth = 1060] | 0.161 | 9.017 | 0.003 |  |
| [Group=FSE ] * [Depth = 1060] | 0^a^ |  |  |  |
| [Group=CTL ] * [Depth = 1080] | 0.180 | 8.873 | 0.003 |  |
| [Group=FSE ] * [Depth = 1080] | 0^a^ |  |  |  |
| [Group=CTL ] * [Depth = 1100] | 0.194 | 8.497 | 0.004 |  |
| [Group=FSE ] * [Depth = 1100] | 0^a^ |  |  |  |
| [Group=CTL ] * [Depth = 1120] | 0.210 | 8.404 | 0.004 |  |
| [Group=FSE ] * [Depth = 1120] | 0^a^ |  |  |  |
| [Group=CTL ] * [Depth = 1140] | 0.225 | 8.295 | 0.004 |  |
| [Group=FSE ] * [Depth = 1140] | 0^a^ |  |  |  |
| [Group=CTL ] * [Depth = 1180] | 0.247 | 7.967 | 0.005 |  |
| [Group=FSE ] * [Depth = 1180] | 0^a^ |  |  |  |
| [Group=CTL ] * [Depth = 1200] | 0.255 | 7.764 | 0.005 |  |
| [Group=FSE ] * [Depth = 1200] | 0^a^ |  |  |  |
| [Group=CTL ] * [Depth = 1220] | 0.263 | 7.700 | 0.006 |  |
| [Group=FSE ] * [Depth = 1220] | 0^a^ |  |  |  |
| [Group=CTL ] * [Depth = 1240] | 0.276 | 7.943 | 0.005 |  |
| [Group=FSE ] * [Depth = 1240] | 0^a^ |  |  |  |

Supplemental Table 12. Analysis of TPS group x frequency phase coherence in MML (ref to OML) from long window analysis. “Parameters” values have been edited for length. Frequency values are presented in Hz.

|  | | | |
| --- | --- | --- | --- |
| Parameter | B | Hypothesis Test | |
|  |  | Wald Chi-Square | Sig. |
| [Group=CTL ] * [Frequency=1.13932] | 0.035 | 3.755 | 0.053 |
| [Group=FSE ] * [Frequency=1.13932] | 0a |  |  |
| [Group=CTL ] * [Frequency=1.30208] | 0.048 | 7.149 | 0.007 |
| [Group=FSE ] * [Frequency=1.30208] | 0a |  |  |
| [Group=CTL ] * [Frequency=1.46484] | 0.059 | 8.577 | 0.003 |
| [Group=FSE ] * [Frequency=1.46484] | 0a |  |  |
| [Group=CTL ] * [Frequency=1.62760] | 0.055 | 6.753 | 0.009 |
| [Group=FSE ] * [Frequency=1.62760] | 0a |  |  |
| [Group=CTL ] * [Frequency=1.79036] | 0.032 | 3.317 | 0.069 |
| [Group=FSE ] * [Frequency=1.79036] | 0a |  |  |
| [Group=CTL ] * [Frequency=1.95313] | 0.005 | 0.117 | 0.732 |
| [Group=FSE ] * [Frequency=1.95313] | 0a |  |  |
| [Group=CTL ] * [Frequency=2.11589] | -0.003 | 0.074 | 0.785 |
| [Group=FSE ] * [Frequency=2.11589] | 0a |  |  |
| [Group=CTL ] * [Frequency=2.27865] | 0.003 | 0.065 | 0.799 |
| [Group=FSE ] * [Frequency=2.27865] | 0a |  |  |
| [Group=CTL ] * [Frequency=2.44141] | 0.015 | 1.318 | 0.251 |
| [Group=FSE ] * [Frequency=2.44141] | 0a |  |  |
| [Group=CTL ] * [Frequency=2.60417] | 0.027 | 3.812 | 0.051 |
| [Group=FSE ] * [Frequency=2.60417] | 0a |  |  |
| [Group=CTL ] * [Frequency=2.76693] | 0.026 | 5.807 | 0.016 |
| [Group=FSE ] * [Frequency=2.76693] | 0a |  |  |
| [Group=CTL ] * [Frequency=2.92969] | 0.018 | 4.125 | 0.042 |
| [Group=FSE ] * [Frequency=2.92969] | 0a |  |  |
| [Group=CTL ] * [Frequency=3.09245] | 0.013 | 1.900 | 0.168 |
| [Group=FSE ] * [Frequency=3.09245] | 0a |  |  |
| [Group=CTL ] * [Frequency=3.25521] | 0.010 | 1.025 | 0.311 |
| [Group=FSE ] * [Frequency=3.25521] | 0a |  |  |
| [Group=CTL ] * [Frequency=3.41797] | 0.011 | 1.241 | 0.265 |
| [Group=FSE ] * [Frequency=3.41797] | 0a |  |  |
| [Group=CTL ] * [Frequency=3.58073] | 0.019 | 2.721 | 0.099 |
| [Group=FSE ] * [Frequency=3.58073] | 0a |  |  |
| [Group=CTL ] * [Frequency=3.74349] | 0.031 | 3.009 | 0.083 |
| [Group=FSE ] * [Frequency=3.74349] | 0a |  |  |
| [Group=CTL ] * [Frequency=3.90625] | 0.037 | 2.672 | 0.102 |
| [Group=FSE ] * [Frequency=3.90625] | 0a |  |  |
| [Group=CTL ] * [Frequency=4.06901] | 0.022 | 1.594 | 0.207 |
| [Group=FSE ] * [Frequency=4.06901] | 0a |  |  |
| [Group=CTL ] * [Frequency=4.23177] | 0.005 | 0.125 | 0.724 |
| [Group=FSE ] * [Frequency=4.23177] | 0a |  |  |
| [Group=CTL ] * [Frequency=4.39453] | -0.004 | 0.061 | 0.806 |
| [Group=FSE ] * [Frequency=4.39453] | 0a |  |  |
| [Group=CTL ] * [Frequency=4.55729] | -0.007 | 0.094 | 0.760 |
| [Group=FSE ] * [Frequency=4.55729] | 0a |  |  |
| [Group=CTL ] * [Frequency=4.72005] | -0.008 | 0.069 | 0.793 |
| [Group=FSE ] * [Frequency=4.72005] | 0a |  |  |
| [Group=CTL ] * [Frequency=4.88281] | -0.006 | 0.022 | 0.881 |
| [Group=FSE ] * [Frequency=4.88281] | 0a |  |  |
| [Group=CTL ] * [Frequency=5.04557] | 0.009 | 0.045 | 0.832 |
| [Group=FSE ] * [Frequency=5.04557] | 0a |  |  |

Supplemental Table 13. TPS Group x frequency phase coherence in DGC (ref to OML) from long window analysis. “Parameters” values have been edited for length. Frequency values are presented in Hz.

|  | | | |
| --- | --- | --- | --- |
| Parameter | B | Hypothesis Test | |
|  |  | Wald Chi-Square | Sig. |
| [Group=CTL ] * [Frequency=1.13932] | 0.073 | 2.227 | 0.136 |
| [Group=FSE ] * [Frequency=1.13932] | 0a |  |  |
| [Group=CTL ] * [Frequency=1.30208] | 0.165 | 16.609 | 0.000 |
| [Group=FSE ] * [Frequency=1.30208] | 0a |  |  |
| [Group=CTL ] * [Frequency=1.46484] | 0.210 | 26.434 | 0.000 |
| [Group=FSE ] * [Frequency=1.46484] | 0a |  |  |
| [Group=CTL ] * [Frequency=1.62760] | 0.197 | 21.564 | 0.000 |
| [Group=FSE ] * [Frequency=1.62760] | 0a |  |  |
| [Group=CTL ] * [Frequency=1.79036] | 0.126 | 10.658 | 0.001 |
| [Group=FSE ] * [Frequency=1.79036] | 0a |  |  |
| [Group=CTL ] * [Frequency=1.95313] | 0.014 | 0.190 | 0.663 |
| [Group=FSE ] * [Frequency=1.95313] | 0a |  |  |
| [Group=CTL ] * [Frequency=2.11589] | -0.020 | 0.526 | 0.468 |
| [Group=FSE ] * [Frequency=2.11589] | 0a |  |  |
| [Group=CTL ] * [Frequency=2.27865] | 0.028 | 0.678 | 0.410 |
| [Group=FSE ] * [Frequency=2.27865] | 0a |  |  |
| [Group=CTL ] * [Frequency=2.44141] | 0.098 | 3.636 | 0.057 |
| [Group=FSE ] * [Frequency=2.44141] | 0a |  |  |
| [Group=CTL ] * [Frequency=2.60417] | 0.171 | 7.980 | 0.005 |
| [Group=FSE ] * [Frequency=2.60417] | 0a |  |  |
| [Group=CTL ] * [Frequency=2.76693] | 0.193 | 13.543 | 0.000 |
| [Group=FSE ] * [Frequency=2.76693] | 0a |  |  |
| [Group=CTL ] * [Frequency=2.92969] | 0.147 | 20.272 | 0.000 |
| [Group=FSE ] * [Frequency=2.92969] | 0a |  |  |
| [Group=CTL ] * [Frequency=3.09245] | 0.099 | 13.459 | 0.000 |
| [Group=FSE ] * [Frequency=3.09245] | 0a |  |  |
| [Group=CTL ] * [Frequency=3.25521] | 0.077 | 8.907 | 0.003 |
| [Group=FSE ] * [Frequency=3.25521] | 0a |  |  |
| [Group=CTL ] * [Frequency=3.41797] | 0.087 | 11.066 | 0.001 |
| [Group=FSE ] * [Frequency=3.41797] | 0a |  |  |
| [Group=CTL ] * [Frequency=3.58073] | 0.149 | 13.129 | 0.000 |
| [Group=FSE ] * [Frequency=3.58073] | 0a |  |  |
| [Group=CTL ] * [Frequency=3.74349] | 0.228 | 8.986 | 0.003 |
| [Group=FSE ] * [Frequency=3.74349] | 0a |  |  |
| [Group=CTL ] * [Frequency=3.90625] | 0.246 | 5.782 | 0.016 |
| [Group=FSE ] * [Frequency=3.90625] | 0a |  |  |
| [Group=CTL ] * [Frequency=4.06901] | 0.156 | 3.651 | 0.056 |
| [Group=FSE ] * [Frequency=4.06901] | 0a |  |  |
| [Group=CTL ] * [Frequency=4.23177] | 0.011 | 0.048 | 0.827 |
| [Group=FSE ] * [Frequency=4.23177] | 0a |  |  |
| [Group=CTL ] * [Frequency=4.39453] | -0.068 | 1.359 | 0.244 |
| [Group=FSE ] * [Frequency=4.39453] | 0a |  |  |
| [Group=CTL ] * [Frequency=4.55729] | -0.074 | 0.723 | 0.395 |
| [Group=FSE ] * [Frequency=4.55729] | 0a |  |  |
| [Group=CTL ] * [Frequency=4.72005] | -0.046 | 0.174 | 0.677 |
| [Group=FSE ] * [Frequency=4.72005] | 0a |  |  |
| [Group=CTL ] * [Frequency=4.88281] | 0.033 | 0.091 | 0.763 |
| [Group=FSE ] * [Frequency=4.88281] | 0a |  |  |
| [Group=CTL ] * [Frequency=5.04557] | 0.102 | 1.060 | 0.303 |
| [Group=FSE ] * [Frequency=5.04557] | 0a |  |  |

Supplemental Table 14. Group x frequency phase coherence in DGC (ref to OML) of higher frequencies during TPS from short window analysis. “Parameters” values have been edited for length. Frequency values are presented in Hz.

|  | | | |
| --- | --- | --- | --- |
| Parameter | B | Hypothesis Test | |
|  |  | Wald Chi-Square | Sig. |
| [Group=CTL ] * [Frequency=6.347656] | -0.106 | 0.636 | 0.425 |
| [Group=FSE ] * [Frequency=6.347656] | 0a |  |  |
| [Group=CTL ] * [Frequency=8.300781] | 0.182 | 0.810 | 0.368 |
| [Group=FSE ] * [Frequency=8.300781] | 0a |  |  |
| [Group=CTL ] * [Frequency=10.253906] | -0.146 | 0.382 | 0.536 |
| [Group=FSE ] * [Frequency=10.253906] | 0a |  |  |
| [Group=CTL ] * [Frequency=12.207031] | -0.230 | 0.902 | 0.342 |
| [Group=FSE ] * [Frequency=12.207031] | 0a |  |  |
| [Group=CTL ] * [Frequency=14.160156] | -0.220 | 0.782 | 0.377 |
| [Group=FSE ] * [Frequency=14.160156] | 0a |  |  |
| [Group=CTL ] * [Frequency=16.113281] | -0.243 | 0.923 | 0.337 |
| [Group=FSE ] * [Frequency=16.113281] | 0a |  |  |
| [Group=CTL ] * [Frequency=18.066406] | -0.331 | 1.918 | 0.166 |
| [Group=FSE ] * [Frequency=18.066406] | 0a |  |  |
| [Group=CTL ] * [Frequency=20.019531] | -0.482 | 3.957 | 0.047 |
| [Group=FSE ] * [Frequency=20.019531] | 0a |  |  |
| [Group=CTL ] * [Frequency=22.460938] | -0.286 | 1.719 | 0.190 |
| [Group=FSE ] * [Frequency=22.460938] | 0a |  |  |
| [Group=CTL ] * [Frequency=24.414063] | -0.395 | 2.751 | 0.097 |
| [Group=FSE ] * [Frequency=24.414063] | 0a |  |  |
| [Group=CTL ] * [Frequency=26.367188] | -0.351 | 2.600 | 0.107 |
| [Group=FSE ] * [Frequency=26.367188] | 0a |  |  |
| [Group=CTL ] * [Frequency=28.320313] | -0.405 | 3.532 | 0.060 |
| [Group=FSE ] * [Frequency=28.320313] | 0a |  |  |
| [Group=CTL ] * [Frequency=30.273438] | -0.419 | 3.764 | 0.052 |
| [Group=FSE ] * [Frequency=30.273438] | 0a |  |  |
| [Group=CTL ] * [Frequency=32.226563] | -0.342 | 2.688 | 0.101 |
| [Group=FSE ] * [Frequency=32.226563] | 0a |  |  |
| [Group=CTL ] * [Frequency=34.179688] | -0.455 | 4.197 | 0.041 |
| [Group=FSE ] * [Frequency=34.179688] | 0a |  |  |
| [Group=CTL ] * [Frequency=36.132813] | -0.606 | 8.177 | 0.004 |
| [Group=FSE ] * [Frequency=36.132813] | 0a |  |  |
| [Group=CTL ] * [Frequency=38.085938] | -0.523 | 6.279 | 0.012 |
| [Group=FSE ] * [Frequency=38.085938] | 0a |  |  |
| [Group=CTL ] * [Frequency=40.039063] | -0.563 | 7.546 | 0.006 |
| [Group=FSE ] * [Frequency=40.039063] | 0a |  |  |
| [Group=CTL ] * [Frequency=42.480469] | -0.465 | 6.350 | 0.012 |
| [Group=FSE ] * [Frequency=42.480469] | 0a |  |  |
| [Group=CTL ] * [Frequency=44.433594] | -0.711 | 11.117 | 0.001 |
| [Group=FSE ] * [Frequency=44.433594] | 0a |  |  |
| [Group=CTL ] * [Frequency=46.386719] | -0.648 | 11.399 | 0.001 |
| [Group=FSE ] * [Frequency=46.386719] | 0a |  |  |
| [Group=CTL ] * [Frequency=48.339844] | -0.814 | 14.167 | 0.000 |
| [Group=FSE ] * [Frequency=48.339844] | 0a |  |  |
| [Group=CTL ] * [Frequency=50.292969] | -0.617 | 8.803 | 0.003 |
| [Group=FSE ] * [Frequency=50.292969] | 0a |  |  |
| [Group=CTL ] * [Frequency=52.246094] | -0.719 | 11.983 | 0.001 |
| [Group=FSE ] * [Frequency=52.246094] | 0a |  |  |
| [Group=CTL ] * [Frequency=54.199219] | -0.740 | 12.827 | 0.000 |
| [Group=FSE ] * [Frequency=54.199219] | 0a |  |  |
| [Group=CTL ] * [Frequency=56.152344] | -0.582 | 8.479 | 0.004 |
| [Group=FSE ] * [Frequency=56.152344] | 0a |  |  |
| [Group=CTL ] * [Frequency=58.105469] | -0.634 | 9.864 | 0.002 |
| [Group=FSE ] * [Frequency=58.105469] | 0a |  |  |
| [Group=CTL ] * [Frequency=60.058594] | -0.703 | 12.076 | 0.001 |
| [Group=FSE ] * [Frequency=60.058594] | 0a |  |  |
| [Group=CTL ] * [Frequency=62.011719] | -0.687 | 11.363 | 0.001 |
| [Group=FSE ] * [Frequency=62.011719] | 0a |  |  |
| [Group=CTL ] * [Frequency=64.453125] | -0.673 | 10.681 | 0.001 |
| [Group=FSE ] * [Frequency=64.453125] | 0a |  |  |
| [Group=CTL ] * [Frequency=66.406250] | -0.386 | 3.756 | 0.053 |
| [Group=FSE ] * [Frequency=66.406250] | 0a |  |  |
| [Group=CTL ] * [Frequency=68.359375] | -0.496 | 5.762 | 0.016 |
| [Group=FSE ] * [Frequency=68.359375] | 0a |  |  |
| [Group=CTL ] * [Frequency=70.312500] | -0.631 | 9.861 | 0.002 |
| [Group=FSE ] * [Frequency=70.312500] | 0a |  |  |
| [Group=CTL ] * [Frequency=72.265625] | -0.505 | 7.171 | 0.007 |
| [Group=FSE ] * [Frequency=72.265625] | 0a |  |  |
| [Group=CTL ] * [Frequency=74.218750] | -0.627 | 8.665 | 0.003 |
| [Group=FSE ] * [Frequency=74.218750] | 0a |  |  |
| [Group=CTL ] * [Frequency=76.171875] | -0.586 | 10.153 | 0.001 |
| [Group=FSE ] * [Frequency=76.171875] | 0a |  |  |
| [Group=CTL ] * [Frequency=78.125000] | -0.559 | 7.772 | 0.005 |
| [Group=FSE ] * [Frequency=78.125000] | 0a |  |  |
| [Group=CTL ] * [Frequency=80.078125] | -0.576 | 8.747 | 0.003 |
| [Group=FSE ] * [Frequency=80.078125] | 0a |  |  |
| [Group=CTL ] * [Frequency=82.031250] | -0.655 | 9.500 | 0.002 |
| [Group=FSE ] * [Frequency=82.031250] | 0a |  |  |
| [Group=CTL ] * [Frequency=84.472656] | -0.370 | 3.820 | 0.051 |
| [Group=FSE ] * [Frequency=84.472656] | 0a |  |  |
| [Group=CTL ] * [Frequency=86.425781] | -0.461 | 5.881 | 0.015 |
| [Group=FSE ] * [Frequency=86.425781] | 0a |  |  |
| [Group=CTL ] * [Frequency=88.378906] | -0.422 | 4.465 | 0.035 |
| [Group=FSE ] * [Frequency=88.378906] | 0a |  |  |
| [Group=CTL ] * [Frequency=90.332031] | -0.505 | 7.021 | 0.008 |
| [Group=FSE ] * [Frequency=90.332031] | 0a |  |  |
| [Group=CTL ] * [Frequency=92.285156] | -0.378 | 4.055 | 0.044 |
| [Group=FSE ] * [Frequency=92.285156] | 0a |  |  |
| [Group=CTL ] * [Frequency=94.238281] | -0.367 | 3.860 | 0.049 |
| [Group=FSE ] * [Frequency=94.238281] | 0a |  |  |
| [Group=CTL ] * [Frequency=96.191406] | -0.435 | 5.920 | 0.015 |
| [Group=FSE ] * [Frequency=96.191406] | 0a |  |  |
| [Group=CTL ] * [Frequency=98.144531] | -0.522 | 7.891 | 0.005 |
| [Group=FSE ] * [Frequency=98.144531] | 0a |  |  |

**Cross Frequency Coupling**

Supplemental Table 15: GEE for MI statistics for CFC between TPS and slow gamma range frequencies; FSE SP layer used as a comparator (0a indicates statistical comparator at SP).

| Parameter | Hypothesis Test | |  |
| --- | --- | --- | --- |
|  | Wald Chi-Square | Sig. |  |
| [Group=CTL] * [Location=SP] | 0.158 | 0.691 |  |
| [Group=CTL] * [Location=SR] | 9.104 | 0.003 |  |
| [Group=CTL] * [Location=SLM] | 4.579 | 0.032 |  |
| [Group=CTL] * [Location=OML] | 1.294 | 0.255 |  |
| [Group=CTL] * [Location=MML] | 0.011 | 0.917 |  |
| [Group=CTL] * [Location=DGC] | 1.936 | 0.164 |  |
| [Group=FSE] * [Location=SR] | 0.425 | 0.514 |  |
| [Group=FSE] * [Location=SLM] | 0.171 | 0.680 |  |
| [Group=FSE] * [Location=OML] | 0.343 | 0.558 |  |
| [Group=FSE] * [Location=MML] | 0.447 | 0.504 |  |
| [Group=FSE] * [Location=DGC] | 1.093 | 0.296 |  |
| [Group=FSE] * [Location=SP] | 0a |  |  |

Supplemental Table 16: GEE for MI statistics for CFC between TPS and medium gamma range frequencies; FSE SP layer used as a comparator (0a indicates statistical comparator at SP).

| Parameter | Hypothesis Test | |  |
| --- | --- | --- | --- |
|  | Wald Chi-Square | Sig. |  |
| [Group=CTL] * [Location=SP] | 2.259 | 0.133 |  |
| [Group=CTL] * [Location=SR] | 5.056 | 0.025 |  |
| [Group=CTL] * [Location=SLM] | 1.479 | 0.224 |  |
| [Group=CTL] * [Location=OML] | 0.688 | 0.407 |  |
| [Group=CTL] * [Location=MML] | 0.234 | 0.629 |  |
| [Group=CTL] * [Location=DGC] | 4.632 | 0.031 |  |
| [Group=FSE] * [Location=SR] | 0.105 | 0.746 |  |
| [Group=FSE] * [Location=SLM] | 0.424 | 0.515 |  |
| [Group=FSE] * [Location=OML] | 0.292 | 0.589 |  |
| [Group=FSE] * [Location=MML] | 0.000 | 0.982 |  |
| [Group=FSE] * [Location=DGC] | 2.225 | 0.136 |  |
| [Group=FSE] * [Location=SP] | 0a |  |  |

Supplemental Table 17: GEE for MI statistics for CFC between TPS and frequencies in the fast oscillation range; FSE SP layer used as a comparator (0a indicates statistical comparator at SP).

| Parameter | Hypothesis Test | |  |
| --- | --- | --- | --- |
|  | Wald Chi-Square | Sig. |  |
| [Group=CTL] * [Location=SP] | 4.28 | 0.039 |  |
| [Group=CTL] * [Location=SR] | 1.56 | 0.212 |  |
| [Group=CTL] * [Location=SLM] | 11.90 | 0.001 |  |
| [Group=CTL] * [Location=OML] | 31.68 | 0.000 |  |
| [Group=CTL] * [Location=MML] | 23.27 | 0.000 |  |
| [Group=CTL] * [Location=DGC] | 1.85 | 0.174 |  |
| [Group=FSE] * [Location=SR] | 0.54 | 0.464 |  |
| [Group=FSE] * [Location=SLM] | 0.84 | 0.361 |  |
| [Group=FSE] * [Location=OML] | 14.20 | 0.000 |  |
| [Group=FSE] * [Location=MML] | 7.55 | 0.006 |  |
| [Group=FSE] * [Location=DGC] | 1.78 | 0.182 |  |
| [Group=FSE] * [Location=SP] | 0a |  |  |

Supplemental Table 18: GEE for MI statistics and Group x Frequency interactions at SP. The CTL peak at 135 Hz is used as a comparator (0a indicates statistical comparator).

|  | | |
| --- | --- | --- |
| Parameter | Hypothesis Test | |
|  | Wald Chi-Square | Sig. |
| [Group=FSE] * [Frequency=205.00] | 6.835 | 0.0090 |
| [Group=FSE] * [Frequency=200.00] | 6.711 | 0.0100 |
| [Group=FSE] * [Frequency=195.00] | 7.284 | 0.0070 |
| [Group=FSE] * [Frequency=190.00] | 8.02 | 0.0050 |
| [Group=FSE] * [Frequency=185.00] | 8.6 | 0.0030 |
| [Group=FSE] * [Frequency=180.00] | 9.674 | 0.0020 |
| [Group=FSE] * [Frequency=175.00] | 10.22 | 0.0010 |
| [Group=FSE] * [Frequency=170.00] | 11.165 | 0.0010 |
| [Group=FSE] * [Frequency=165.00] | 11.227 | 0.0010 |
| [Group=FSE] * [Frequency=160.00] | 11.001 | 0.0010 |
| [Group=FSE] * [Frequency=155.00] | 11.014 | 0.0010 |
| [Group=FSE] * [Frequency=150.00] | 10.167 | 0.0010 |
| [Group=FSE] * [Frequency=145.00] | 9.238 | 0.0020 |
| [Group=FSE] * [Frequency=140.00] | 7.745 | 0.0050 |
| [Group=FSE] * [Frequency=135.00] | 6.591 | 0.0100 |
| [Group=FSE] * [Frequency=130.00] | 6.41 | 0.0110 |
| [Group=FSE] * [Frequency=125.00] | 7.415 | 0.0060 |
| [Group=FSE] * [Frequency=120.00] | 9.584 | 0.0020 |
| [Group=FSE] * [Frequency=115.00] | 12.614 | 0.0000 |
| [Group=FSE] * [Frequency=110.00] | 15.55 | 0.0000 |
| [Group=FSE] * [Frequency=105.00] | 18.665 | 0.0000 |
| [Group=FSE] * [Frequency=100.00] | 23.111 | 0.0000 |
| [Group=FSE] * [Frequency=95.00] | 26.865 | 0.0000 |
| [Group=FSE] * [Frequency=90.00] | 22.636 | 0.0000 |
| [Group=FSE] * [Frequency=85.00] | 16.783 | 0.0000 |
| [Group=FSE] * [Frequency=80.00] | 13.099 | 0.0000 |
| [Group=FSE] * [Frequency=75.00] | 13.178 | 0.0000 |
| [Group=FSE] * [Frequency=70.00] | 16.603 | 0.0000 |
| [Group=FSE] * [Frequency=65.00] | 22.612 | 0.0000 |
| [Group=FSE] * [Frequency=60.00] | 26.964 | 0.0000 |
| [Group=FSE] * [Frequency=55.00] | 27.167 | 0.0000 |
| [Group=FSE] * [Frequency=50.00] | 22.455 | 0.0000 |
| [Group=FSE] * [Frequency=45.00] | 12.745 | 0.0000 |
| [Group=FSE] * [Frequency=40.00] | 9.168 | 0.0020 |
| [Group=FSE] * [Frequency=35.00] | 7.288 | 0.0070 |
| [Group=FSE] * [Frequency=30.00] | 9.07 | 0.0030 |
| [Group=FSE] * [Frequency=25.00] | 3.943 | 0.0470 |
| [Group=CTL] * [Frequency=205.00] | 4.393 | 0.0360 |
| [Group=CTL] * [Frequency=200.00] | 3.96 | 0.0470 |
| [Group=CTL] * [Frequency=195.00] | 4.679 | 0.0310 |
| [Group=CTL] * [Frequency=190.00] | 6.178 | 0.0130 |
| [Group=CTL] * [Frequency=185.00] | 6.783 | 0.0090 |
| [Group=CTL] * [Frequency=180.00] | 8.479 | 0.0040 |
| [Group=CTL] * [Frequency=175.00] | 8.049 | 0.0050 |
| [Group=CTL] * [Frequency=170.00] | 8.513 | 0.0040 |
| [Group=CTL] * [Frequency=165.00] | 7.351 | 0.0070 |
| [Group=CTL] * [Frequency=160.00] | 6.122 | 0.0130 |
| [Group=CTL] * [Frequency=155.00] | 4.253 | 0.0390 |
| [Group=CTL] * [Frequency=150.00] | 1.714 | 0.1900 |
| [Group=CTL] * [Frequency=145.00] | 0.368 | 0.5440 |
| [Group=CTL] * [Frequency=140.00] | 0.007 | 0.9320 |
| [Group=CTL] * [Frequency=130.00] | 0.17 | 0.6800 |
| [Group=CTL] * [Frequency=125.00] | 0.455 | 0.5000 |
| [Group=CTL] * [Frequency=120.00] | 0.789 | 0.3740 |
| [Group=CTL] * [Frequency=115.00] | 1.175 | 0.2780 |
| [Group=CTL] * [Frequency=110.00] | 1.697 | 0.1930 |
| [Group=CTL] * [Frequency=105.00] | 4.141 | 0.0420 |
| [Group=CTL] * [Frequency=100.00] | 7.881 | 0.0050 |
| [Group=CTL] * [Frequency=95.00] | 29.315 | 0.0000 |
| [Group=CTL] * [Frequency=90.00] | 79.601 | 0.0000 |
| [Group=CTL] * [Frequency=85.00] | 92.359 | 0.0000 |
| [Group=CTL] * [Frequency=80.00] | 139.339 | 0.0000 |
| [Group=CTL] * [Frequency=75.00] | 185.811 | 0.0000 |
| [Group=CTL] * [Frequency=70.00] | 99.008 | 0.0000 |
| [Group=CTL] * [Frequency=65.00] | 60.265 | 0.0000 |
| [Group=CTL] * [Frequency=60.00] | 50.477 | 0.0000 |
| [Group=CTL] * [Frequency=55.00] | 51.055 | 0.0000 |
| [Group=CTL] * [Frequency=50.00] | 53.322 | 0.0000 |
| [Group=CTL] * [Frequency=45.00] | 33.836 | 0.0000 |
| [Group=CTL] * [Frequency=40.00] | 43.647 | 0.0000 |
| [Group=CTL] * [Frequency=35.00] | 15.619 | 0.0000 |
| [Group=CTL] * [Frequency=30.00] | 37.157 | 0.0000 |
| [Group=CTL] * [Frequency=25.00] | 10.299 | 0.0010 |
| [Group=CTL] * [Frequency=135.00] | 0a |  |

Supplemental Table 19: GEE for MI statistics and Group x Frequency interactions at SR. The CTL peak at 45 Hz is used as a comparator (0a indicates statistical comparator).

|  | | |
| --- | --- | --- |
| Parameter | Hypothesis Test | |
|  | Wald Chi-Square | Sig. |
| [Group=FSE] * [Frequency=205.00] | 10.908 | 0.0010 |
| [Group=FSE] * [Frequency=200.00] | 9.977 | 0.0020 |
| [Group=FSE] * [Frequency=195.00] | 10.463 | 0.0010 |
| [Group=FSE] * [Frequency=190.00] | 11.975 | 0.0010 |
| [Group=FSE] * [Frequency=185.00] | 11.78 | 0.0010 |
| [Group=FSE] * [Frequency=180.00] | 13.131 | 0.0000 |
| [Group=FSE] * [Frequency=175.00] | 12.032 | 0.0010 |
| [Group=FSE] * [Frequency=170.00] | 12.25 | 0.0000 |
| [Group=FSE] * [Frequency=165.00] | 10.756 | 0.0010 |
| [Group=FSE] * [Frequency=160.00] | 10.047 | 0.0020 |
| [Group=FSE] * [Frequency=155.00] | 9.728 | 0.0020 |
| [Group=FSE] * [Frequency=150.00] | 7.919 | 0.0050 |
| [Group=FSE] * [Frequency=145.00] | 7.156 | 0.0070 |
| [Group=FSE] * [Frequency=140.00] | 6.497 | 0.0110 |
| [Group=FSE] * [Frequency=135.00] | 6.013 | 0.0140 |
| [Group=FSE] * [Frequency=130.00] | 5.858 | 0.0160 |
| [Group=FSE] * [Frequency=125.00] | 6.201 | 0.0130 |
| [Group=FSE] * [Frequency=120.00] | 7.358 | 0.0070 |
| [Group=FSE] * [Frequency=115.00] | 9.372 | 0.0020 |
| [Group=FSE] * [Frequency=110.00] | 12.182 | 0.0000 |
| [Group=FSE] * [Frequency=105.00] | 16.76 | 0.0000 |
| [Group=FSE] * [Frequency=100.00] | 19.398 | 0.0000 |
| [Group=FSE] * [Frequency=95.00] | 21.527 | 0.0000 |
| [Group=FSE] * [Frequency=90.00] | 21.749 | 0.0000 |
| [Group=FSE] * [Frequency=85.00] | 22.367 | 0.0000 |
| [Group=FSE] * [Frequency=80.00] | 24.34 | 0.0000 |
| [Group=FSE] * [Frequency=75.00] | 25.964 | 0.0000 |
| [Group=FSE] * [Frequency=70.00] | 27.042 | 0.0000 |
| [Group=FSE] * [Frequency=65.00] | 26.184 | 0.0000 |
| [Group=FSE] * [Frequency=60.00] | 23.028 | 0.0000 |
| [Group=FSE] * [Frequency=55.00] | 18.451 | 0.0000 |
| [Group=FSE] * [Frequency=50.00] | 17.286 | 0.0000 |
| [Group=FSE] * [Frequency=45.00] | 17.089 | 0.0000 |
| [Group=FSE] * [Frequency=40.00] | 21.555 | 0.0000 |
| [Group=FSE] * [Frequency=35.00] | 27.523 | 0.0000 |
| [Group=FSE] * [Frequency=30.00] | 33.282 | 0.0000 |
| [Group=FSE] * [Frequency=25.00] | 42.64 | 0.0000 |
| [Group=CTL] * [Frequency=205.00] | 67.084 | 0.0000 |
| [Group=CTL] * [Frequency=200.00] | 39.377 | 0.0000 |
| [Group=CTL] * [Frequency=195.00] | 36.764 | 0.0000 |
| [Group=CTL] * [Frequency=190.00] | 40.657 | 0.0000 |
| [Group=CTL] * [Frequency=185.00] | 34.509 | 0.0000 |
| [Group=CTL] * [Frequency=180.00] | 42.311 | 0.0000 |
| [Group=CTL] * [Frequency=175.00] | 34.631 | 0.0000 |
| [Group=CTL] * [Frequency=170.00] | 42.198 | 0.0000 |
| [Group=CTL] * [Frequency=165.00] | 37.672 | 0.0000 |
| [Group=CTL] * [Frequency=160.00] | 44.41 | 0.0000 |
| [Group=CTL] * [Frequency=155.00] | 69.386 | 0.0000 |
| [Group=CTL] * [Frequency=150.00] | 46.886 | 0.0000 |
| [Group=CTL] * [Frequency=145.00] | 48.614 | 0.0000 |
| [Group=CTL] * [Frequency=140.00] | 49.511 | 0.0000 |
| [Group=CTL] * [Frequency=135.00] | 49.599 | 0.0000 |
| [Group=CTL] * [Frequency=130.00] | 48.995 | 0.0000 |
| [Group=CTL] * [Frequency=125.00] | 47.325 | 0.0000 |
| [Group=CTL] * [Frequency=120.00] | 47.244 | 0.0000 |
| [Group=CTL] * [Frequency=115.00] | 46.44 | 0.0000 |
| [Group=CTL] * [Frequency=110.00] | 45.478 | 0.0000 |
| [Group=CTL] * [Frequency=105.00] | 53.759 | 0.0000 |
| [Group=CTL] * [Frequency=100.00] | 42.199 | 0.0000 |
| [Group=CTL] * [Frequency=95.00] | 32.765 | 0.0000 |
| [Group=CTL] * [Frequency=90.00] | 29.164 | 0.0000 |
| [Group=CTL] * [Frequency=85.00] | 20.884 | 0.0000 |
| [Group=CTL] * [Frequency=80.00] | 14.238 | 0.0000 |
| [Group=CTL] * [Frequency=75.00] | 16.269 | 0.0000 |
| [Group=CTL] * [Frequency=70.00] | 28.321 | 0.0000 |
| [Group=CTL] * [Frequency=65.00] | 28.924 | 0.0000 |
| [Group=CTL] * [Frequency=60.00] | 16.061 | 0.0000 |
| [Group=CTL] * [Frequency=55.00] | 8.547 | 0.0030 |
| [Group=CTL] * [Frequency=50.00] | 6.851 | 0.0090 |
| [Group=CTL] * [Frequency=40.00] | 0.03 | 0.8620 |
| [Group=CTL] * [Frequency=35.00] | 2.92 | 0.0870 |
| [Group=CTL] * [Frequency=30.00] | 46.548 | 0.0000 |
| [Group=CTL] * [Frequency=25.00] | 316.619 | 0.0000 |
| [Group=CTL] * [Frequency=45.00] | 0a |  |

Supplemental Table 20: GEE for CFC MI statistics and Group x Frequency interactions at OML. The CTL peak at 40 Hz is used as a comparator (0a indicates statistical comparator).

|  | | |
| --- | --- | --- |
| Parameter | Hypothesis Test | |
|  | Wald Chi-Square | Sig. |
| [Group=FSE] * [Frequency=205.00] | 55.093 | 0.0000 |
| [Group=FSE] * [Frequency=200.00] | 44.015 | 0.0000 |
| [Group=FSE] * [Frequency=195.00] | 44.183 | 0.0000 |
| [Group=FSE] * [Frequency=190.00] | 49.157 | 0.0000 |
| [Group=FSE] * [Frequency=185.00] | 45.06 | 0.0000 |
| [Group=FSE] * [Frequency=180.00] | 51.637 | 0.0000 |
| [Group=FSE] * [Frequency=175.00] | 44.793 | 0.0000 |
| [Group=FSE] * [Frequency=170.00] | 48.083 | 0.0000 |
| [Group=FSE] * [Frequency=165.00] | 40.687 | 0.0000 |
| [Group=FSE] * [Frequency=160.00] | 37.832 | 0.0000 |
| [Group=FSE] * [Frequency=155.00] | 39.392 | 0.0000 |
| [Group=FSE] * [Frequency=150.00] | 28.996 | 0.0000 |
| [Group=FSE] * [Frequency=145.00] | 28.121 | 0.0000 |
| [Group=FSE] * [Frequency=140.00] | 29.492 | 0.0000 |
| [Group=FSE] * [Frequency=135.00] | 31.436 | 0.0000 |
| [Group=FSE] * [Frequency=130.00] | 32.838 | 0.0000 |
| [Group=FSE] * [Frequency=125.00] | 32.925 | 0.0000 |
| [Group=FSE] * [Frequency=120.00] | 31.874 | 0.0000 |
| [Group=FSE] * [Frequency=115.00] | 30.659 | 0.0000 |
| [Group=FSE] * [Frequency=110.00] | 29.575 | 0.0000 |
| [Group=FSE] * [Frequency=105.00] | 34.647 | 0.0000 |
| [Group=FSE] * [Frequency=100.00] | 31.025 | 0.0000 |
| [Group=FSE] * [Frequency=95.00] | 31.256 | 0.0000 |
| [Group=FSE] * [Frequency=90.00] | 28.86 | 0.0000 |
| [Group=FSE] * [Frequency=85.00] | 23.709 | 0.0000 |
| [Group=FSE] * [Frequency=80.00] | 18.335 | 0.0000 |
| [Group=FSE] * [Frequency=75.00] | 13.313 | 0.0000 |
| [Group=FSE] * [Frequency=70.00] | 12.315 | 0.0000 |
| [Group=FSE] * [Frequency=65.00] | 10.821 | 0.0010 |
| [Group=FSE] * [Frequency=60.00] | 10.429 | 0.0010 |
| [Group=FSE] * [Frequency=55.00] | 10.482 | 0.0010 |
| [Group=FSE] * [Frequency=50.00] | 10.443 | 0.0010 |
| [Group=FSE] * [Frequency=45.00] | 10.843 | 0.0010 |
| [Group=FSE] * [Frequency=40.00] | 9.153 | 0.0020 |
| [Group=FSE] * [Frequency=35.00] | 6.742 | 0.0090 |
| [Group=FSE] * [Frequency=30.00] | 1.561 | 0.2120 |
| [Group=FSE] * [Frequency=25.00] | 0.003 | 0.9550 |
| [Group=CTL] * [Frequency=205.00] | 62.805 | 0.0000 |
| [Group=CTL] * [Frequency=200.00] | 45.644 | 0.0000 |
| [Group=CTL] * [Frequency=195.00] | 42.63 | 0.0000 |
| [Group=CTL] * [Frequency=190.00] | 47.997 | 0.0000 |
| [Group=CTL] * [Frequency=185.00] | 40 | 0.0000 |
| [Group=CTL] * [Frequency=180.00] | 49.177 | 0.0000 |
| [Group=CTL] * [Frequency=175.00] | 39.789 | 0.0000 |
| [Group=CTL] * [Frequency=170.00] | 46.642 | 0.0000 |
| [Group=CTL] * [Frequency=165.00] | 39.82 | 0.0000 |
| [Group=CTL] * [Frequency=160.00] | 41.177 | 0.0000 |
| [Group=CTL] * [Frequency=155.00] | 46.473 | 0.0000 |
| [Group=CTL] * [Frequency=150.00] | 32.311 | 0.0000 |
| [Group=CTL] * [Frequency=145.00] | 31.528 | 0.0000 |
| [Group=CTL] * [Frequency=140.00] | 33.956 | 0.0000 |
| [Group=CTL] * [Frequency=135.00] | 36.39 | 0.0000 |
| [Group=CTL] * [Frequency=130.00] | 36.932 | 0.0000 |
| [Group=CTL] * [Frequency=125.00] | 35.581 | 0.0000 |
| [Group=CTL] * [Frequency=120.00] | 33.523 | 0.0000 |
| [Group=CTL] * [Frequency=115.00] | 31.05 | 0.0000 |
| [Group=CTL] * [Frequency=110.00] | 26.944 | 0.0000 |
| [Group=CTL] * [Frequency=105.00] | 31.636 | 0.0000 |
| [Group=CTL] * [Frequency=100.00] | 22.778 | 0.0000 |
| [Group=CTL] * [Frequency=95.00] | 24.505 | 0.0000 |
| [Group=CTL] * [Frequency=90.00] | 24.296 | 0.0000 |
| [Group=CTL] * [Frequency=85.00] | 21.426 | 0.0000 |
| [Group=CTL] * [Frequency=80.00] | 20.584 | 0.0000 |
| [Group=CTL] * [Frequency=75.00] | 14.997 | 0.0000 |
| [Group=CTL] * [Frequency=70.00] | 15.099 | 0.0000 |
| [Group=CTL] * [Frequency=65.00] | 13.65 | 0.0000 |
| [Group=CTL] * [Frequency=60.00] | 9.847 | 0.0020 |
| [Group=CTL] * [Frequency=55.00] | 7.135 | 0.0080 |
| [Group=CTL] * [Frequency=50.00] | 4.778 | 0.0290 |
| [Group=CTL] * [Frequency=45.00] | 1.138 | 0.2860 |
| [Group=CTL] * [Frequency=35.00] | 0.351 | 0.5530 |
| [Group=CTL] * [Frequency=30.00] | 5.319 | 0.0210 |
| [Group=CTL] * [Frequency=25.00] | 1.77 | 0.1830 |
| [Group=CTL] * [Frequency=40.00] | 0a |  |

Supplemental Table 21: GEE for CFC MI statistics and Group x Frequency interactions at MML. The CTL peak at 40 Hz is used as a comparator (Asterisk indicates statistical significance and 0a indicates statistical comparator).

|  | | |
| --- | --- | --- |
| Parameter | Hypothesis Test | |
|  | Wald Chi-Square | Sig. |
| [Group=FSE] * [Frequency=205.00] | 23.091 | 0.0000 |
| [Group=FSE] * [Frequency=200.00] | 9.261 | 0.0020 |
| [Group=FSE] * [Frequency=195.00] | 8.763 | 0.0030 |
| [Group=FSE] * [Frequency=190.00] | 12.012 | 0.0010 |
| [Group=FSE] * [Frequency=185.00] | 8.853 | 0.0030 |
| [Group=FSE] * [Frequency=180.00] | 13.604 | 0.0000 |
| [Group=FSE] * [Frequency=175.00] | 8.349 | 0.0040 |
| [Group=FSE] * [Frequency=170.00] | 10.507 | 0.0010 |
| [Group=FSE] * [Frequency=165.00] | 5.935 | 0.0150 |
| [Group=FSE] * [Frequency=160.00] | 5.028 | 0.0250 |
| [Group=FSE] * [Frequency=155.00] | 7.589 | 0.0060 |
| [Group=FSE] * [Frequency=150.00] | 2.013 | 0.1560 |
| [Group=FSE] * [Frequency=145.00] | 1.871 | 0.1710 |
| [Group=FSE] * [Frequency=140.00] | 2.298 | 0.1300 |
| [Group=FSE] * [Frequency=135.00] | 2.643 | 0.1040 |
| [Group=FSE] * [Frequency=130.00] | 2.550 | 0.1100 |
| [Group=FSE] * [Frequency=125.00] | 2.072 | 0.1500 |
| [Group=FSE] * [Frequency=120.00] | 1.326 | 0.2500 |
| [Group=FSE] * [Frequency=115.00] | 0.621 | 0.4310 |
| [Group=FSE] * [Frequency=110.00] | 0.228 | 0.6330 |
| [Group=FSE] * [Frequency=105.00] | 0.551 | 0.4580 |
| [Group=FSE] * [Frequency=100.00] | 0.150 | 0.6990 |
| [Group=FSE] * [Frequency=95.00] | 0.266 | 0.6060 |
| [Group=FSE] * [Frequency=90.00] | 0.367 | 0.5450 |
| [Group=FSE] * [Frequency=85.00] | 0.490 | 0.4840 |
| [Group=FSE] * [Frequency=80.00] | 1.041 | 0.3080 |
| [Group=FSE] * [Frequency=75.00] | 0.986 | 0.3210 |
| [Group=FSE] * [Frequency=70.00] | 1.852 | 0.1740 |
| [Group=FSE] * [Frequency=65.00] | 2.177 | 0.1400 |
| [Group=FSE] * [Frequency=60.00] | 2.181 | 0.1400 |
| [Group=FSE] * [Frequency=55.00] | 1.923 | 0.1660 |
| [Group=FSE] * [Frequency=50.00] | 1.952 | 0.1620 |
| [Group=FSE] * [Frequency=45.00] | 2.561 | 0.1100 |
| [Group=FSE] * [Frequency=40.00] | 4.249 | 0.0390 |
| [Group=FSE] * [Frequency=35.00] | 3.775 | 0.0520 |
| [Group=FSE] * [Frequency=30.00] | 0.001 | 0.9710 |
| [Group=FSE] * [Frequency=25.00] | 1.867 | 0.1720 |
| [Group=CTL] * [Frequency=205.00] | 12.058 | 0.0010 |
| [Group=CTL] * [Frequency=200.00] | 6.451 | 0.0110 |
| [Group=CTL] * [Frequency=195.00] | 6.369 | 0.0120 |
| [Group=CTL] * [Frequency=190.00] | 8.484 | 0.0040 |
| [Group=CTL] * [Frequency=185.00] | 6.985 | 0.0080 |
| [Group=CTL] * [Frequency=180.00] | 9.678 | 0.0020 |
| [Group=CTL] * [Frequency=175.00] | 7.507 | 0.0060 |
| [Group=CTL] * [Frequency=170.00] | 10.089 | 0.0010 |
| [Group=CTL] * [Frequency=165.00] | 8.154 | 0.0040 |
| [Group=CTL] * [Frequency=160.00] | 8.691 | 0.0030 |
| [Group=CTL] * [Frequency=155.00] | 11.320 | 0.0010 |
| [Group=CTL] * [Frequency=150.00] | 7.056 | 0.0080 |
| [Group=CTL] * [Frequency=145.00] | 7.824 | 0.0050 |
| [Group=CTL] * [Frequency=140.00] | 8.586 | 0.0030 |
| [Group=CTL] * [Frequency=135.00] | 8.082 | 0.0040 |
| [Group=CTL] * [Frequency=130.00] | 6.612 | 0.0100 |
| [Group=CTL] * [Frequency=125.00] | 5.056 | 0.0250 |
| [Group=CTL] * [Frequency=120.00] | 3.727 | 0.0540 |
| [Group=CTL] * [Frequency=115.00] | 2.434 | 0.1190 |
| [Group=CTL] * [Frequency=110.00] | 1.316 | 0.2510 |
| [Group=CTL] * [Frequency=105.00] | 0.829 | 0.3630 |
| [Group=CTL] * [Frequency=100.00] | 0.244 | 0.6210 |
| [Group=CTL] * [Frequency=95.00] | 0.134 | 0.7140 |
| [Group=CTL] * [Frequency=90.00] | 0.045 | 0.8310 |
| [Group=CTL] * [Frequency=85.00] | 0.000 | 0.9840 |
| [Group=CTL] * [Frequency=80.00] | 0.012 | 0.9110 |
| [Group=CTL] * [Frequency=75.00] | 0.034 | 0.8550 |
| [Group=CTL] * [Frequency=70.00] | 0.002 | 0.9610 |
| [Group=CTL] * [Frequency=65.00] | 0.007 | 0.9340 |
| [Group=CTL] * [Frequency=60.00] | 0.044 | 0.8330 |
| [Group=CTL] * [Frequency=55.00] | 0.082 | 0.7750 |
| [Group=CTL] * [Frequency=50.00] | 0.089 | 0.7650 |
| [Group=CTL] * [Frequency=45.00] | 0.235 | 0.6280 |
| [Group=CTL] * [Frequency=35.00] | 0.249 | 0.6180 |
| [Group=CTL] * [Frequency=30.00] | 0.429 | 0.5120 |
| [Group=CTL] * [Frequency=25.00] | 0.171 | 0.6800 |
| [Group=CTL] * [Frequency=40.00] | 0a |  |
